# Multifunctional solvent molecule design enables high-voltage Li-ion batteries

**DOI:** 10.1038/s41467-023-37999-4

**Published:** 2023-04-18

**Authors:** Junbo Zhang, Haikuo Zhang, Suting Weng, Ruhong Li, Di Lu, Tao Deng, Shuoqing Zhang, Ling Lv, Jiacheng Qi, Xuezhang Xiao, Liwu Fan, Shujiang Geng, Fuhui Wang, Lixin Chen, Malachi Noked, Xuefeng Wang, Xiulin Fan

**Affiliations:** 1 State Key Laboratory of Silicon Materials, School of Materials Science and Engineering, Zhejiang University, Hangzhou, 310027 China; 2 Shenyang National Laboratory for Materials Science, Northeastern University, Shenyang, 110819 China; 3 Beijing National Laboratory for Condensed Matter Physics, Institute of Physics, Chinese Academy of Sciences, Beijing, 100190 China; 4 Department of Chemical and Biomolecular Engineering, University of Maryland, College Park, MD USA; 5 State Key Laboratory of Clean Energy Utilization, School of Energy Engineering, Zhejiang University, Hangzhou, 310027 China; 6 Key Laboratory of Advanced Materials and Applications for Batteries of Zhejiang Province, Hangzhou, 310013 China; 7 Department of Chemistry, Bar-Ilan University, Ramat Gan, Israel; 8 Tianmu Lake Institute of Advanced Energy Storage Technologies Co. Ltd., Liyang, 213300 Jiangsu China

**Keywords:** Batteries, Batteries, Batteries

## Abstract

Elevating the charging cut-off voltage is one of the efficient approaches to boost the energy density of Li-ion batteries (LIBs). However, this method is limited by the occurrence of severe parasitic reactions at the electrolyte/electrode interfaces. Herein, to address this issue, we design a non-flammable fluorinated sulfonate electrolyte by multifunctional solvent molecule design, which enables the formation of an inorganic-rich cathode electrolyte interphase (CEI) on high-voltage cathodes and a hybrid organic/inorganic solid electrolyte interphase (SEI) on the graphite anode. The electrolyte, consisting of 1.9 M LiFSI in a 1:2 *v*/*v* mixture of 2,2,2-trifluoroethyl trifluoromethanesulfonate and 2,2,2-trifluoroethyl methanesulfonate, endows 4.55 V-charged graphite||LiCoO_2_ and 4.6 V-charged graphite||NCM811 batteries with capacity retentions of 89% over 5329 cycles and 85% over 2002 cycles, respectively, thus resulting in energy density increases of 33% and 16% compared to those charged to 4.3 V. This work demonstrates a practical strategy for upgrading the commercial LIBs.

## Abstract (web summary)

The parasitic reactions at the electrolyte/electrode interfaces inhibit the increase of the charging cut-off voltage and the improvement of energy density. Herein, the authors design multifunctional solvent molecules and propose a practical design principle to stabilize the electrolyte/electrode interfaces for high-voltage Li ion batteries.

## Introduction

Lithium-ion batteries (LIBs) with high energy density (>300 Wh kg^−1^) and long-term cycling performance are urgently needed for consumer electronics and electric vehicle applications^1,2^. However, the state-of-the-art LIBs, combining a Ni-rich LiNi_*x*_Co_*y*_Mn_1*−x−y*_O_2_ (NCM, *x* ≥ 0.6) or LiCoO_2_ (LCO) cathode and a graphite anode, can achieve a maximum energy density of ~250 Wh kg^−1^ when charged to ~4.3 V^3,4^. Elevating the charging cut-off voltage without altering the cathode and anode chemistry is a straightforward and efficient approach to increase both the reversible capacity and the working voltage, thus increasing the energy density of LIBs^5,6^.

Theoretically, an additional capacity of ~15 to 35% could be obtained after increasing the charging cut-off voltage of layered oxides cathodes from 4.3 to 4.7 V (vs. Li^+^/Li)^7^. Unfortunately, the elevation of the charging cut-off voltage is limited by the low oxidative stability of conventional carbonate electrolytes and their poor passivation capability on the delithiated cathodes, especially at >4.3 V (vs. Li^+^/Li)^8,9^. High parasitic reactions between the delithiated cathode and electrolyte at the high voltage will cause transition-metal dissolution from the cathode^10,11^ and also structural reconstruction^12^, thus degrading the battery performance. Besides, the SEI layer formed in the carbonate electrolytes with low ionic conductivity is detrimental to the cycling durability of LIBs^13^.

Extensive researches have been conducted to improve the cathode–electrolyte compatibility. Surface coating^14,15^ or doping^16^ the cathodes can reduce the incidence of side reactions between the electrolyte and the cathode. However, their performance enhancement is limited, especially at high cut-off voltages (>4.5 V) since these CEIs usually have a high interfacial resistance and lack self-healing capability. Electrolyte design is a promising solution to form CEIs in situ^17,18^ and helpful to resolve the aforementioned challenges. Hydrofluoroethers^19^, fluorobenzenes^20^, fluorinated sulfones^21^, and fluorinated carbonates^22–24^ have demonstrated better oxidation stability than their counterparts. Yet, the short shelf life of fluorinated electrolytes and the increase in interfacial impedance during usage^25,26^ remain major issues that restrict their applicability. Although additives^27,28^ can further protect the cathode by forming protective CEIs through sacrificial decomposition, the low amount of additive cannot maintain the interfacial stability during prolonged cycling because CEIs are in a dynamic process of formation, destruction, and repair. High-concentration electrolytes (HCEs)^29,30^, localized high-concentration electrolytes (LHCEs)^31,32^, and weakly-solvating electrolytes^33,34^ also improve the oxidative stability by forming inorganic-rich CEIs/SEIs. Yet, so far, the reported charging cut-off voltages in these electrolytes have been mostly limited to <4.5 V (vs. Li^+^/Li), and the fragile nature of anion-derived CEIs/SEIs^35^ limits the cycle life of LIBs.

Herein, to address the above challenges, we rationally create an electrolyte, namely 1.9 M lithium bis(fluorosulfonyl)imide (LiFSI) in a mixture of 2,2,2-trifluoroethyl trifluoromethanesulfonate (TTMS) and 2,2,2-trifluoroethyl methanesulfonate (TM) (1:2 *v*/*v*). The designed electrolyte forms thin and compact interphases (CEI and SEI) in situ; their high Li^+^ conductivity ensures fast Li^+^ intercalation/deintercalation kinetics while the CEI limits the reactivity of the cathode at high potentials. Post-mortem characterization analysis and theoretical simulations show that sulfur (S)-containing species (Li_2_SO_*x*_) in the interphases play a vital role in lowering the interfacial resistance. As a result, this electrolyte endows 4.55 V-charged graphite||LCO and 4.6 V-charged graphite||NCM811 batteries with capacity retentions of 89% over 5329 cycles and 85% over 2002 cycles, respectively. We also demonstrate that ampere-hour-level 4.55 V-charged graphite||LCO and 4.6 V-charged graphite||NCM811 pouch cells under lean electrolyte conditions (2 g (Ah)^−1^) exhibit better cycling stability and safety profile than their counterparts using conventional electrolytes.

## Results

### Design rationale for the sulfonate-based solvent family

**Fig. 1:**
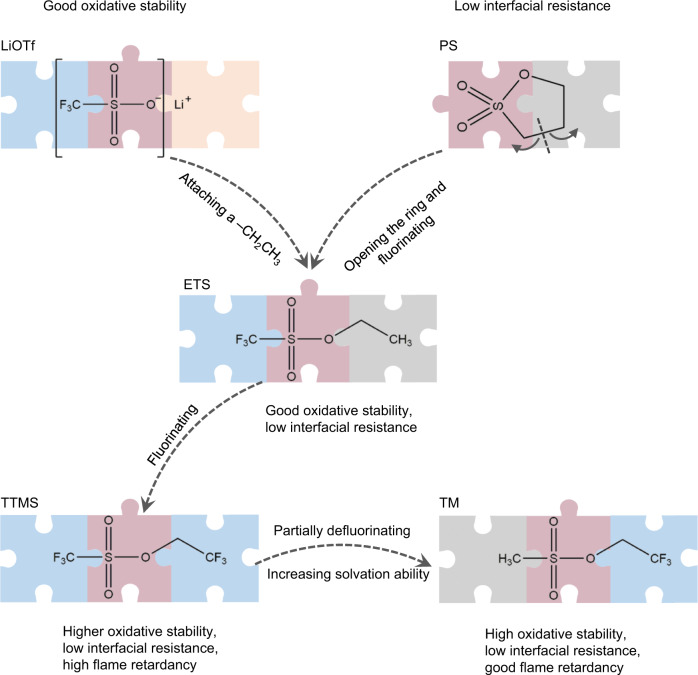
Design steps for multifunctional sulfonate-based solvents based on the stable LiOTf salt and the PS film-forming additive. By incorporating the functional groups into the LiOTf and PS and partial fluorination, TTMS and TM should inherit the merits of high oxidative stability, low interfacial resistance, and good flame retardancy.

Lithium trifluoromethanesulfonate (LiOTf), a stable lithium salt, possesses high oxidation stability^36^; 1,3-propane sultone (PS), one of the commercial film-forming additives, contributes to a low interfacial resistance and reducing gas generation in LIBs^37,38^ (Fig. 1). Through covalently attaching a –CH_2_CH_3_ to OTf or opening the PS ring and fluorinating, the synthesized ethyl trifluoromethanesulfonate (ETS) molecule should simultaneously exhibit good oxidation stability and be capable of lowering cell resistance and reducing gas generation. Introducing an electron-withdrawing –CF_3_ moiety on the *β*–carbon of ETS endows the resulting TTMS with higher anti-oxidation stability and flame retardancy, and it also reduces the negative effect on the solvation ability of O=S=O groups. To further enhance the solvation of Li-ions, the –CF_3_ moiety next to the O=S=O is replaced by a –CH_3_ moiety, thus forming TM. Based on this design rationale, TM should possess high oxidation stability, good Li-ion solvation ability, and the capability to form low-resistance films on both the anode and the cathode simultaneously. The stronger solvation power of TM is verified by the fact that its surface minimal value of electrostatic potential (*E*_min_) is lower (−29.6 kcal mol^−1^) than that of TTMS (−21.4 kcal mol^−1^) (Supplementary Fig. 1); a more negative *E*_min_ value represents a higher probability of coordinating with Li^+^^33^. Moreover, the high-voltage stability of the TM and TTMS is studied by oxidation potential calculations for the explicit presence of anion and solvents. As demonstrated in Supplementary Fig. 2, the solvents paired with FSI^–^ are endowed with a typical H-abstraction process under the oxidation reaction. The calculated oxidation potentials of TTMS and TM are 5.72 and 5.54 V, respectively. They are higher than those of conventional ethylene carbonate (EC, 5.31 V) and dimethyl carbonate (DMC, 5.36 V), indicating their superior oxidation stability. Besides, the good passivation ability of TTMS and TM is also confirmed by linear sweep voltammograms. The oxidation current density at 5.5 V of the cell containing the electrolyte of 1.9 M LiFSI/TTMS–TM (1:2 *v*/*v*) is only about 10% of that containing 1 M LiPF_6_/EC–DMC + 2%VC electrolyte (Supplementary Fig. 3). The ratio of Li salt to solvent (1.9 M LiFSI/TTMS–TM) was systematically screened to achieve a high oxidation stability and ionic conductivity (Supplementary Fig. 4). Therefore, the rational designed TTMS and TM have the potential to unlock the high-voltage capacity of cathodes and reduce the interfacial resistance, thus enhancing the energy density of commercial LIBs.

### Electrochemical performances of Li-ion full cells

The electrochemical performances of 4.55 V-charged graphite||LCO and 4.6 V-charged graphite||NCM811 full cells with the 1.9 M LiFSI/TTMS–TM electrolyte were evaluated. Two representative carbonate electrolytes, *id est*, 1 M LiPF_6_ in a mixture of ethylene carbonate and dimethyl carbonate (1 M LiPF_6_/EC–DMC, 1:1 *v*/*v*) and 1 M LiPF_6_/EC–DMC with 2 wt.% vinylene carbonate (1 M LiPF_6_/EC–DMC + 2%VC), were used as references. The use of high cut-off voltages, 4.55 V for graphite||LCO (>4.6 V for LCO *vs*. Li^+^/Li) and 4.6 V for graphite||NCM811 (>4.65 V for NCM811 *vs*. Li^+^/Li), represents a rigorous test for the electrolyte stability, because it magnifies the incidence of parasitic reactions on the catalytic cathode–electrolyte interface and requires effective cathode passivation.

**Fig. 2:**
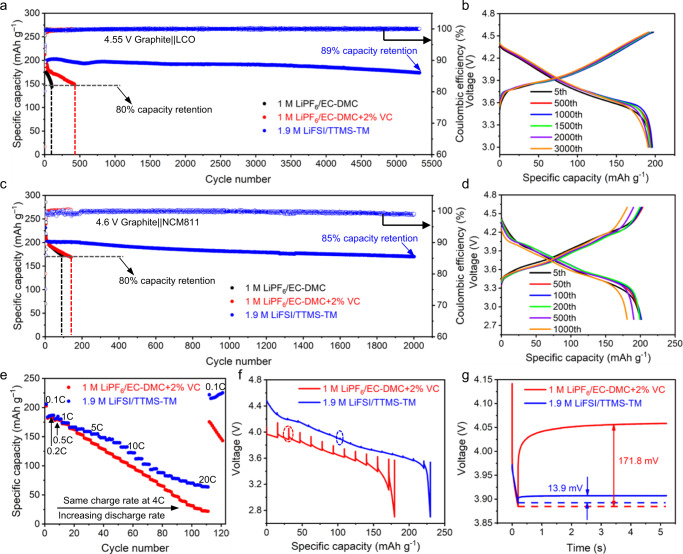
Electrochemical performances of Li-ion full cells using LCO and NCM811 as cathode materials under high-voltage operation. **a** Cycling stability of graphite | |LCO cells at a 4.55 V cut-off voltage with 1 C charge and 2 C discharge rates after three-cycles activation at C/10. **b** Corresponding charge–discharge curves of graphite | |LCO cells using the 1.9 M LiFSI/TTMS–TM electrolyte. **c** Cycling stability of graphite | |NCM811 cells at a 4.6 V cut-off voltage with 1 C charge and 2 C discharge rates after three-cycles activation at C/10. **d** Corresponding charge–discharge curves of graphite||NCM811 cells using the 1.9 M LiFSI/TTMS–TM electrolyte. **e** Rate performances of graphite||NCM811 cells using different electrolytes at a 4 C charge rate and various discharge rates (1 C–20 C). **f** Discharge voltage profiles of GITT measurements on the cells after 100 cycles at 1 C charge and 2 C discharge rates. All cells were cycled at a rate of C/3 with a 12-min pulse time and a 5-h rest time. **g** The magnified view of voltage relaxation process highlighted by ovals in (**f**).

All three graphite||LCO cells with different electrolytes exhibit a similar discharge specific capacity of 210 mAh g^−1^ at C/10 (Fig. 2a). The cell with the 1.9 M LiFSI/TTMS–TM electrolyte shows the best cycling stability (blue curve in Fig. 2a, and Fig. 2b), delivering a capacity retention of >89% after 5329 cycles, with an average Coulombic efficiency (CE) of >99.8%. Notably, the cycling stability of the graphite||LCO full cell is even higher than those of 4.6 V-charged Li||LCO (~93% capacity retention after 700 cycles, Supplementary Fig. 5) and Li||graphite (~90% capacity retention after 690 cycles, Supplementary Fig. 6) half cells, thanks to the high stability of the interfacial layers formed in situ on both graphite and LCO. In contrast, the graphite||LCO cell with the 1 M LiPF_6_/EC–DMC electrolyte (black curve in Fig. 2a) shows a rapid capacity drop, with only 80% capacity retention after 104 cycles. Although the VC additive mitigates capacity loss, the capacity of the 4.55 V-charged graphite||LCO cell still decays to ~80% of its original value over 424 cycles (red curve in Fig. 2a). The large overpotential increase in the referenced electrolytes (Supplementary Fig. 7) indicates severe parasitic reactions between the electrolyte and the electrode. The designed electrolyte is also beneficial to the electrochemical performance of a 4.55 V-charged graphite||LCO full cell cycled at a low rate of 0.5 C charge/1 C discharge (Supplementary Fig. 8).

The 1.9 M LiFSI/TTMS–TM electrolyte also improves the performance of a 4.6 V-charged graphite||NCM811 cell (Fig. 2c, d, and Supplementary Fig. 9), leading to a high capacity retention (>85% after 2002 cycles), a high discharge specific capacity (230 mAh g^−1^ at C/10), and high cycling stability at 60 °C (Supplementary Fig. 10). The cells with the referenced electrolytes (black and red curves in Fig. 2c, and Supplementary Fig. 11) show continuous capacity decay and their capacity drop to ~80% of their original value only after 93 and 141 cycles, respectively, which is consistent with previous reports^39^. Besides, the 1.9 M LiFSI/TTMS–TM electrolyte displays good wettability to the separator (Supplementary Fig. 12) and potential for low-temperature applications (Supplementary Fig. 13). Given its high compatibility with graphite anode and LCO/NCM811 cathodes at high cut-off voltage as well as the advantage of inhibiting Al dissolution via the formation of an AlO_*x*_F_*y*_/AlF_3_-like passivation layer (Supplementary Fig. 14), our designed 1.9 M LiFSI/TTMS–TM electrolyte is a promising option that could enable the realization of high-energy-density LIBs for 3 C (computer, communication, and consumer electronics) electronic devices and electric vehicles.

The cell with the 1.9 M LiFSI/TTMS–TM electrolyte also exhibits better rate performances than those with the referenced electrolyte (Fig. 2e and Supplementary Fig. 15). The cell with the referenced electrolyte shows rapid capacity decay and retains a low specific capacity (~20 mAh g^−1^) when the discharge rate is raised to 20 C. In contrast, the cell with the 1.9 M LiFSI/TTMS–TM electrolyte shows better rate capability, delivering a discharge specific capacity (~70 mAh g^−1^) 3.5 times than the baseline at 20 C. Its specific capacity then recovers to ~230 mAh g^−1^ when the rate returns to C/10. The well-performed rate performance benefits from the high Li^+^ transference number (*t*^+^) of the 1.9 M LiFSI/TTMS-TM electrolyte (0.56, Supplementary Fig. 16 and Supplementary Table 1), which is 1.75 times higher than that of the 1 M LiPF_6_/EC-DMC electrolyte.

Galvanostatic intermittent titration technique (GITT) was utilized to quantitatively study the kinetic changes of NCM811 cathode under high-voltage operation in different electrolytes (Fig. 2f, g). The overpotential of the cell with 1 M LiPF_6_/EC–DMC + 2%VC electrolyte (171.8 mV) is reduced by 92% when switching to the 1.9 M LiFSI/TTMS–TM electrolyte (13.9 mV), confirming the lower resistance of the interphase formed in the designed electrolyte. This overpotential drop is ascribed to accelerated reaction kinetics, which relates to the interfacial chemistries between the electrodes and electrolyte, in the presence of 1.9 M LiFSI/TTMS–TM electrolyte.

### Interfacial chemistry

**Fig. 3:**
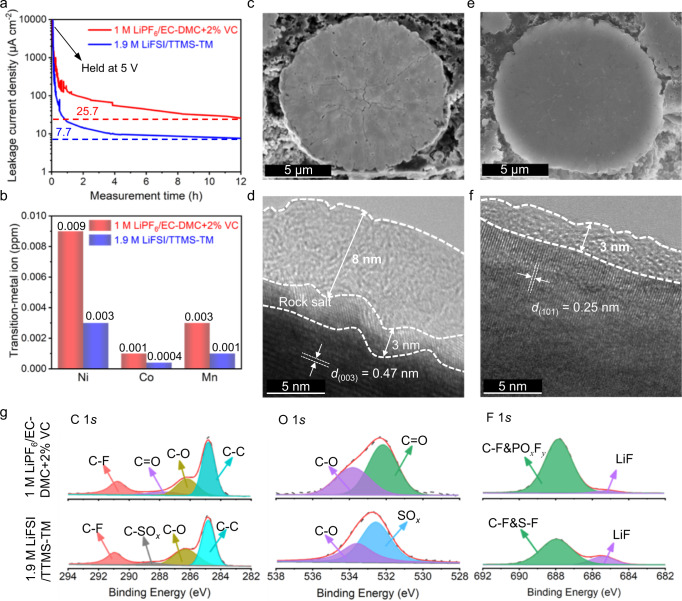
Oxidation stability of the designed electrolyte and interfacial chemistry of the NCM811 cathodes retrieved from the cycled full cells. **a** Leakage current density during a constant-voltage (5 V *vs*. Li^+^/Li) floating test of the NCM811 cathodes after 3 cycles in the indicated electrolyte. **b** Transition-metal (Ni, Co, and Mn) dissolution determined by inductively coupled plasma mass spectrometry (ICP-MS) after 100 cycles in the indicated electrolyte. **c**, **d** Cross-sectioned SEM images (**c**) and HRTEM images (**d**) of NCM811 cathodes retrieved from the graphite||NCM811 cells after 100 cycles in 1 M LiPF_6_/EC–DMC + 2%VC electrolyte. **e**, **f** Cross-sectioned SEM images (**e**) and HRTEM images (**f**) of NCM811 cathodes retrieved from the graphite||NCM811 cells after 100 cycles in 1.9 M LiFSI/TTMS–TM electrolyte. **g** XPS profiles of the NCM811 cathode surface retrieved from the graphite||NCM811 cells after 100 cycles in the indicated electrolyte.

To understand the origin of the improvement in high-voltage electrochemical performances, we evaluated the oxidation stability of the electrolytes on the NCM811 cathode. In the aggressive floating tests at 5 V with a 12-h holding time (Fig. 3a), the leakage current density of the cell with the 1.9 M LiFSI/TTMS–TM electrolyte is 7.7 μA cm^−2^, corresponding to ~30% of that with the 1 M LiPF_6_/EC–DMC + 2%VC electrolyte (25.7 μA cm^−2^). The lower float-test leakage current density in the 1.9 M LiFSI/TTMS–TM electrolyte demonstrates that fewer side reactions occur on the cathode surface, confirming the higher oxidation stability of the designed electrolyte as compared with the references. At high potentials, the parasitic reactions between the delithiated cathode and the electrolyte accelerate the transition-metal dissolution, which is considered one of the main failure mechanisms for high-energy LIBs^17,40^. The reduced occurrence of side reactions between the 1.9 M LiFSI/TTMS–TM electrolyte and the delithiated cathode effectively suppresses transition-metal dissolution (Fig. 3b) and the cross-talk between the CEI and SEI (Supplementary Fig. 17 and Supplementary Table 2), which contributes to sustaining the stability of the cell performance.

To further understand the well-performed electrochemical performances of the high-voltage NCM811 in our electrolyte, we investigated the structural evolution of the cathode materials and interphases upon cycling by cross-sectional scanning electron microscopy (SEM), high-resolution transmission electron microscopy (HRTEM), and X-ray photoelectron microscopy (XPS). The NCM811 cathode retrieved from graphite||NCM811 cells cycled with the referenced electrolyte presents extensive microcracks extending from the core to the surface along the grain boundaries of the primary particles (Fig. 3c and Supplementary Fig. 18b, c). These cracks are induced by serious parasitic reactions between the acidic carbonate electrolytes and the catalytic cathodes. The anisotropic expansion of NCM811 is also evidenced by the inconsistent peak shifts in the XRD patterns (Supplementary Fig. 19). In addition, a ~3 nm nonuniform rock-salt structure and a ~8 nm CEI layer are detected on the NCM811 cathode retrieved from the graphite||NCM811 cells cycled with the 1 M LiPF_6_/EC–DMC + 2%VC electrolyte (Fig. 3d). These large cracks and an inadequate CEI promote side reactions and capacity fading^41^. On the contrary, the NCM811 retrieved from the graphite||NCM811 cells cycled with the 1.9 M LiFSI/TTMS–TM electrolyte presents a well-structured morphology with few visible microcracks and little change in its crystalline structure (Fig. 3e, Supplementary Fig. 18d, and Supplementary Fig. 19). A well-defined and thin CEI layer (~3 nm) is present on the surface of NCM811 (Fig. 3f) and LCO (Supplementary Fig. 20) cathodes.

The CEI components are also different. XPS analysis of the CEI formed in the 1 M LiPF_6_/EC–DMC + 2%VC electrolyte (Fig. 3g) shows strong signals attributed to C–C (284.8 eV), C–O (286.5 eV) and C=O (287.7 eV)^31,42^ in the C 1*s* spectrum, indicating the decomposition of the carbonate electrolyte. The C–F (289.9 eV) peak corresponds to the polyvinylidene fluoride binder in the cathodes. Furthermore, the decomposition of LiPF_6_ is confirmed by the presence of LiF (685.2 eV) and PO_*x*_F_*y*_ (687.8 eV) in the F 1 *s* spectrum. For the CEI formed in the 1.9 M LiFSI/TTMS–TM electrolyte, the presence of SO_*x*_ (532.5 eV in O 1*s* spectrum, and 169.2 eV in S 2*p* spectrum, Supplementary Fig. 21)^43^ indicates the decomposition of the electrolyte. The low content of C–C and C–O organic species, and high content of LiF show the formation of a thin and inorganic-rich CEI in the 1.9 M LiFSI/TTMS–TM electrolyte.

These results prove that the 1.9 M LiFSI/TTMS–TM electrolyte not only possesses superior oxidation stability for high-voltage operation of LIBs, but also plays a vital role in passivating the catalytic cathode by generating a protective inorganic-rich CEI. The latter eliminates direct contact between the active cathode and the electrolyte, thus effectively preventing the continuous decomposition of the electrolyte and the intergranular cracking of the NCM811 particles. Such inhibition of side reactions between the electrolyte and the catalytic cathode leads to improved performance at high voltages.

**Fig. 4:**
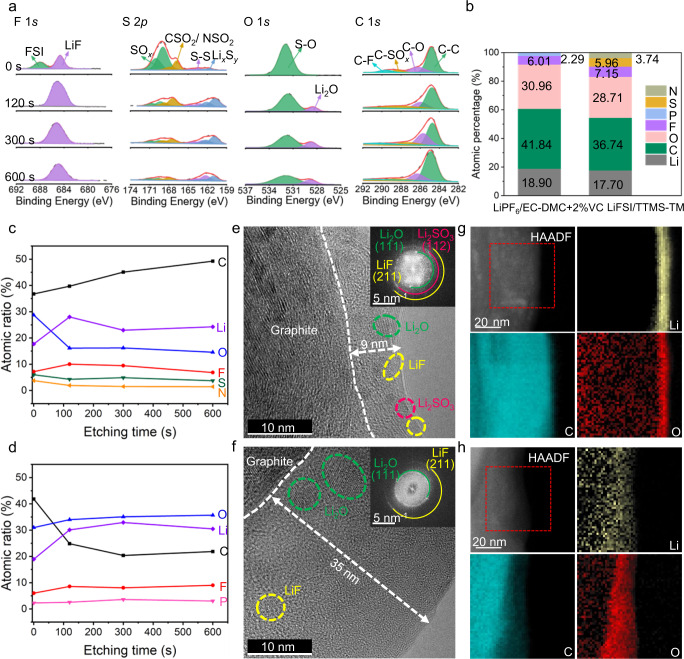
Interfacial chemistry of graphite anodes retrieved from full cells cycled with different electrolytes. **a** XPS depth profiles of F 1*s*, S 2*p*, O 1*s*, and C 1*s* for graphite anodes retrieved from full cells cycled with the 1.9 M LiFSI/TTMS–TM electrolyte at a sputtering time of 0, 120, 300, and 600 s. **b** Relative content of the detected elements on the surface of graphite anodes retrieved from full cells cycled with indicated electrolytes. **c**, **d** Atomic concentration at different depths of SEI formed in 1.9 M LiFSI/TTMS–TM electrolyte (**c**) and 1 M LiPF_6_/EC–DMC + 2%VC electrolyte (**d**). **e**, **f** Cryo-TEM images of graphite anodes after 100 cycles in 1.9 M LiFSI/TTMS–TM electrolyte (**e**) and 1 M LiPF_6_/EC–DMC + 2%VC electrolyte (**f**). **g**, **h** Corresponding EELS mappings of graphite anodes retrieved from full cells cycled with the 1.9 M LiFSI/TTMS–TM electrolyte (**g**) and the 1 M LiPF_6_/EC–DMC + 2%VC electrolyte (**h**).

An effective SEI formed in situ by electrolyte decomposition on the graphite anode is a pre-requisite for the proper function of LIBs^13^, which rules out a myriad of anodically stable solvents with low salt concentrations as LIBs electrolytes, including phosphates, sulfones, nitriles, and some ionic liquids^17,18^. To characterize the SEI formed on the graphite anode, XPS (Fig. 4a–d and Supplementary Fig. 22) and cryogenic transmission electron microscopy (Cryo-TEM, Fig. 4e–h)^44^. were performed. The component and elemental distribution of the SEIs formed in the two kinds of electrolytes are distinct. As shown by XPS results, a high content of LiF prevails throughout the SEI formed in the 1.9 M LiFSI/TTMS–TM electrolyte, and some Li_2_O is observed in the inner layer (Fig. 4a, b). In the S 2*p* spectrum, the intensity of the strong SO_*x*_ peak in the outer layer, originating from the reduction of TTMS/TM, continually decreases as the etching depth increases. Meanwhile, the peak of Li_*x*_S_*y*_ (161.3 eV), originating from the decomposition of LiFSI^45^, gradually increases. These results indicate that LiFSI is decomposed preferentially over TTMS/TM. The amount of C component increases consistently with the etching time (Fig. 4c) because the etching beam penetrated the thin SEI and reached the bulk graphite. In contrast, the C content in the thicker SEI formed in the referenced electrolyte (Fig. 4d and Supplementary Fig. 22) decreases as the F content increases over an etching time of 120 s. This elemental distribution agrees well with the classic mosaic SEI model. It indicates the SEI contains an outer layer mainly consisting of organic species at high oxidation states (mainly Li alkyl carbonates) and an inner layer consisting of inorganic compounds (mainly LiF and Li_2_O) that are more resistant to reduction^13^. The difference in SEI structure was further confirmed by cryo-TEM (Fig. 4e, f, and Supplementary Fig. 23) and electron energy loss spectroscopy (EELS) mapping (Fig. 4g, h). A thin (~9 nm) and homogeneous SEI (Fig. 4e, g) is generated on the graphite anode retrieved from the graphite||NCM811 cells cycled with the 1.9 M LiFSI/TTMS–TM electrolyte; it contains several uniformly dispersed inorganic species (Li_2_SO_3_, LiF and Li_2_O), which is consistent with the above XPS results. In contrast, the graphite anode retrieved from the full cells cycled with the referenced electrolyte is covered by a thick (~35 nm) and nonuniform SEI (Fig. 4f, h) consisting of an abundance of amorphous components and a low number of inorganic species (Li_2_O and LiF). Meanwhile, the post-mortem analysis after different cycles in cell chemistries (Supplementary Fig. 24, Supplementary Fig. 25, and Supplementary Fig. 26) indicates that the electrode/electrolyte interface is well sustained in the cell with the 1.9 M LiFSI/TTMS-TM electrolyte once it is formed, ensuring the stable electrochemical performance.

**Fig. 5:**
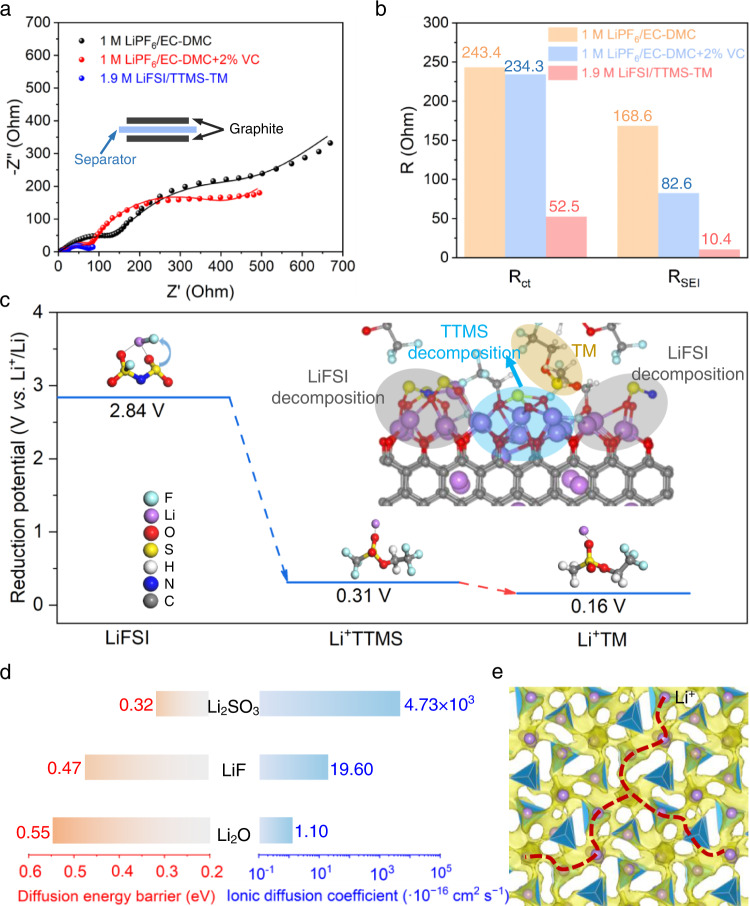
Fast kinetics ensured by the 1.9 M LiFSI/TTMS–TM electrolyte. **a** EIS of the symmetric cells using graphite anodes retrieved from the graphite||NCM811 cells after 1000 cycles in different electrolytes. **b** The resistance obtained by fitting with the equivalent circuit shown in Supplementary Fig. 27. **c** Calculated reduction potentials (vs. Li^+^/Li) and reduced complexes of LiFSI, TTMS, and TM with SMD solvation model. Inset is the simulated reduction products of the 1.9 M LiFSI/TTMS–TM electrolyte on the lithiated graphite anode. **d** Fitted Li^+^ diffusion coefficients at room temperature and diffusion energy barriers of the main reduction products (Li_2_SO_3_, LiF, and Li_2_O) in 1.9 M LiFSI/TTMS–TM electrolyte. **e** Simulated possible Li^+^ diffusion pathways in bulk Li_2_SO_3_ by the BVEL method.

Thanks to the thin and compact SEI, the SEI layer resistance (*R*_SEI_) of the cycled graphite anodes retrieved from full cells after 1000 cycles in the 1.9 M LiFSI/TTMS–TM electrolyte is only about 6% of that in the 1 M LiPF_6_/EC–DMC + 2%VC electrolyte (Fig. 5a, b, and Supplementary Fig. 27). The lower *R*_SEI_ indicates higher Li^+^ diffusion kinetics through the SEI. Density functional theory (DFT) calculations were performed to further reveal the underlying mechanism behind the fast kinetics enabled by the 1.9 M LiFSI/TTMS–TM electrolyte. Reduction potential calculations for the explicit presence of Li^+^ and solvents indicate that LiFSI becomes reductively unstable below 2.84 V (*vs*. Li^+^/Li) (Fig. 5c), which is substantially higher than the reduction potential of TTMS (0.31 V *vs*. Li^+^/Li) and TM (0.16 V *vs*. Li^+^/Li). The electrolyte decomposition simulations (ab initio molecular dynamics (AIMD) simulation of electrolyte on the lithiated graphite anode in the inset of Fig. 5c and DFT calculation of possible reduction pathways of the TTMS in Supplementary Fig. 28) show that the preferred reduction of FSI^–^ and TTMS generates sufficient inorganic compounds, including Li_2_SO_*x*_, LiF, and Li_2_O, to form a SEI at graphite-electrolyte interface. The Li^+^ diffusion coefficient and diffusion energy barrier in Li_2_SO_3_, LiF, and Li_2_O (the main products of the 1.9 M LiFSI/TTMS–TM electrolyte reductive decomposition, as determined by XPS, TEM and theoretical calculations) were calculated by the AIMD (Fig. 5d and Supplementary Fig. 29). Li_2_SO_3_ shows the lowest diffusion energy barrier (0.32 eV) and highest room-temperature ionic diffusion coefficient (4.73 × 10^−13^ cm^2^ s^−1^), which ensures the rapid transport of Li^+^ through the SEI, kinetically preventing the sustained reduction of the electrolyte. Li^+^ diffusion pathways (Fig. 5e and Supplementary Fig. 30) obtained by the bond valence energy landscape (BVEL) method^46^ also indicate a continuous three-dimensional (3D) Li^+^ percolation network in Li_2_SO_3_, which is in line with the ionically permeable SEI formed in electrolytes with PS additives^37,38^. Therefore, the thin Li_2_SO_*x*_-containing SEI formed on graphite in 1.9 M LiFSI/TTMS–TM electrolyte contributes to the improved rate capability and long life of LIBs.

### Pouch cell performance and safety assessment

**Fig. 6:**
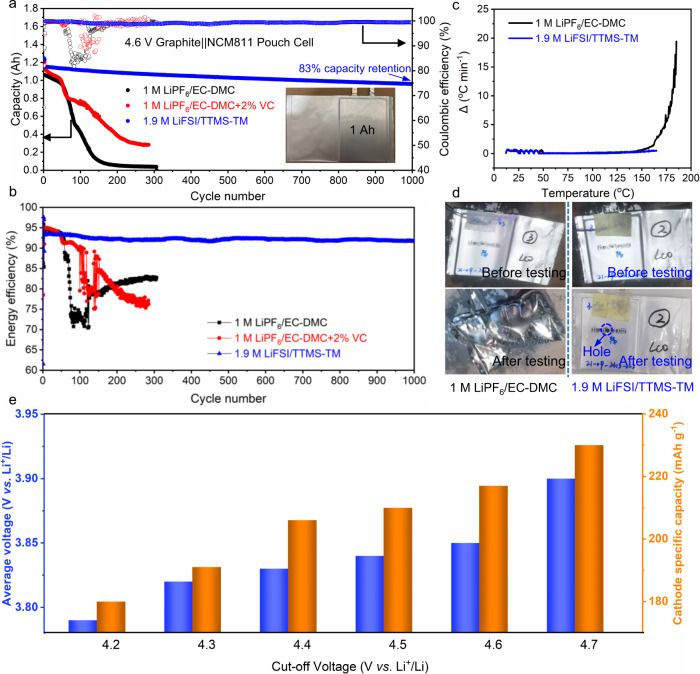
Practical application of the 1.9 M LiFSI/TTMS–TM electrolyte in pouch cells. **a** Cycling performance of 1-Ah graphite||NCM811 pouch cells. **b** Energy efficiency of 1-Ah graphite||NCM811 pouch cells. **c** ARC test for fully charged graphite||NCM811 pouch cells. **d** Optical morphology of graphite||LCO pouch cells before and after the nail penetration test. **e** Average voltage and cathode specific capacity of graphite||NCM811 pouch cells at different charging cut-off voltages. The pouch cell tests were carried out at 0.5 C charge and 1 C discharge in the voltage range from 3 to 4.6 V.

Most recent studies on high-voltage batteries use a low-loading cathode or an excessive amount of electrolyte (or both) while ignoring the effects of the aggressive conditions in practical cells on the battery performances^47^. In this work, we used high-loading cathodes (2.85 mAh cm^–2^), a low negative-to-positive capacity ratio (N/P, ~1), and a limited amount of electrolyte (low electrolyte/capacity ratio of 2 g (Ah)^–1^) to build practical 1-Ah pouch cells. Such harsh conditions are destructive for carbonate electrolytes. All 4.6 V-charged graphite||NCM811 (black and red curves in Fig. 6a, and Supplementary Fig. 31a) and 4.55 V-charged graphite||LCO pouch cells (black curve in Supplementary Fig. 32a, and Supplementary Fig. 32b) with the referenced electrolytes show rapid capacity decay (80% retention) within 100 cycles because of uncontrolled side reactions. In contrast, the 4.6 V-charged graphite||NCM811 pouch cell cycled in the 1.9 M LiFSI/TTMS–TM electrolyte still retains 83% of its original capacity after 1000 cycles (blue curve in Fig. 6a), with an energy efficiency of >93% (Fig. 6b). Besides, the 1.9 M LiFSI/TTMS–TM electrolyte also endows the 4.55 V-charged graphite||LCO pouch cell with well-performed cycling performance: the cell retains 80% of its original capacity after 1400 cycles (Supplementary Fig. 32).

The 1.9 M LiFSI/TTMS–TM electrolyte improves the safety profile of the studied high-voltage LIBs, as demonstrated by an accelerating rate calorimetry (ARC) test of 4.6 V-charged graphite||NCM811 pouch cells and a nail penetration test of 4.55 V-charged graphite||LCO pouch cells. While the cell with the carbonate electrolyte shows a low runaway temperature of ~150 °C (Fig. 6c), the cell with the 1.9 M LiFSI/TTMS–TM electrolyte shows no obvious sign of thermal runaway up to at least 170 °C. In the nail penetration test, the flammable carbonate electrolyte causes the pouch cell to burst into flame in 4 s (Fig. 6d, left and Supplementary Movie 1). In contrast, the pouch cell with the 1.9 M LiFSI/TTMS–TM electrolyte passes the test successfully (Fig. 6d, right and Supplementary Movie 2). This could be due to the superior flame retardancy of the 1.9 M LiFSI/TTMS–TM electrolyte (Supplementary Fig. 33 and Supplementary Movie 3) as compared with the carbonate electrolyte (Supplementary Movie 4).

Furthermore, the gas expansion rates (*ER*s) of graphite||NCM811 pouch cells after 300 cycles in 1 M LiPF_6_/EC–DMC and 1 M LiPF_6_/EC–DMC + 2%VC electrolytes reach 73% and 69% (Supplementary Fig. 34), respectively, whereas the cell expands by only 20% after 1000 cycles in the 1.9 M LiFSI/TTMS–TM electrolyte. Similar improvements are achieved with graphite||LCO pouch cells (Supplementary Fig. 35), demonstrating the intrinsic safety of the 1.9 M LiFSI/TTMS–TM electrolyte compared with conventional carbonate electrolytes under such aggressive conditions.

Increasing the charging cut-off voltage improves the discharge capacity and energy density of LIBs^17^. Although the average voltages of 4.6 V-charged graphite||NCM811 and 4.55 V-charged graphite||LCO pouch cells are only 0.08 V and 0.15 V higher than those of their 4.3 V-charged counterparts (Fig. 6e and Supplementary Fig. 36), the reversible cathode capacities are increased by 16% and 33% (Supplementary Tables 3–5), respectively. The 1-Ah 4.6 V-charged graphite||NCM811 and 4.55 V-charged graphite||LCO pouch cells with 1.9 M LiFSI/TTMS–TM electrolyte deliver an energy density exceeding 300 Wh kg^–1^ (Supplementary Table 6,7). Higher energy density (338 Wh kg^–1^ for graphite||NCM811 and 363 Wh kg^–1^ for graphite||LCO) could be achieved when decupling the pouch cell size, making the 1.9 M LiFSI/TTMS–TM electrolyte a promising candidate for the next generation of LIBs electrolytes.

## Discussion

### Design principles for high-voltage LIBs electrolyte

**Fig. 7:**
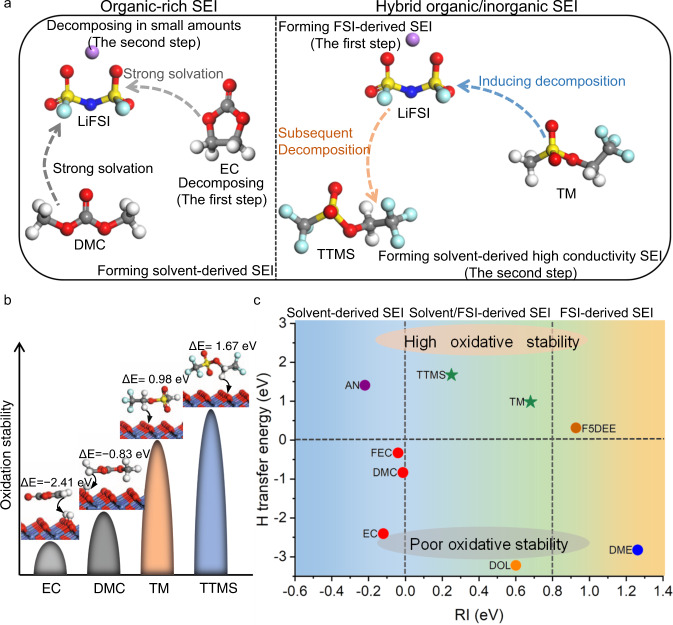
Design principles for high-voltage LIBs electrolyte. **a** Different kinds of SEIs formed on graphite anodes. **b** Oxidation stability of different solvents at fully charged NCM811 surface. **c** Design principle of high-voltage LIB electrolytes. On the vertical axis, spontaneous H-abstraction occurs in the electrolytes below the horizontal dashed line (corresponding to 0 eV), whereas non-spontaneous H-abstraction occurs in the electrolytes above the same line. On the horizontal axis, the three regions separated by vertical dashed lines indicate a solvent-derived SEI, solvent/FSI^–^-derived hybrid SEI, and FSI^–^-derived SEI. The *RI* of DME is calculated by the simulation of LiFSI/1.2DME–1,1,2,2-tetrafluoroethyl-2,2,3,3-tetrafluoropropylether (TTE) electrolyte, representing localized high-concentration electrolytes.

For LIBs, the in situ interfacial chemistry is critical in determining the electrochemical performance. It depends on the competition between the solvent molecules and the anions for the reaction on the electrode surface. For instance, a solvent-derived organic-rich SEI is prevalent in conventional carbonate electrolytes^13^ (Fig. 7a and Supplementary Fig. 37), but its high-resistance nature and poor stability^48^ cannot support long-term Li^+^ intercalation/deintercalation into/from graphite. On the contrary, in HCEs and LHCEs, an anion-derived inorganic-rich SEI is dominant (Supplementary Fig. 38)^29,49^; yet, this inorganic-rich SEI is still unable to prevent the capacity decay owing to its fragile nature^35^.

The compromised modulation of the electrolyte composition makes a hybrid organic/inorganic SEI with high ion conductivity possible. The redox reactivity of the anion and the solvent in electrolytes plays an important role in the composition of SEI. Unfortunately, the highest occupied molecular orbitals (HOMOs) and the lowest unoccupied molecular orbitals (LUMOs), derived from approximated electronic structure theory, cannot precisely determine actual redox reactivity of molecules^50^. Oxidation potentials and reduction potentials are more useful in that regard, but they are difficult to be accurately calculated due to the influences of solvation environment and molecule polarization^51^. To date, no reliable parameters have been reported to describe the redox reactivity of the anion and the solvent in electrolytes. Given this, we propose a reactivity index (*RI*), defined as the difference between the projected density of states (PDOS) centers of the anion (here, FSI^–^) and the solvent (Supplementary Figs. 39 and 40, and Supplementary Table 8); *RI* represents the reduction activity of the solvent compared to that of the anion on the anode (detailed information is provided in the theoretical calculation section). The oxidative stability of the electrolyte, indicated by H-abstraction induced free-energy change^52^ (Fig. 7b and Supplementary Fig. 41), defines the high-voltage limit at the cathode side. The H-transfer reaction energies of TTMS (1.67 eV) and TM (0.98 eV) are much higher than those of conventional EC (–2.41 eV) and DMC (–0.83 eV), as shown in Fig. 7b.

Based on the above discussion, we propose a design principle for high-voltage LIBs electrolytes. In Fig. 7c, the graph is divided into three regions according to *RI* values (along the *x* axis) and into two regions according to H**-**transfer energy (along the *y* axis). We use the FSI^–^ as a benchmark (corresponding to *RI* = 0 eV) to define the relative reactivity of various solvents. The electrolytes in the blue region (*RI* < 0 eV), based on solvents such as acetonitrile (AN), fluoroethylene carbonate (FEC), DMC, and EC, tend to form solvent-derived SEIs because the reduction of these strongly polar solvents usually dominates the interfacial chemistry. The electrolytes in the yellow region (*RI* tends to >0.8 eV), such as HCEs, LCHEs and weakly solvating electrolytes (e.g., F5DEE), tend to form inorganic-rich SEIs, because of the decomposition of FSI^–^ in contact ion pairs and aggregates. Finally, the electrolytes in the green region (*RI* > 0 eV and *RI* < 0.8 eV), based on solvents such as 1,3-dioxolane (DOL), TTMS, and TM, tend to form FSI^–^/solvent-derived hybrid organic/inorganic SEIs, because of the comparable reactivity between the anion and the solvent. In particular, TM with its low binding energy with Li^+^ (Supplementary Fig. 42), promotes the decomposition of FSI^–^ by introducing FSI^–^ to the primary solvation sheath. TTMS, with a higher reduction potential than TM (Fig. 5c), is subsequently decomposed. Therefore, a hybrid organic/inorganic SEI is formed via reactivity regulation between FSI^–^, TM, and TTMS. These results verify the feasibility of our *RI* parameter for electrolyte designing (Fig. 7c). Although DOL regulates the SEI well on graphite, its poor compatibility with the cathodes due to its spontaneous H-abstraction behavior rules it out as a solvent for high-voltage LIB electrolytes. This conclusion is also confirmed by the fact that the 1.9 M LiFSI/TM electrolyte enables the cycling of 4.6 V-charged graphite||NCM811 batteries with good stability (Supplementary Fig. 43).

Therefore, the formulation of electrolytes with superior SEI-forming capability and high oxidative stability can be achieved by choosing solvents in the region where TM and TTMS are situated, as schematically demonstrated in Supplementary Fig. 44. This design principle, along with the derived 1.9 M LiFSI/TTMS–TM electrolyte, opens a research avenue for high-voltage and long-cycling LIBs.

In summary, by fusing the merits of additive (PS) and lithium salt (LiOTf) into solvents, we report a fluorinated sulfonate electrolyte for application in high-voltage and long-cycle LIBs. The proposed 1.9 M LiFSI/TTMS–TM electrolyte stabilizes the NCM811 and LCO cathodes under aggressive high-voltage conditions by suppressing parasitic reactions (electrolyte oxidation, transition-metal dissolution, gas evolution, etc.), and enables fast Li^+^ intercalation/deintercalation kinetics on the graphite anode by forming S-containing species (Li_2_SO_*x*_). Furthermore, the non-flammable electrolyte extends the limits for high-voltage batteries, allowing configurations such as 4.55 V-charged graphite||LCO and 4.6 V-charged graphite||NCM811 to remain stable over thousands of cycles while also maintaining a good safety profile. We also proposed a design principle for high-voltage electrolytes: (1) they should possess high oxidation stability (high H-transfer energy and good passivation ability, i.e., it should form a protective inorganic-rich CEI); (2) the reactivity between the lithium salt and the solvent should be comparable (*RI* > 0 eV but close to 0 eV), to generate a hybrid organic/inorganic SEI with high ion conductivity on the graphite anode. Our findings provide a simple but effective method to formulate electrolytes that improves the energy density of commercial LIBs.

## Methods

### Materials

NCM811, LCO, graphite, and carbon black were purchased from Hefei Kejing Materials Technology Co., Ltd. Lithium metal foil (thickness: 450 μm, diameter: 15.8 mm) was purchased from China Energy Lithium Co., Ltd. The stainless steel 2032-type battery shell and Cu/Al current collector were purchased from Guangdong Canrd New Energy Technology Co., Ltd. Celgard 2325 battery separator (thickness: 16 μm, diameter: 19 mm) was bought from Hefei Kejing Material Technology Co., Ltd. Polyvinylidene fluoride (PVDF), N-methyl pyrrolidone (NMP), lithium hydroxide (LiOH), polyacrylic acid (PAA), LiFSI (99%), LiPF_6_ (99%), 2,2,2-trifluoroethanol, dichloromethane, triethylamine, and sodium bicarbonate were purchased from Aladdin Chemistry Co. Ltd. Methanesulphonyl chloride was purchased from Alfa Aesar. Trifluoromethanesulfonate was purchased from MolMall Sarl. EC, DMC, and vinylene carbonate (VC) were purchased from Duoduo Chem Co., Ltd. The solvents and Li salts were all battery grade. All solvents were dried by molecular sieves to a residual water concentration of <10 ppm (measured by Karl–Fisher titration). Molarity (M, moles of salt in liters of solution, in mol L^−1^) is used to denote the salt concentration in the electrolyte. No additive was added to the 1.9 M LiFSI/TTMS–TM electrolyte.

### Synthesis

2,2,2-trifluoroethyl methanesulfonate (TM)^53^: 2,2,2-trifluoroethanol (1 equiv.) was mixed with dichloromethane in a flask placed in a water bath at 5 °C and stirred for 20 min. Then, triethylamine was added into the above mixture dropwise and stirred for another 10 min. Methanesulphonyl chloride (1.5 equiv.) in dichloromethane was added dropwise to the stirring solvent mixture. The flask was removed from the 5 °C bath and the mixture was stirred at room temperature for overnight. The reaction mixture was slowly added to a 5% sodium bicarbonate solution and stirred for 20 min. After that, the dichloromethane layer was separated, and the aqueous layer was extracted with dichloromethane. The organic extracts were dried and concentrated to afford the product yield of 90%. The product was moved to an argon-filled glovebox for further use.

2,2,2-trifluoroethyl trifluoromethanesulfonate (TTMS)^54^: Trifluoromethanesulfonate (12.5 mL, 44.3 mmol) and 2,2,2-trifluoroethanol (6.25 mL, 62.4 mmol) were mixed and stirred for 30 min at room temperature under nitrogen before being refluxed at 95 °C. After 4 h, the mixture was allowed to cool to room temperature and the excess of 2,2,2-trifluoroethanol was distilled, affording the target molecule as a colorless liquid. Yield: 85%. The product was moved to an argon-filled glovebox for further use.

The nuclear magnetic resonance (NMR) spectra for TTMS and TM solvents (Supplementary Fig. 45) indicate that there are no other impurities in TTMS and TM solvents except for trace amounts of water.

### Preparation of electrodes, electrolytes, and cells

Two kinds of referenced electrolytes (1 M LiPF_6_/EC–DMC and 1 M LiPF_6_/EC–DMC + 2%VC) were formulated by adding LiPF_6_ into an EC:DMC mixture solvent (1:1 by vol.) before stirring to ensure complete dissolution. The 1.9 M LiFSI/TTMS–TM electrolyte was obtained by dissolving LiFSI into a TTMS–TM mixture solvent (1:2 by vol.). All the above processes were carried out in a glovebox filled with purified argon, where the moisture and oxygen contents were <0.01 ppm. For coin cells, NCM811 and LCO electrode sheets were prepared by casting as-prepared NMP slurry (active materials: PVDF: carbon black = 8: 1: 1, weight ratio) on Al foil and then dried in oven at 80 °C for 12 h under vacuum. The coating thickness of the slurry was controlled within the range of 150–200 μm. After that, the electrodes were punched into discs (diameter: 12 mm, mass loading: ~7.1 mg cm^–2^) and stored in an argon-filled glovebox. A typical loading of cathode active materials on the post-drying electrode is ~1.5 mAh cm^–2^. The graphite electrode was processed by a similar method, using deionized water as the solvent; it contained 93 wt.% graphite, 2 wt. % carbon black, and 5 wt.% LiPAA (from a 10 wt.% aqueous solution) binder. The resulting slurry was coated on Cu foil. The coating thickness of the slurry was controlled within the range of 150–200 μm. The graphite electrode was punched into discs with a diameter of 14 mm and a mass loading of ~4.8 mg cm^–2^. A typical loading of anode active materials was ~1.8 mAh cm^–2^. The N/P ratio of the assembled full coin cells was around 1.2.

For graphite||LCO pouch cells, the active material loading of the LCO cathode and graphite anode was 98.6% and 96.7%, respectively. The press density of the LCO cathode and graphite anode was 4.1 g cc^–1^ and 1.7 g cc^–1^, respectively. The N/P ratio was ~1.1. For graphite||NCM811 pouch cells, the active material loading of the NCM811 cathode and graphite anode was 95.5% and 94.8%, respectively. The press density of the NCM811 cathode and graphite anode was 3.4 g cc^–1^ and 1.5 g cc^–1^, respectively. The N/P ratio was ~1.0.

### Electrochemical measurements

The graphite||NCM811 and graphite||LCO coin cells were assembled with one piece of polyethylene separator (Celgard) in an argon-filled glove box. Each coin cell was injected with 150 μL electrolyte to make sure the separator and electrode were completely infiltrated. For the 1-Ah pouch cell, 2 g electrolyte was injected to pursue a high energy density. Galvanostatic charge–discharge cycling and rate performances of full cells were conducted on a Landt battery cycler (Wuhan LAND Electronics Co., Ltd.). All cells were activated at a rate of 0.1 C for 3 cycles and then operated in the corresponding potential range for the subsequent cycles (graphite||NCM811: 2.8–4.6 V; graphite||LCO: 3–4.55 V) if not specially indicated. For GITT tests, cells were cycled at a rate of C/3 with a 12-min pulse time and a 5-h rest time (after 100 cycles at 1 C charge and 2 C discharge rates). EIS measurements were carried out using an electrochemical workstation with an amplitude of 10 mV over a frequency range from 10 kHz to 0.01 Hz. The electrochemical floating tests were performed in coin cells with NCM811 and Li metal as the cathode and anode, respectively. The cells were charged to 5 V and then maintained at this voltage for 12 h with their current monitored. The electrochemical stability of the electrolytes was evaluated by a linear sweep voltammetry method at a scan rate of 1 mV s^−1^ using a Li||Al configuration. The Li^+^ transference number (*t*_+_) is calculated by Bruce-Vincent-Evans equation^55^ (1):

1 $${t}_{+}=\frac{{I}_{{{{{{\rm{S}}}}}}}\left(\Delta {{{{{\rm{V}}}}}}-{I}_{0}{R}_{0}\right)}{{I}_{0}\left(\Delta {{{{{\rm{V}}}}}}-{I}_{{{{{{\rm{S}}}}}}}{R}_{{{{{{\rm{S}}}}}}}\right)}$$

where *I*_s_ is the steady-state current, *I*_0_ is the initial current, ΔV is the applied potential, *R*_s_ and *R*_0_ are the electrode resistances after and before the polarization, respectively. The potentiostatic polarization experiment was done with a polarizing voltage of 10 mV for 1 h; the impedances before and after polarization were measured in the frequency range of 0.01–10,000 Hz. The above electrochemical tests were run at 25 °C.

### Characterization

^1^H and ^19^F NMR spectra for TTMS and TM solvents were tested by a Bruker spectrometer using CDCl_3_ as solvent (1:25 *v*/*v*). For sample post-analysis, including SEM, Cryo-TEM, and XPS measurements, cycled coin cells were disassembled inside an argon-filled glovebox to collect the cathodes and anodes. These electrodes were rinsed with DMC to remove any residual electrolyte and dried for further characterizations. SEM was performed with a Hitachi SU-70 microscope at 3 kV. Cryo-TEM was carried out in a JEOL NEOARM200 aberration-corrected microscope at 200 kV. The specimens were transferred into the TEM column using double-sealed containers, and liquid nitrogen was poured into the Cryo-TEM holder (Fischione 2550) to cool the specimens below 170 °C, at which temperature the specimens were observed. Electron signals from 68 to 280 mrad were collected for high-angle annular dark-field (HAADF) images. XPS measurements were conducted in a Thermo Scientific ESCALAB 250Xi scanning X-ray microprobe with a monochromatic Al Kα X-ray (1486.6 eV) source. The samples were etched by Ar^+^ ions (2 kV, 2 μA, 45° incident angle) during various sputtering times (0, 120, 300, and 600 s) before measurement. X-ray diffraction patterns were obtained on a Rigaku MiniFlex II X-ray diffraction (XRD) instrument (Cu Kα radiation, 30 kV, 15 mA, and scan rate 0.3° min^–1^). The volumes of the pouch cells were determined from the photographs shown in Supplementary Fig. 34 and used to calculate expansion rates (*ER*s) following Eq. (2):

2 $${ER}{{{{\rm{s}}}}}=\frac{{V}_{{{{{\rm{after cycling}}}}}}{-}{V}_{{{{{\rm{before cycling}}}}}}}{{V}_{{{{{\rm{before cycling}}}}}}}\times 100\,\%$$

Freezing points were determined by differential scanning calorimetry (DSC) from room temperature (20 °C) to –80 °C and at a scanning rate of 5 °C min^−1^. The transition-metal (Ni, Co, and Mn) dissolution of the fully charged NCM811 cathodes (disassembled from graphite | |NCM811 full cells after 100 cycles at 1 C charge and 2 C discharge rates) was determined by inductively coupled plasma mass spectrometry (ICP-MS, Agilent 7700). The ARC test was conducted in the heat-wait-search mode. The temperature was increased in steps of 5 °C followed by a waiting period of 40 min. The limit of detection for the self-heating rate was 0.02 °C min^−1^. Nail penetration test: a nail with a diameter of 3 mm was used to puncture the batteries (full-charged) at a rate of 8 cm s^−1^ and then held for 1 h or until the temperature had decreased to 50 °C. The flammability tests and the contact angle measurements were performed on the polyethylene separators (Celgard). The contact angle measurements were carried on JC2000, Shanghai Zhongchen Digital Technic Apparatus Co., Ltd.

### Theoretical calculations

#### DFT calculations

DFT calculations were performed using Gaussian 09 package^56^ and Vienna Ab-initio Simulation Package (VASP)^57^ in this work.

##### Reduction/oxidation potential calculations

All the molecules were first optimized using Gaussian 09 with the three-parameter empirical formulation B3LYP for the exchange-correlation density-functional energy in conjunction with the basis set of 6–311 + G(d,p)^58^ under the gas phase approximation. The oxidation potential (${E}_{{{\mbox{ox}}}}^{^\circ }$) and reduction potential (${E}_{{{\mbox{re}}}}^{^\circ }$) were calculated according to Eqs. (3) and (4) using a Minnesota M05-2X density functional accompanied by the SMD solvation model^59^, where different relative dielectric constants (*ε*) were used for different electrolytes. The relative dielectric constant of acetone (*ε* = 20.493) was used for EC and DMC because it is close to the value of the typical mixture of linear and cyclic carbonates^60^. The relative dielectric constant of the mixture of TTMS-TM (1:2 by vol.) calculated by the method reported in the literature^61^ was 8.1. It is close to the value of tetrahydrofuran (*ε* = 7.426). Therefore, the relative dielectric constant of tetrahydrofuran (*ε* = 7.426) was used for TTMS and TM. To compare the oxidation stability and reduction stability, the relative dielectric constant of tetrahydrofuran (*ε* = 7.426) was also used for ETS, LiFSI and LiOTf.

3 $${E}_{{{\mbox{ox}}}}^{^\circ }\left({{{\mbox{M}}}}\right)=\left[\Delta {G}_{{{\mbox{e}}}}+\Delta {G}_{{{\mbox{s}}}}^{^\circ }\left({{{\mbox{M}}}}^{+}\right)-\Delta {G}_{{{\mbox{s}}}}^{^\circ }\left({{{\mbox{M}}}}\right)\right]/F-1{{{\mbox{.}}}}4{({{{\mbox{V}}}})}$$

4 $${E}_{{{\mbox{re}}}}^{^\circ }\left ({{{\mbox{M}}}}\right)=-\left[\Delta {G}_{{{\mbox{e}}}}+\Delta {G}_{{{\mbox{s}}}}^{^\circ }\left({{{\mbox{M}}}}^{-}\right)-\Delta {G}_{{{\mbox{s}}}}^{^\circ }\left({{{\mbox{M}}}}\right)\right]/F-1{{{\mbox{.}}}}4{({{{\mbox{V}}}})}$$

where Δ*G*_e_ is the ionization free energy for Eq. (3) or electron affinity for Eq. (4) in the gas phase at room temperature (298.15 K); Δ${G}_{{{\mbox{s}}}}^{^\circ }$(M^+^), Δ${G}_{{{\mbox{s}}}}^{^\circ }$(M^–^) and Δ${G}_{{{\mbox{s}}}}^{^\circ }$(M) are the Gibbs free energies of solvation for the oxidized complex (M^+^), reduced complex (M^–^) and initial complex (M) calculated at the standard condition (298.15 K, 1 M), respectively; *F* is the Faraday constant^62^. In the oxidation and reduction potential calculations, the value of potentials was converted from the absolute potential scale to the Li^+^/Li scale by subtraction of 1.4 V^45^.

##### Reaction pathway calculations

The reaction pathways for the reduction of TTMS were calculated using Gaussian 09 at the B3LYP/6-311 + G(d,p) level. For the reaction pathway, the TTMS combines with one electron and Li^+^ and is decomposed to smaller molecules. Then the smaller molecules either polymerize or recombine with electrons and Li^+^ to be reduced. The associated reaction energy of each step was calculated.

##### Binding energy calculations

The binding energies (*G*_binding_) between Li^+^ and the solvents were calculated using Gaussian 09 at the B3LYP/6-311 + G(d,p) level. The binding energy values were obtained according to Eq. (5).

5 $${G}_{{{{{{\rm{binding}}}}}}}={G}_{{{{{{\rm{Solvent}}}}}}}+{G}_{{{{{{\rm{Li}}}}}}}-{G}_{{{{{{\rm{Li}}}}}}-{{{{{\rm{Solvent}}}}}}}$$

where *G*_Solvent_, *G*_Li_, and *G*_Li-Solvent_ are the Gibbs free energies of the free solvent, free Li^+^, and their complex, respectively.

##### H-transfer reaction energy calculations

The H-transfer reaction energies were calculated since many previous studies reported that H-transfer occurs during the initial oxidation process^63^. The DFT calculation as implemented in VASP was performed in this work with the projector augmented wave (PAW) method and Perdew-Burke-Ernzerhof (PBE) exchange-correlation functional in the Generalized Gradient Approximation (GGA). A 5 × 5 × 1 slab of NCM811 (001) with eight atomic layers and a vacuum layer of 20 Å along the *z* axis was constructed. Solvent molecules were positioned on the slab of NCM811 (001). The DFT + U was employed, where the one-site coulombic (*U*) and exchange (*J*) terms are combined into a single *U* parameter (*U*_eff_, *U*_eff_ = *U*-*J*). The *U*_eff_ was applied on the transition metal *d* states based on previous literature (*U*_eff_ = 6.4, 4.0 and 3.3 eV for Ni, Mn and Co, *J* = 0.0 eV)^64^. The spin-polarized calculations were considered. Only the Gamma point was used for sampling the Brillouin’s zone. The wave functions were expanded by using the plane-wave energy cutoff of 550 eV. Dipole corrections^65^ were included in the surface normal direction (IDIPOL=3, LDIPOL=TRUE). The DFT-D3 approach^66^ was included to describe the long-range van der Waals (vdW) interaction. The constructed structures were relaxed and the convergence criteria for electronic self-consistent iteration and ionic relaxation were set to 1 × 10^−5^ eV and 0.01 eV Å^−1^, respectively. Four bottom atomic layers of slab model were fixed while four surface atomic layers were allowed to relax.

### AIMD simulations

The AIMD simulations were employed using VASP. Exchange-correlation potentials were parameterized using the GGA employing the functional of PBE. The PAW approach was used to represent the core electrons and a kinetic energy cutoff of 550 eV was chosen to expand the mono-electronic states in plane waves.

#### Electronic structure of electrolytes

For each electrolyte system, the molecular model was constructed by placing the solvents, Li^+^, and FSI^−^ based on their molar ratio and density information. The size and the number of solvents/lithium salts in the AIMD simulation box of electrolytes were summarized in Supplementary Table 8. Because of the large size of the cells, only the Gamma point was used for sampling the Brillouin’s zone. All molecular dynamics simulations were performed in the NVT ensemble at 300 K using a Nosé−Hoover thermostat. The hydrogen masses were replaced by tritium masses to allow Born–Oppenheimer dynamics time steps of 1 fs^67^. A 15 ps of dynamic simulations were performed. For each electrolyte system, PDOS of the solvent and FSI^–^ were calculated. The center (*ξ*) of PDOS above 0 eV was calculated as follows:

6 $${{\xi }}=\frac{{\int} _{0}^{+{{\infty }}}E\times D\left(E\right){dE}}{{\int }_{0}^{+{{\infty }}}D\left(E\right){dE}}$$

where *D*(*E*) is the density of state of the solvent and FSI^–^ in the electrolyte, and *E* is the electron energy. We then defined a parameter of *RI* as follows:

7 $${RI}={{{\xi }}_{{{\mbox{Solvent}}}}{-}{\xi }}_{{{{\mbox{FSI}}}}^{{{{{{\rm{{-}}}}}}}}}$$

where *ξ*_Solvent_ and *ξ*_FSI_^–^ are the PDOS centers of the solvent and FSI^–^, respectively.

#### Reduction products calculations

The reduction products were obtained by the AIMD simulation of the electrolyte on the lithiated graphite anode^68^. The anode was comprised of three layers of fully discharged graphite (LiC_6_) with a vacuum layer of 20 Å along the *z* axis. Carbon edges were functionalized with all oxygens. Then, anode slab model was optimized by DFT calculations using VASP with a 2 × 2 × 1 Monkhorst-Pack *k*-point mesh. The 1.9 M LiFSI/TTMS-TM (3 LiFSI, 3 TTMS, and 6 TM molecules) electrolyte system was tested in this work. Initial geometries of the electrolyte were created based on their molar ratio and density information and then quenched using density functional forces. Finally, the anode and electrolyte models were combined for subsequent AIMD simulations. Because of the large size of the cells, only the Gamma point was used for sampling the Brillouin’s zone. AIMD simulations were then performed at an elevated temperature of 300 K for 5 ps using a Nosé−Hoover thermostat to allow fast equilibration. A final 10-ps dynamic simulation at 300 K was performed to check the reduction products.

#### Diffusion behavior calculations

We used the AIMD to simulate the lithium diffusion behaviors in the bulk LiF (${{\mbox{Fm}}}\overline{3}{{\mbox{m}}}$ space group), Li_2_O (${{\mbox{Fm}}}\overline{3}{{\mbox{m}}}$ space group) and Li_2_SO_3_ (${{\mbox{P}}}\overline{3}$ space group). LiF, Li_2_O, and Li_2_SO_3_ were optimized by DFT calculations using VASP in advance with the same convergence criteria and cut-off energy as H-transfer reaction energy calculations. The 4 × 4 × 4 Monkhorst-Pack *k*-point mesh was used for LiF and Li_2_O, and 4 × 4 × 3 Monkhorst-Pack *k*-point mesh was used for Li_2_SO_3_. Then, the optimized structures (LiF, Li_2_O, and Li_2_SO_3_) were used for subsequent AIMD simulations. The optimized structures were heated to an initial temperature of 300 K for 5 ps using a Nosé−Hoover thermostat. The structures were then heated to targeted temperature over a time period of 5 ps. The total time was set to 20 ps with a time step of 1 fs in the NVT ensemble. Using the Einstein relationship^69^, the Li^+^ diffusion coefficients at different temperatures can be calculated based on the slope of the average mean-square displacements (MSD) curves. The diffusion energy barriers and corresponding diffusion coefficients at 300 K were then calculated according to the Arrhenius equation^70^.

## Supplementary information

Supplementary Information

Description of Additional Supplementary Files

Supplementary Movie 1

Supplementary Movie 2

Supplementary Movie 3

Supplementary Movie 4

## Data Availability

The data that support the findings of this study are available from the corresponding author upon reasonable request.

## Code availability

The codes that support the findings of this study are available from the corresponding author upon reasonable request.
