# Supplementary material for: Multifunctional solvent molecule design enables high-voltage Li-ion batteries
Source: Nat Commun. 2023 Apr 18;14:2211. doi: 10.1038/s41467-023-37999-4 (PMC10113204; doi:10.1038/s41467-023-37999-4)
Supplement: Supplementary file 1 — Supplementary Information [file 41467_2023_37999_MOESM1_ESM.pdf]

# Supplementary information

# Multifunctional solvent molecule design enables high-voltage Li-ion batteries

Junbo Zhang[1,2#], Haikuo Zhang[1#], Suting Weng[3#], Ruhong Li[1], Di Lu[1], Tao Deng[4], Shuoqing Zhang[1], Ling Lv[1], Jiacheng Qi[1], Xuezhang Xiao[1], Liwu Fan[5], Shujiang Geng[2], Fuhui Wang[2], Lixin Chen[1,6], Malachi Noked[7]*, Xuefeng Wang[3,8]* and Xiulin Fan[1]*

*1 State Key Laboratory of Silicon Materials, School of Materials Science and Engineering, Zhejiang University, Hangzhou 310027, China.*

*2 Shenyang National Laboratory for Materials Science, Northeastern University, Shenyang, 110819, China.*

*3 Beijing National Laboratory for Condensed Matter Physics, Institute of Physics, Chinese Academy of Sciences, Beijing 100190, China.*

*4 Department of Chemical and Biomolecular Engineering, University of Maryland, College Park, MD, USA.*

*5 State Key Laboratory of Clean Energy Utilization, School of Energy Engineering, Zhejiang University, Hangzhou, 310027, China.*

*6 Key Laboratory of Advanced Materials and Applications for Batteries of Zhejiang Province, Hangzhou 310013, China.*

*7 Department of Chemistry, Bar-Ilan University, Ramat Gan, Israel.*

*8 Tianmu Lake Institute of Advanced Energy Storage Technologies Co. Ltd., Liyang, 213300, Jiangsu, China*

[#]These authors contributed equally to this work.

*Corresponding authors: malachi.noked@biu.ac.il, wxf@iphy.ac.cn, xlfan@zju.edu.cn

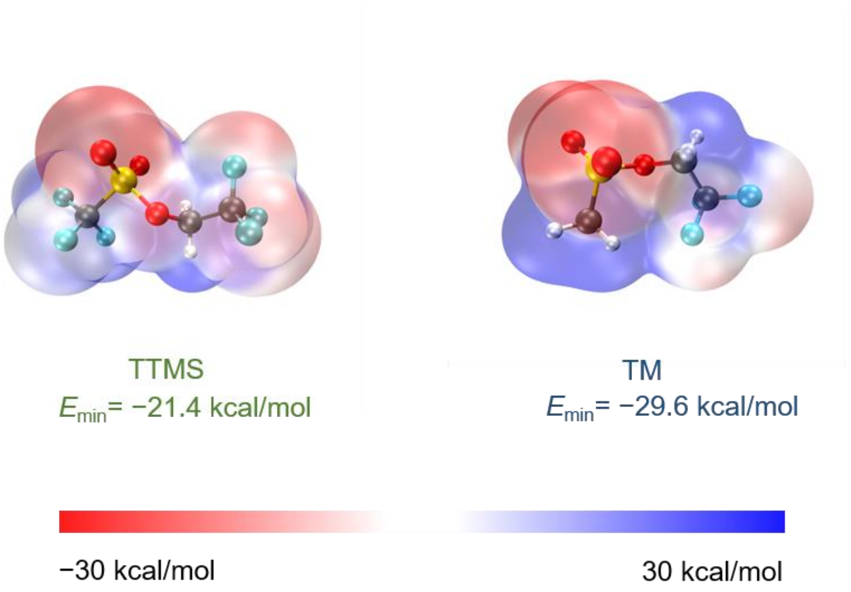


**Supplementary Fig. 1**. The electrostatic potential (ESP) mapped molecular van der Waals (vdW) surface of TTMS and TM solvents.

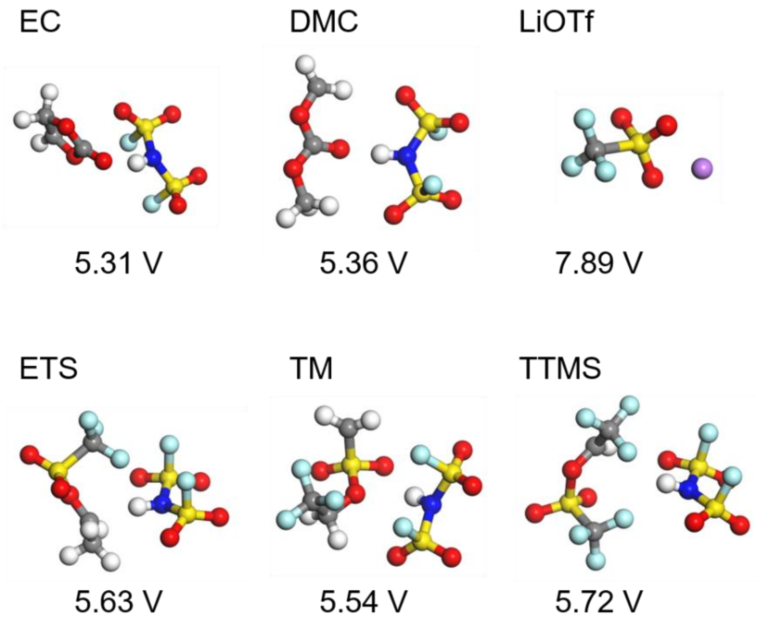


**Supplementary Fig. 2** Calculated oxidation potentials (V *vs*. $Li^+/Li$) of LiOTf and solvents with SMD solvation model at M05-2X/6-311+G(d,p) level.

The oxidation stability of solvents is highly affected by anion. The presence of anion around the solvent significantly induces H-transfer reaction, thus decreasing the complex oxidation stability compared to the intrinsic oxidation stability for the isolated solvent. Therefore, the solvent/$FSI^-$ complexes were constructed for oxidation potential calculations.

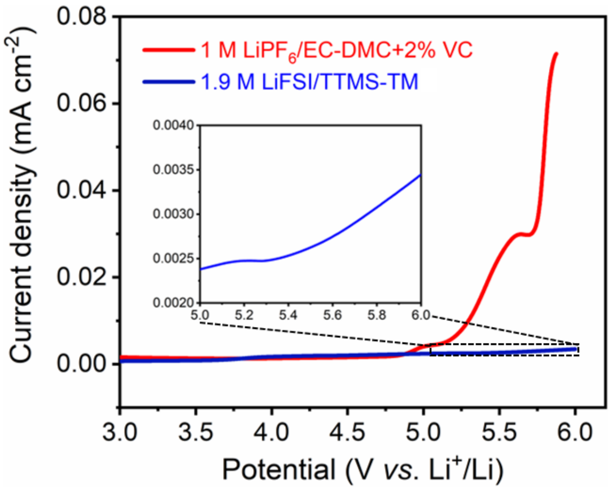


**Supplementary Fig. 3** Oxidation stability of 1 M $LiPF_6$/EC–DMC+2%VC electrolyte and 1.9 M LiFSI/TTMS–TM electrolyte at a scanning rate of 1 mV $s^{-1}$.

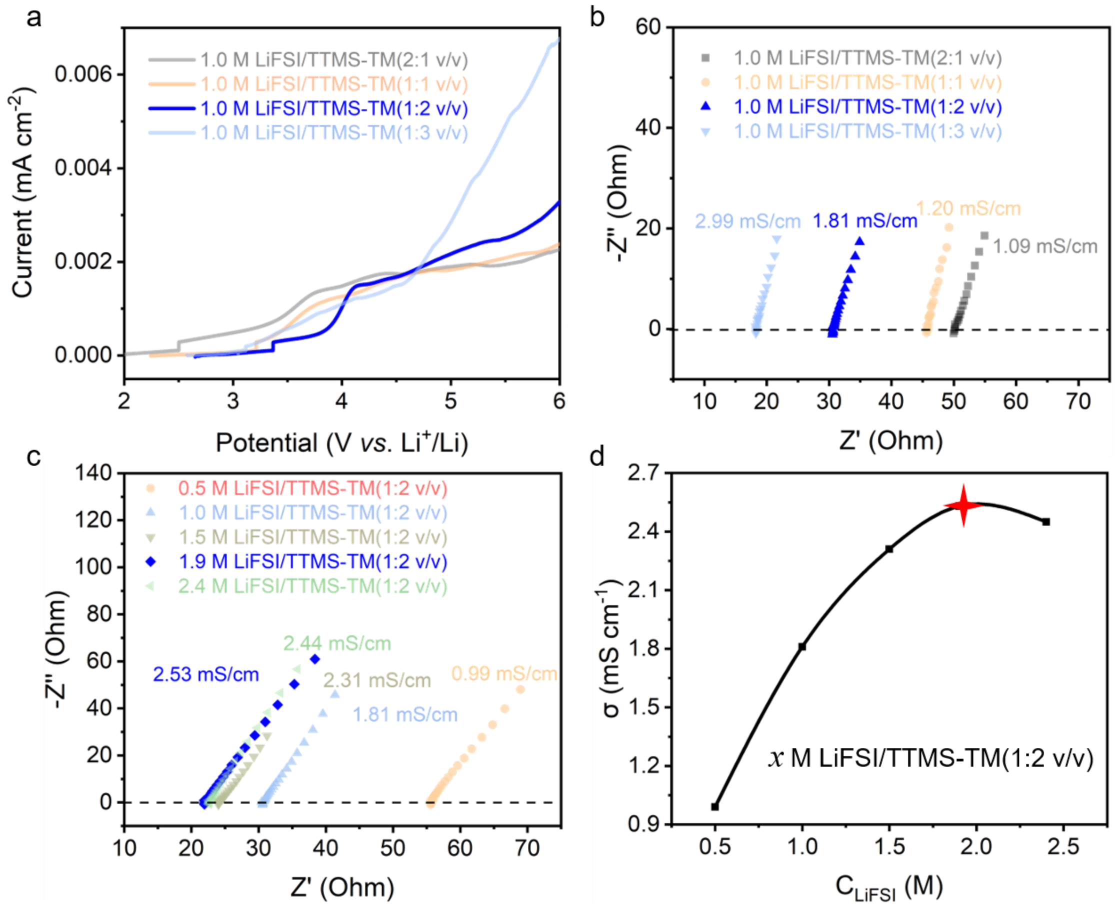


**Supplementary Fig. 4** Systematic screening of the electrolyte formulation with an optimum ratio. a, Oxidation stability of different electrolytes with a formulation of 1.0 M LiFSI/TTMS-TM (*x*:*y* *v*/*v*). b, The $Li^+$ conductivity of different electrolytes with a formulation of 1.0 M LiFSI/TTMS-TM (*x*:*y* *v*/*v*). c, The Nyquist plots of *x* M LiFSI/TTMS-TM (1:2 *v*/*v*) electrolytes. d, The $Li^+$ conductivity of *x* M LiFSI/TTMS-TM (1:2 *v*/*v*) electrolytes.

By comprehensively comparing the oxidation stability and $Li^+$ conductivity of different electrolytes with a formulation of 1.0 M LiFSI/TTMS-TM (*x*:*y* *v*/*v*) in (a) and (b), the electrolyte with the solvent ratio of TTMS-TM (1:2 *v*/*v*) stands out. By further comparing the $Li^+$ conductivity of *x* M LiFSI/TTMS-TM (1:2 *v*/*v*) electrolytes, the 1.9 M LiFSI/TTMS–TM (1:2 *v*/*v*) is chosen as the optimum electrolyte formulation.

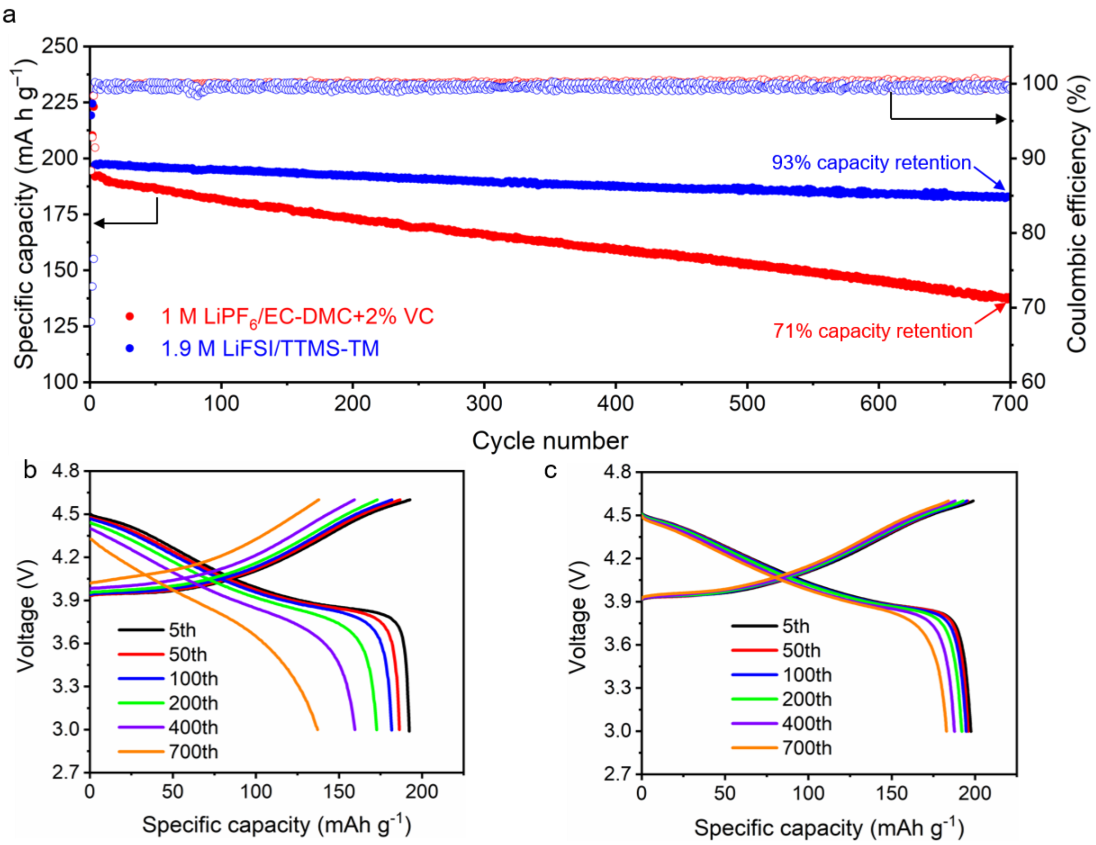


**Supplementary Fig. 5** Cycling performances (a) and corresponding voltage profiles (b and c) of Li||LCO cells using different electrolytes at 1 C charge and 2 C discharge rates in the voltage range from 3 to 4.6 V. The electrolytes used in (b) and (c) were 1 M $LiPF_6$/EC–DMC+2%VC electrolyte and 1.9 M LiFSI/TTMS–TM electrolyte, respectively.

The cycling stability of Li||LCO cells with the indicated electrolyte is slightly worse than graphite||LCO full cells, due to the electrolyte compatibility issue with Li metal anodes.

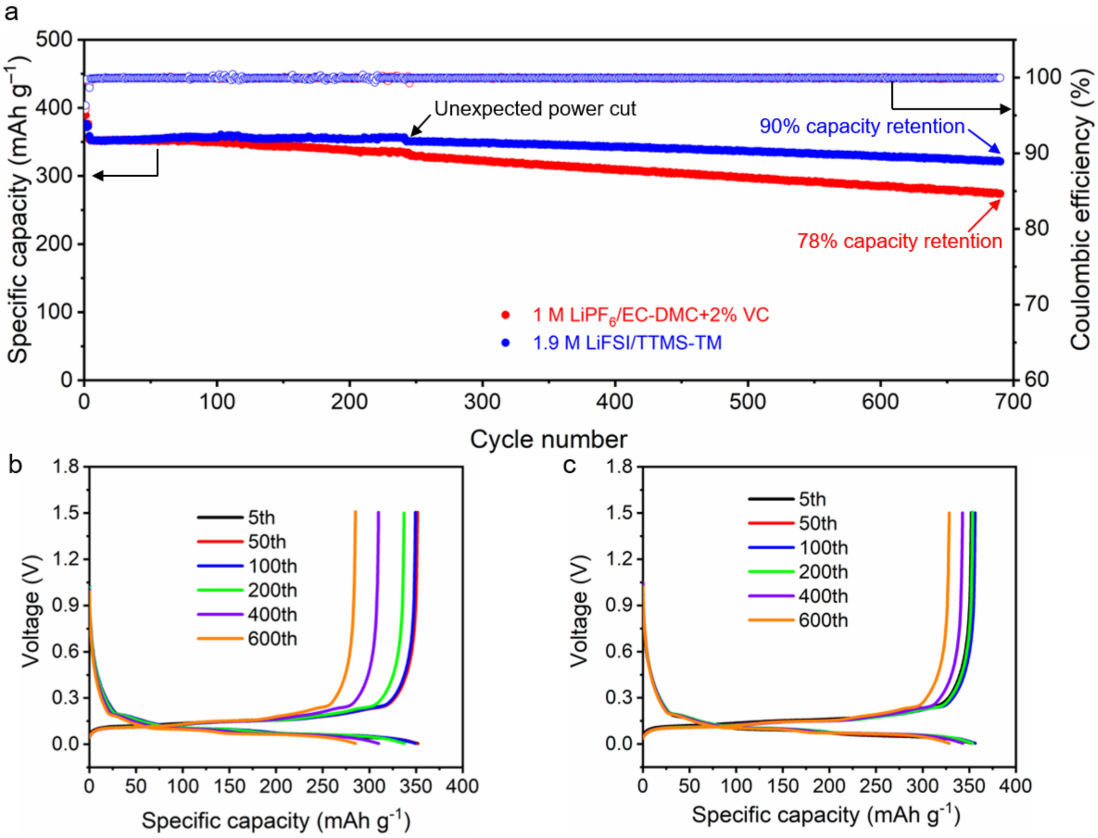


**Supplementary Fig. 6** Cycling performances (a) and corresponding voltage profiles (b and c) of Li||graphite cells using the indicated electrolytes at 0.5 C charge/discharge. The electrolytes used in (b) and (c) were 1 M $LiPF_6$/EC–DMC+2%VC electrolyte and 1.9 M LiFSI/TTMS–TM electrolyte, respectively.

The cycling stability of Li||graphite cells with the indicated electrolytes is slightly worse than graphite||LCO full cells, due to the electrolyte compatibility issue with Li metal anodes.

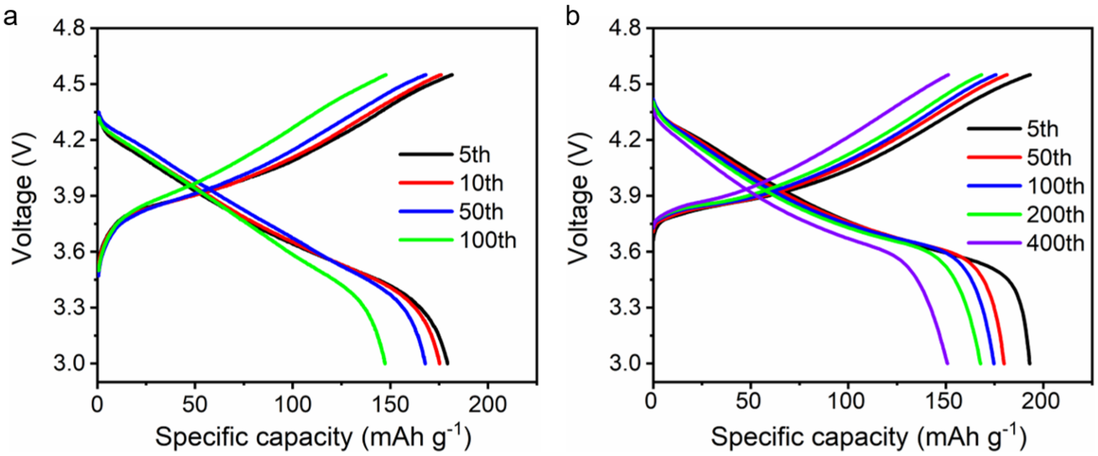


**Supplementary Fig. 7** Voltage profiles of graphite||LCO cells using 1 M $LiPF_6$/EC–DMC (a) and 1 M $LiPF_6$/EC–DMC+2%VC (b) electrolyte at 1 C charge and 2 C discharge rates in the voltage range from 3 to 4.55 V.

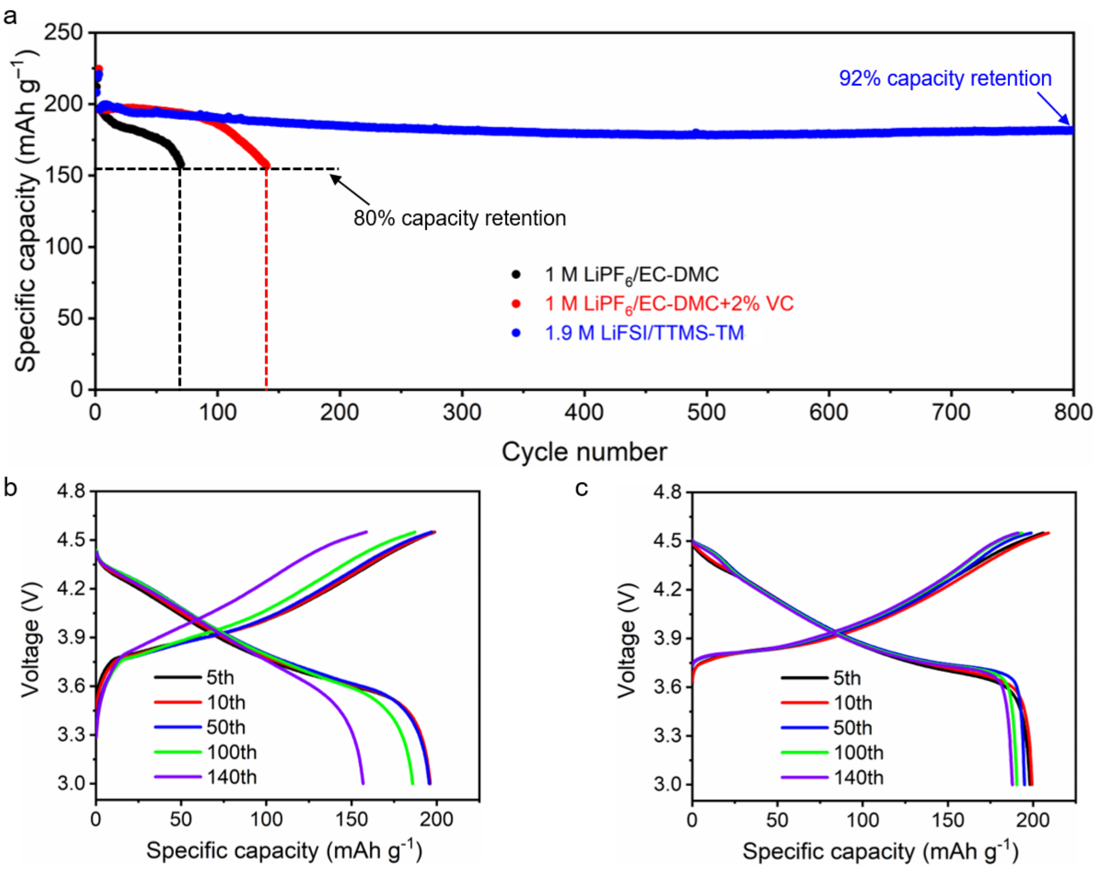


**Supplementary Fig. 8** Cycling performances (a) and corresponding voltage profiles (b and c) of graphite||LCO cells using the indicated electrolytes at 0.5 C charge and 1 C discharge rates in the voltage range from 3 to 4.55 V. The electrolytes used in (b) and (c) were 1 M $LiPF_6$/EC–DMC+2%VC electrolyte and 1.9 M LiFSI/TTMS–TM electrolyte, respectively.

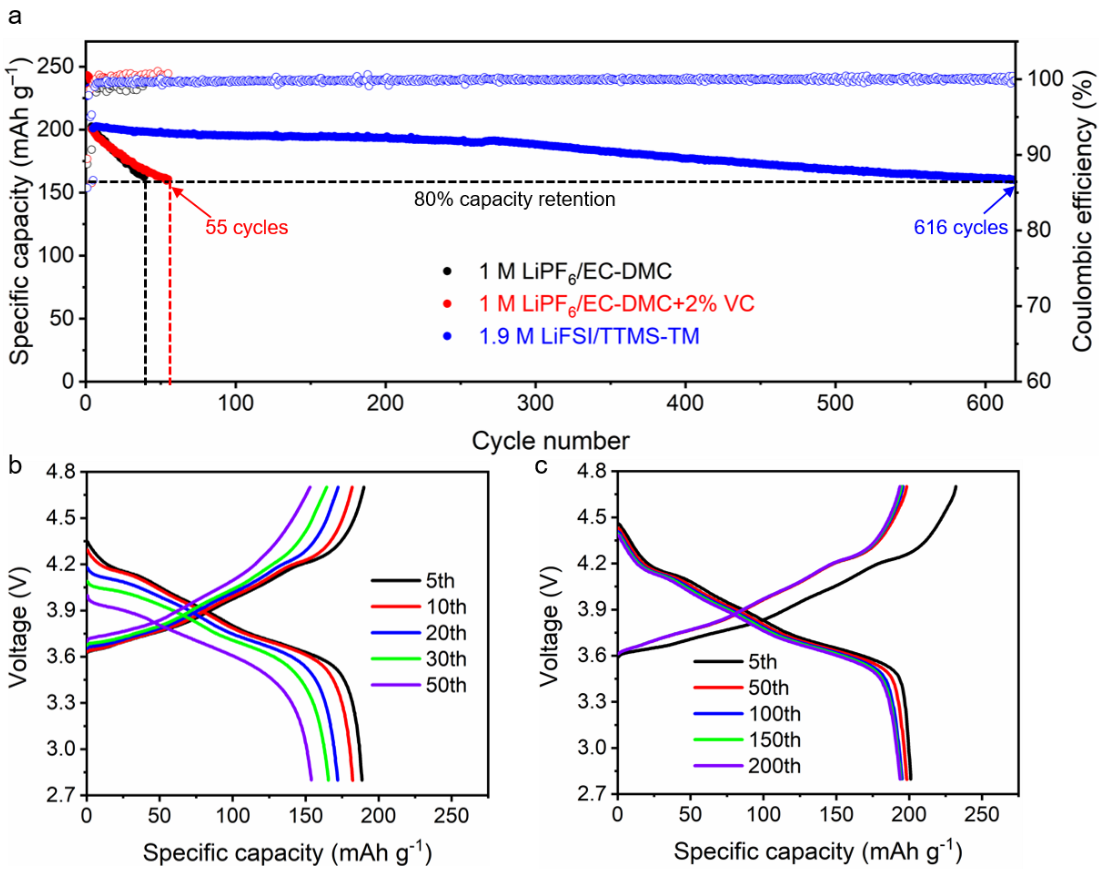


**Supplementary Fig. 9** Cycling performances (a) and corresponding voltage profiles (b and c) of Li||NCM811 batteries with the indicated electrolytes at 1 C charge and 2 C discharge rates in the voltage range from 2.8 to 4.7 V. The electrolytes used in (b) and (c) were 1 M $LiPF_6$/EC–DMC+2%VC electrolyte and 1.9 M LiFSI/TTMS–TM electrolyte, respectively.

The cycling stability of Li||NCM811 cells with the indicated electrolytes is slightly worse than graphite||NCM811 full cells, due to the electrolyte compatibility issue with Li metal anodes.

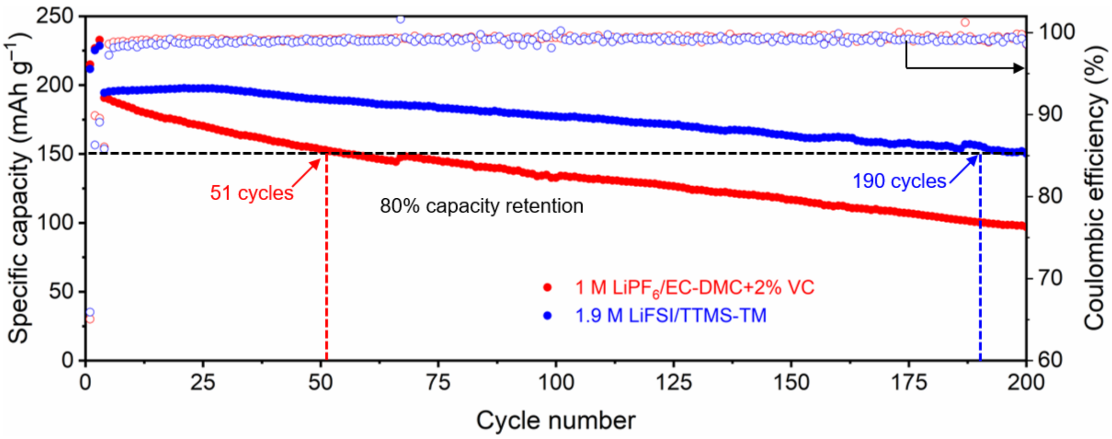


**Supplementary Fig. 10** High-temperature (60 °C) cycling performances of graphite||NCM811 cells using the indicated electrolytes at 1 C charge and 2 C discharge rates in the voltage range from 2.8 to 4.6 V.

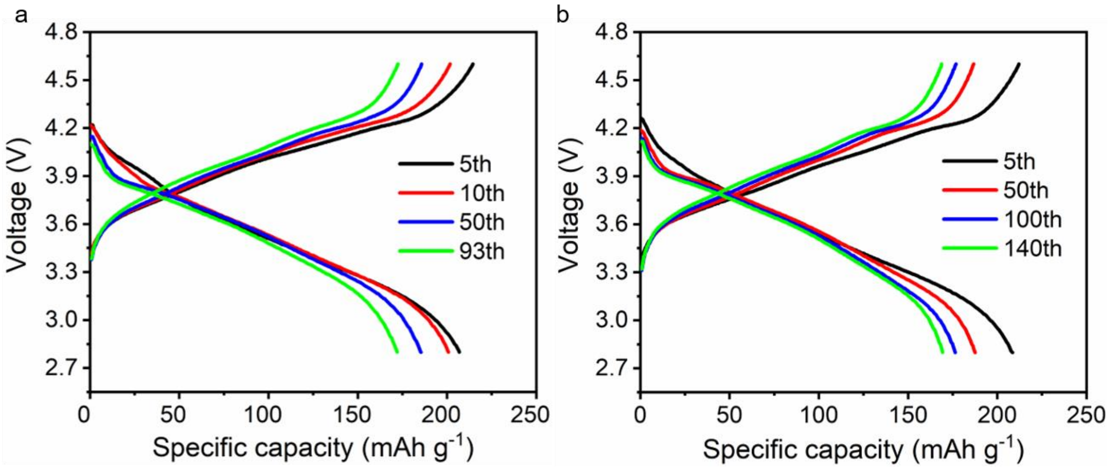


**Supplementary Fig. 11** Voltage profiles of the graphite||NCM811 cells using 1 M $LiPF_6$/EC–DMC (a) and 1 M $LiPF_6$/EC–DMC+2%VC (b) electrolyte at 1 C charge and 2 C discharge rates in the voltage range from 2.8 to 4.6 V.

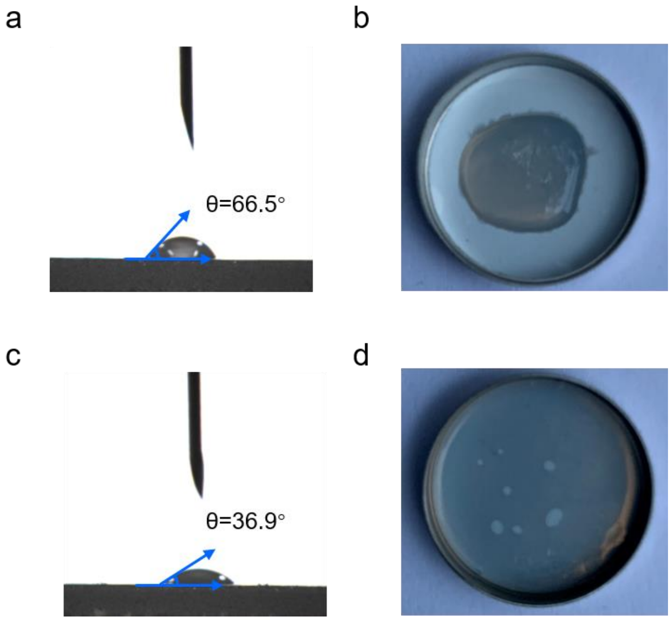


**Supplementary Fig. 12** Wettability test of 1 M $LiPF_6$/EC–DMC+2%VC electrolyte (a and b) and 1.9 M LiFSI/TTMS–TM electrolyte (c and d) with separators.

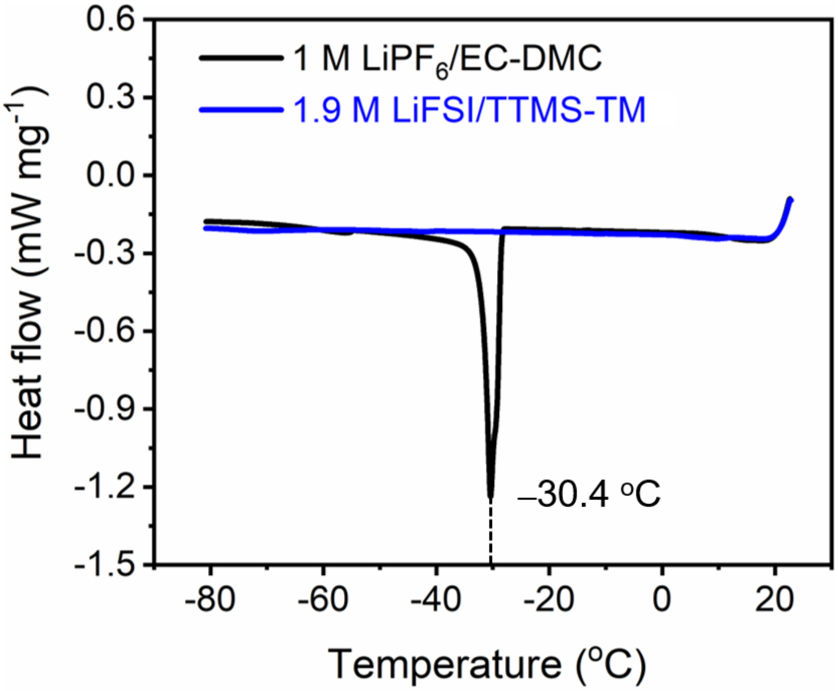


**Supplementary Fig. 13** Differential scanning calorimetry (DSC) cooling curves of different electrolytes. Electrolytes were cooled from room temperature (20 °C) to –80 °C at a rate of 5 °C/min.

The 1 M $LiPF_6$/EC–DMC electrolyte undergoes freeze under –30.4 °C as the exothermic peak appears. However, the 1.9 M LiFSI/TTMS–TM electrolyte remains liquid state even when the temperature cooled to –80 °C, suggesting the potential for low-temperature applications.

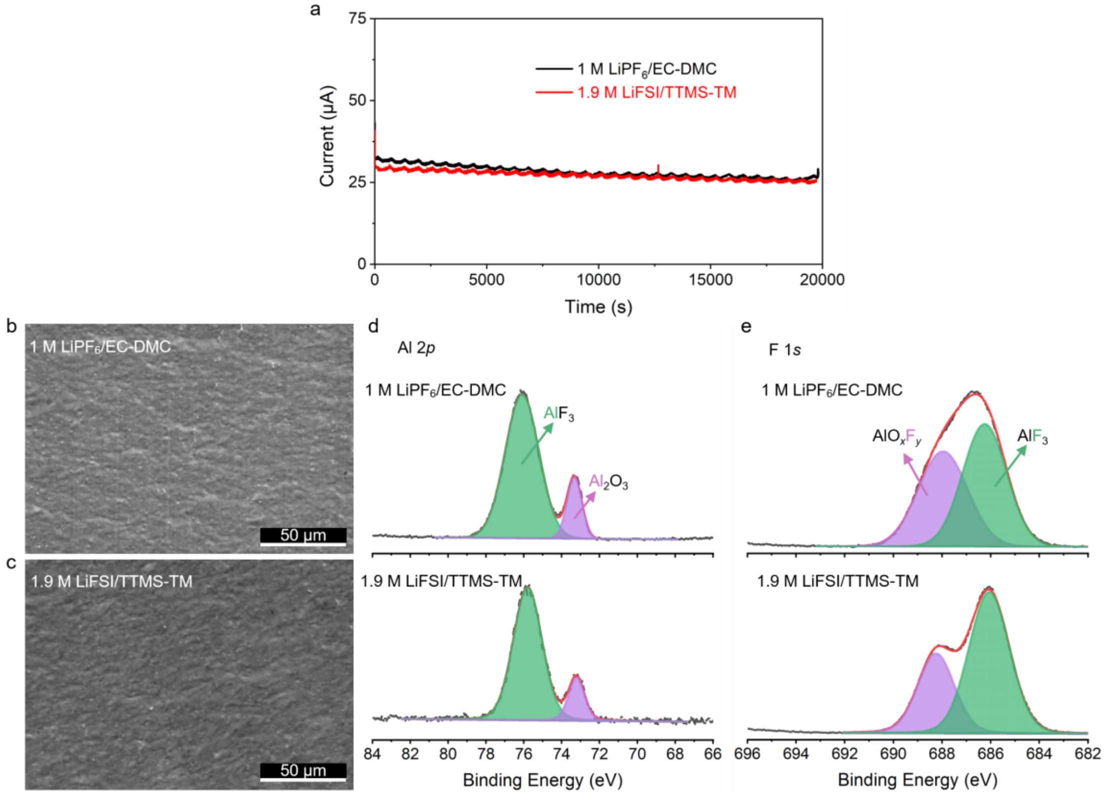


**Supplementary Fig. 14** Characterization of Al foils in Li||Al cells cycled with the 1.9 M LiFSI/TTMS-TM and 1 M $LiPF_6$/EC-DMC electrolyte. a, Leakage current as a functional of time during 4.7 V holding of Li||Al cells with different electrolytes. b,c, SEM images of the Al foils after 4.7 V holding experiments. d,e, Surface chemistry obtained by XPS measurement on Al foils after holding experiments: Al 2*p* (d) and F 1*s* (e) spectra.

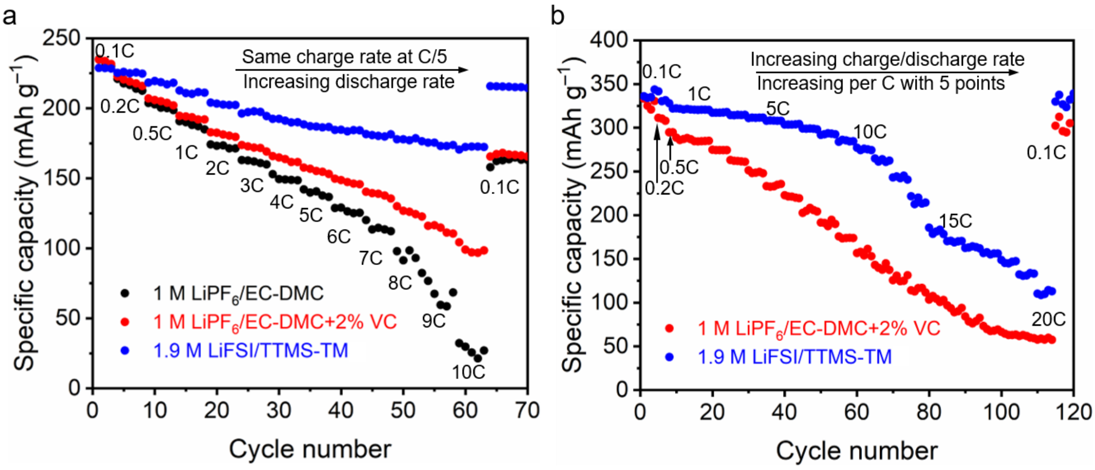


**Supplementary Fig. 15** Rate performances of half cells. (a) Rate performances of Li||NCM811 cells with varied discharge rates (*x* C) and same charge rate at C/5 after 3 activation cycles at C/10 using the investigated electrolytes. (b) Rate performances of Li||graphite cells with the increasing charging/discharging rates after 3 activation cycles at C/10 using the investigated electrolytes.

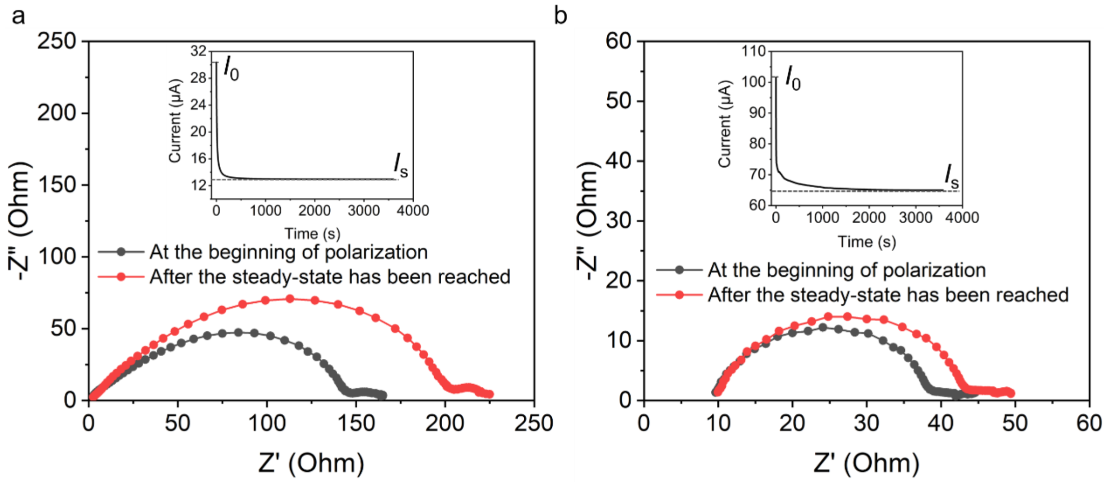


**Supplementary Fig. 16** Impedance spectra of the cell in the initial state and the steady-state with 1 M $LiPF_6$/EC-DMC (a) and 1.9 M LiFSI/TTMS-TM (b) electrolyte. Inset shows the polarization curves.

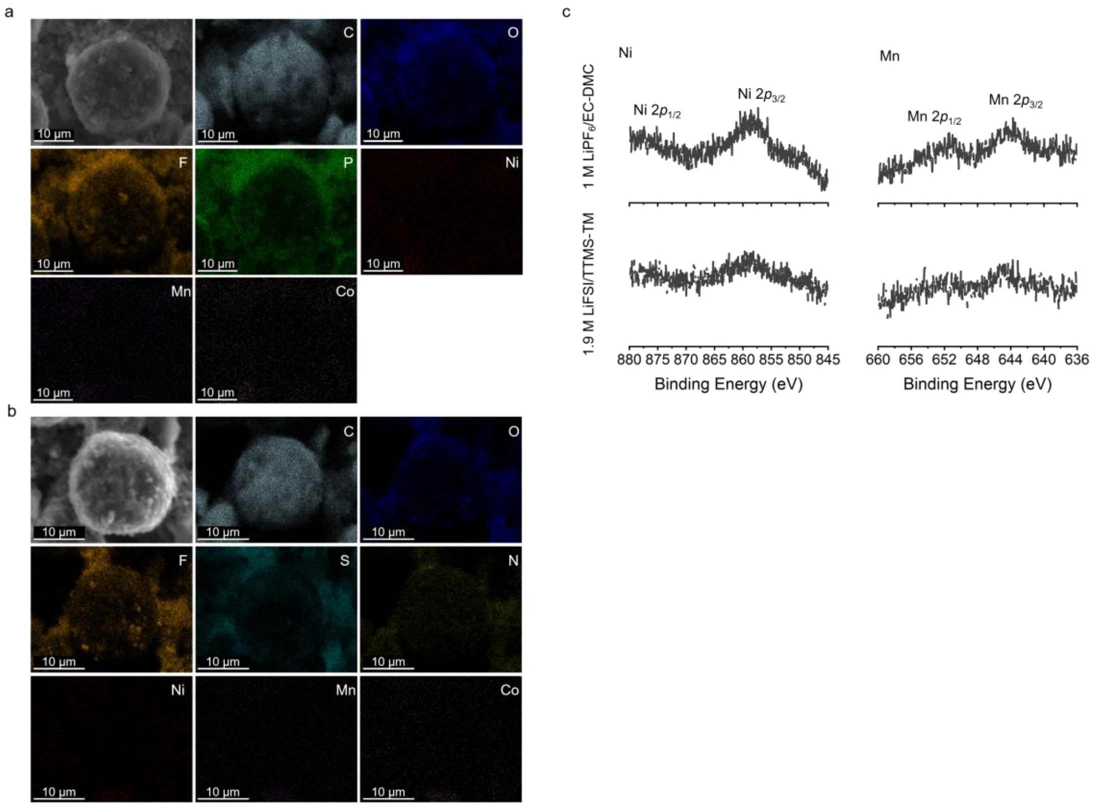


**Supplementary Fig. 17** Element contents on the graphite anodes retrieved from graphite||NCM811 cells after 100 cycles in different electrolytes analyzed by energy dispersive X-ray spectroscopy (a and b) and XPS (c) results.

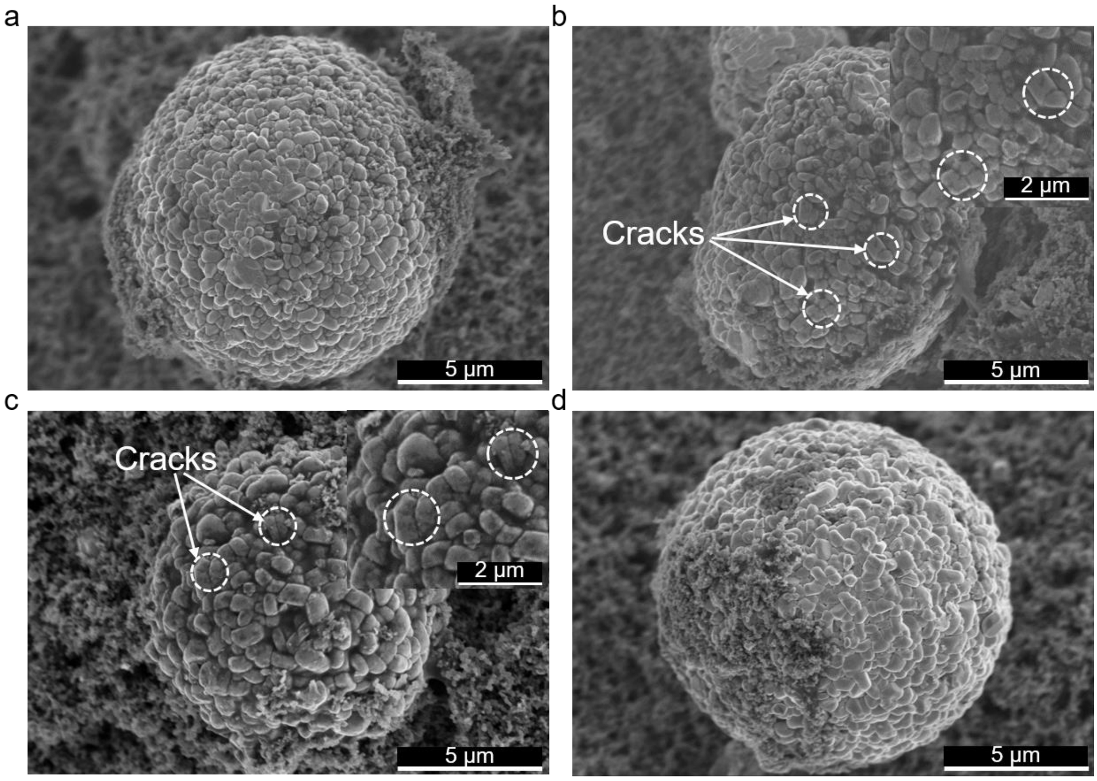


**Supplementary Fig. 18** SEM images of NCM811 cathodes. a, Pristine NCM811 cathode. b-d, NCM811 cathodes after 100 cycles in 1 M $LiPF_6$/EC–DMC electrolyte (b), 1 M $LiPF_6$/EC–DMC+2%VC electrolyte (c) and 1.9 M LiFSI/TTMS–TM electrolyte (d).

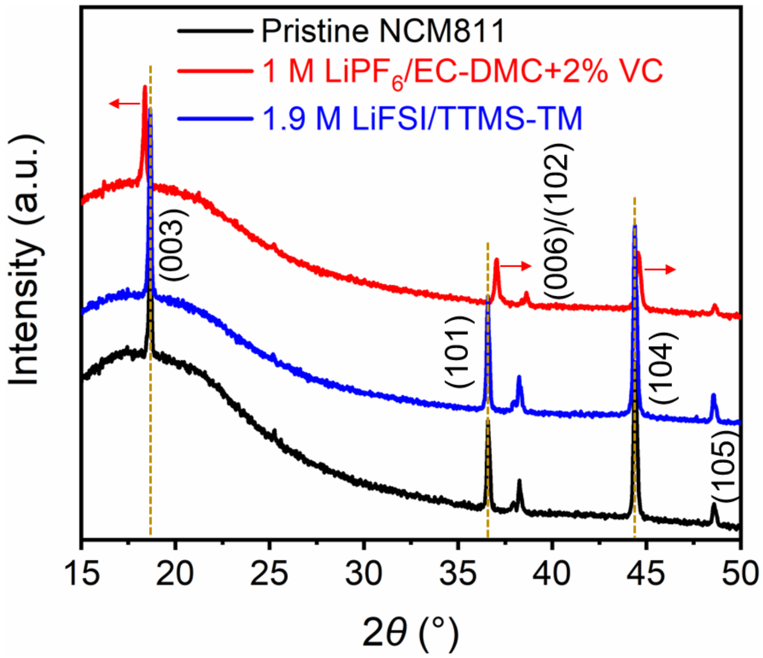


**Supplementary Fig. 19** X-ray diffraction patterns of the pristine NCM811 cathode and NCM811 cathodes after 100 cycles in investigated electrolytes.

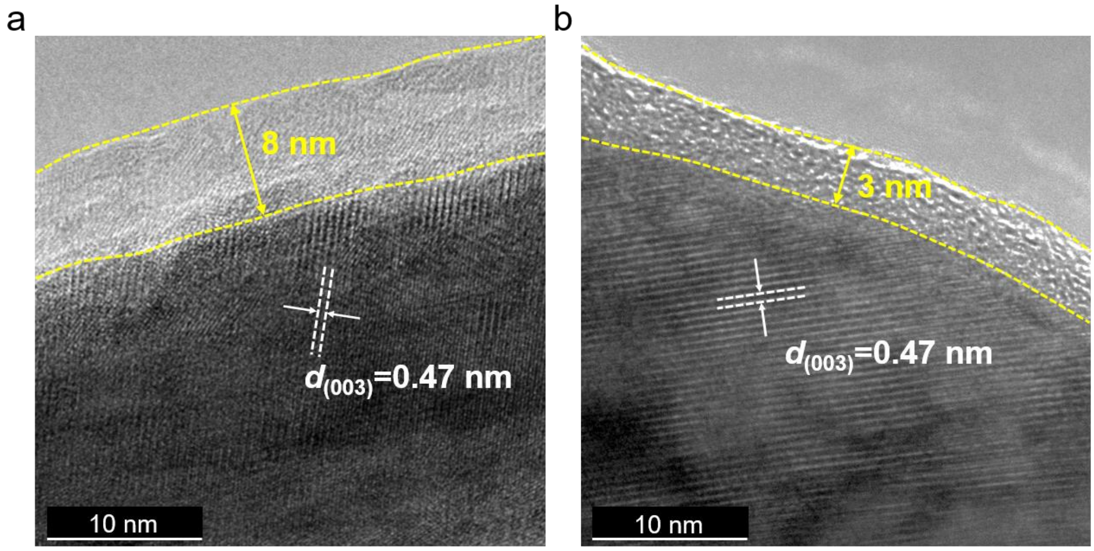


**Supplementary Fig. 20** High-resolution TEM images of LCO retrieved from the graphite||LCO cells after 100 cycles in 1 M $LiPF_6$/EC–DMC+2%VC electrolyte (a) and 1.9 M LiFSI/TTMS–TM electrolyte (b).

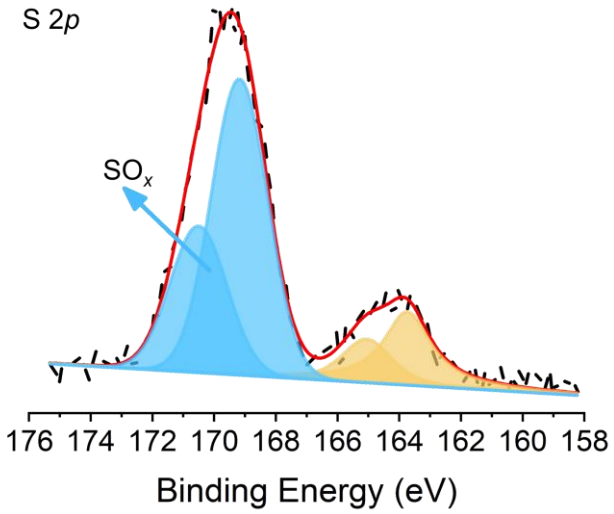


**Supplementary Fig. 21** XPS profiles of S 2*p* for NMC811 cathodes retrieved from the graphite||NCM811 cells after 100 cycles in the 1.9 M LiFSI/TTMS–TM electrolyte.

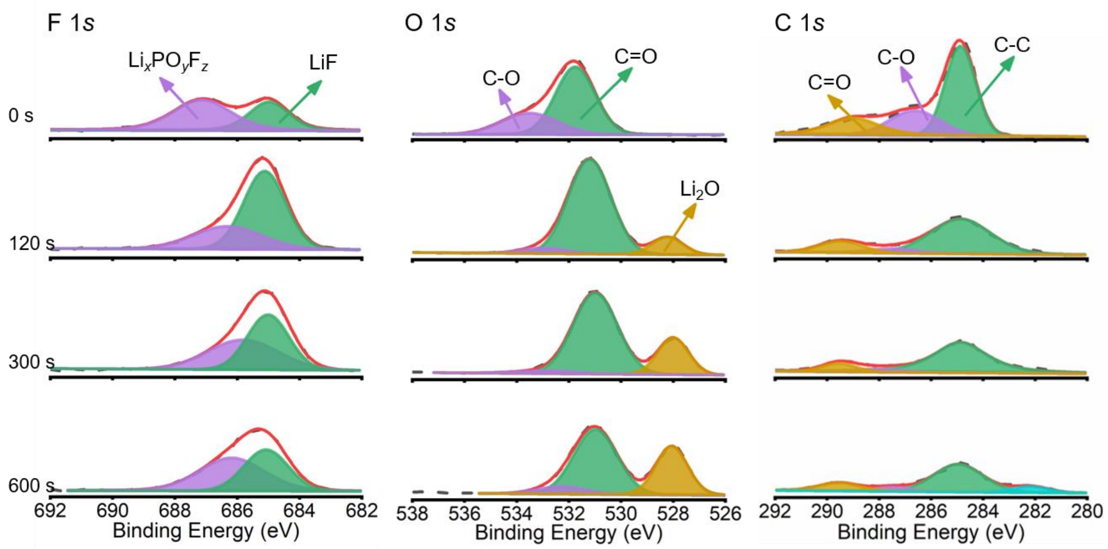


**Supplementary Fig. 22** SEI information obtained by XPS depth profiles of graphite retrieved from full cells after 100 cycles in 1 M $LiPF_6$/EC–DMC+2%VC electrolyte. Ar Sputtering (0 s, 120 s, 300 s and 600 s) was conducted to get the information in different depths.

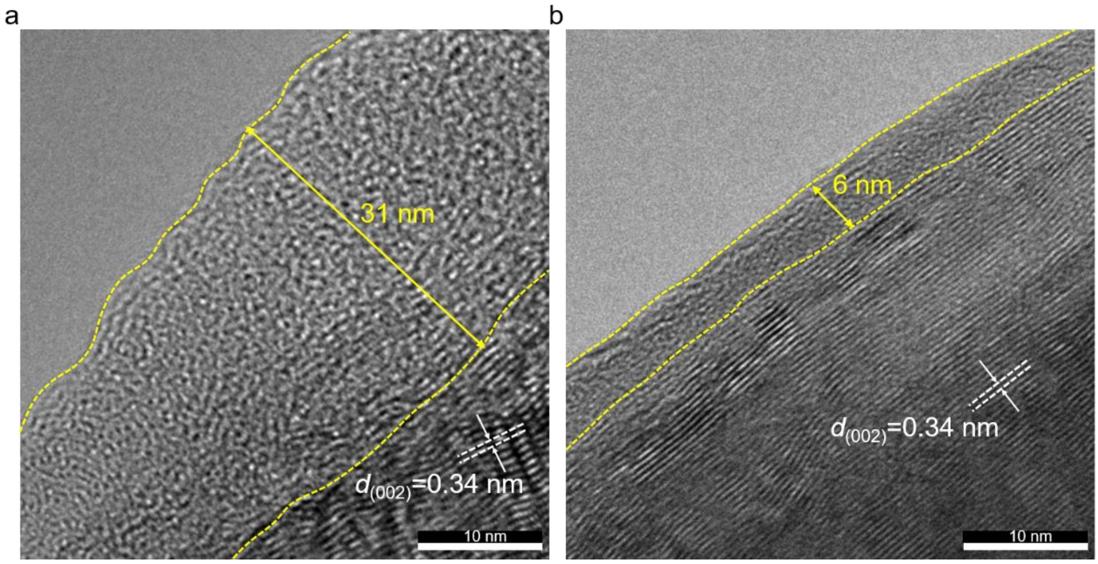


**Supplementary Fig. 23** Cryo-TEM images of graphite anodes retrieved from the graphite||NCM811 cells after 100 cycles in 1 M $LiPF_6$/EC–DMC+2%VC electrolyte (a) and 1.9 M LiFSI/TTMS–TM electrolyte (b).

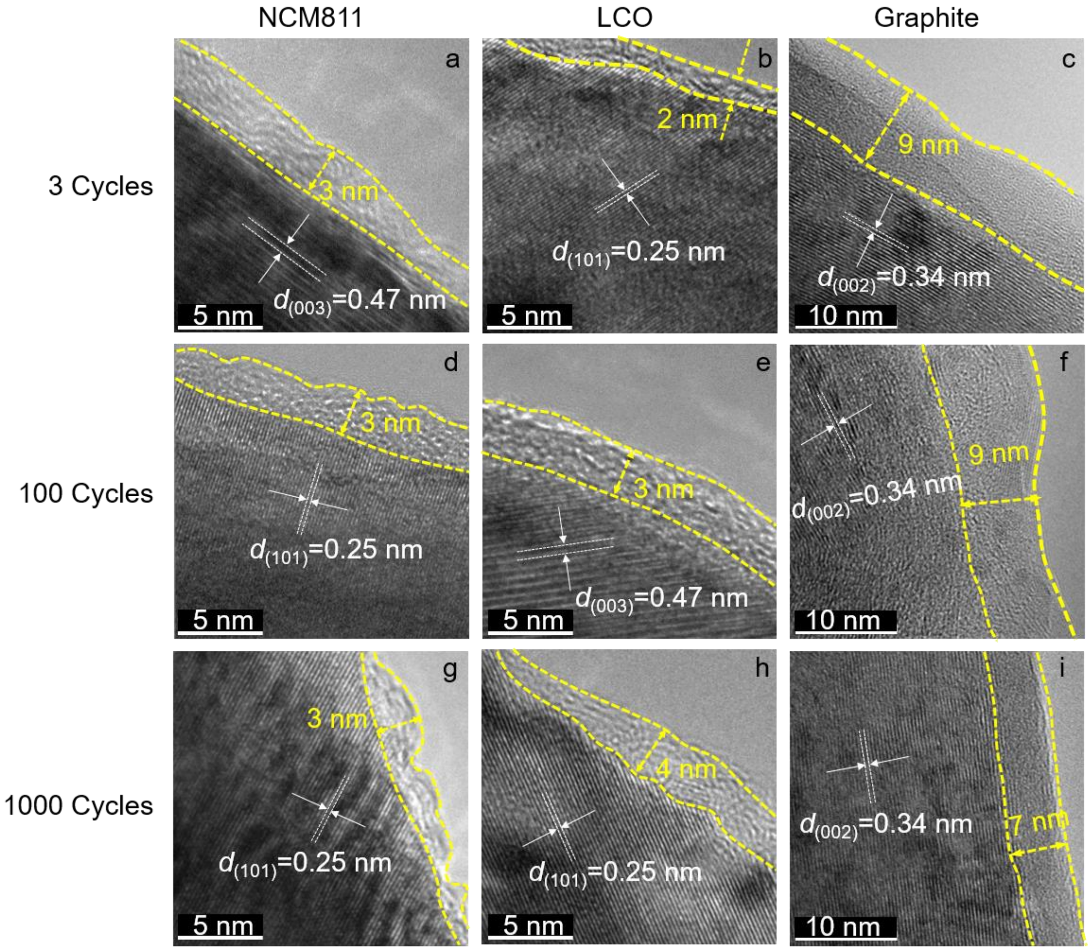


**Supplementary Fig. 24** TEM images of NCM811 (a, d and g), LCO (b, e and h), and graphite anodes (c, f and i) retrieved from full cells after 3 cycles, 100 cycles, and 1000 cycles in the 1.9 M LiFSI/TTMS–TM electrolyte, respectively.

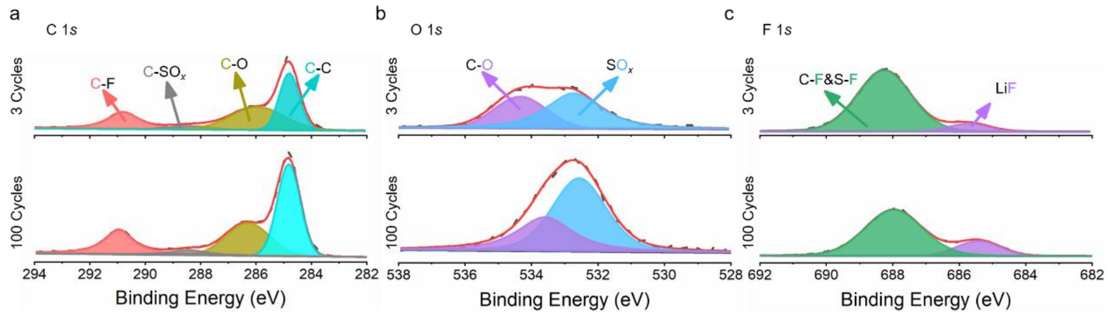


**Supplementary Fig. 25** XPS profiles of C 1*s* (a), O 1*s* (b) and F 1*s* (c) for the NCM811 cathode retrieved from full cells after 3 cycles and 100 cycles in the 1.9 M LiFSI/TTMS–TM electrolyte, respectively.

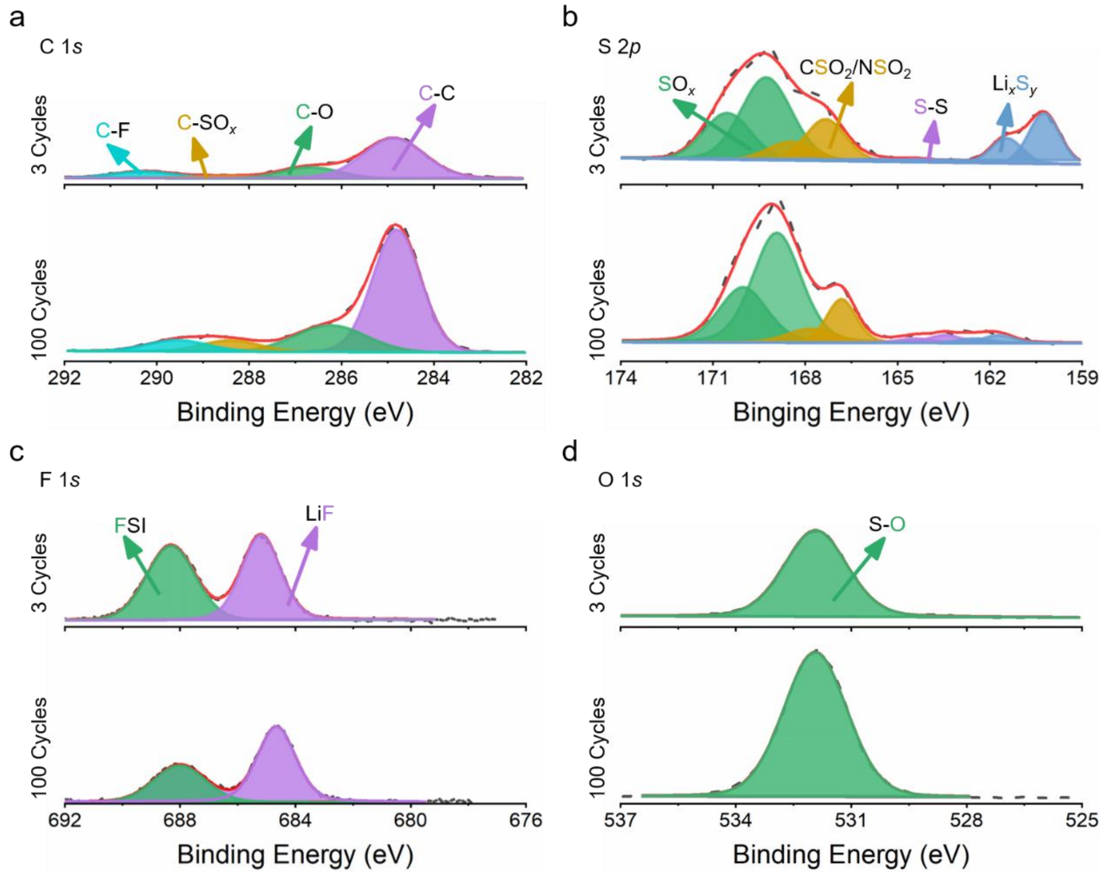


**Supplementary Fig. 26** XPS profiles of C 1*s* (a), S 2*p* (b), F 1*s* (c) and O 1*s* (d) for the graphite anode retrieved from full cells after 3 cycles and 100 cycles in the 1.9 M LiFSI/TTMS–TM electrolyte, respectively.

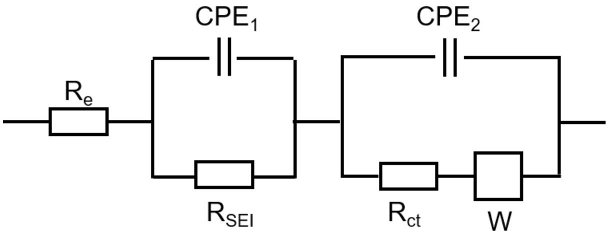


**Supplementary Fig. 27** The equivalent circuit used to fit the electrochemical impedance data.

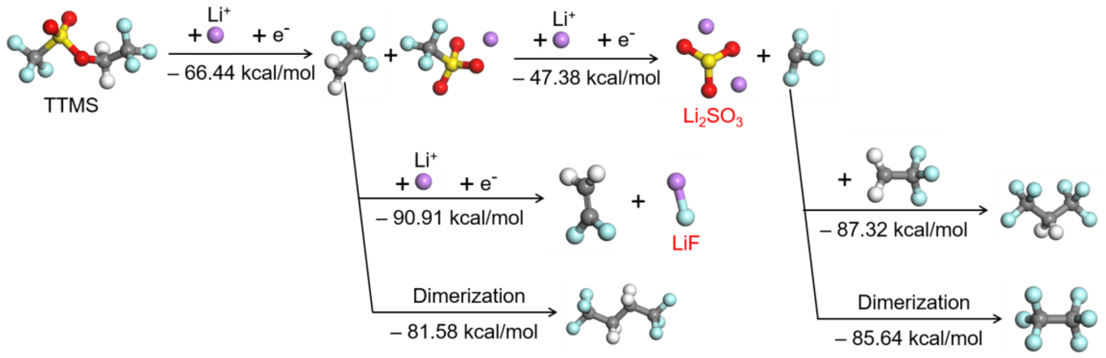


**Supplementary Fig. 28** Possible reduction pathways and associated reaction energies of the TTMS in 1.9 M LiFSI/TTMS-TM electrolyte.

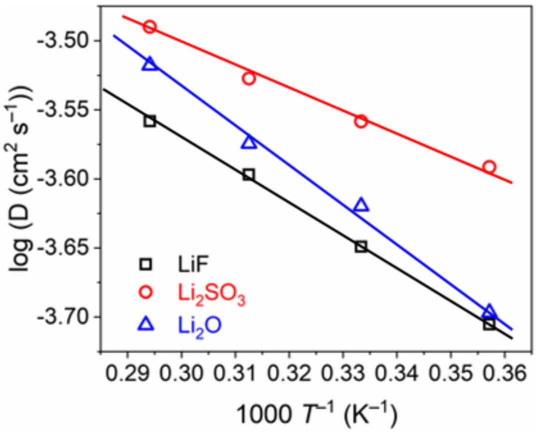


**Supplementary Fig. 29** Arrhenius plots of $Li^+$ diffusion coefficients in the bulk $Li_2SO_3$, LiF and $Li_2O$.

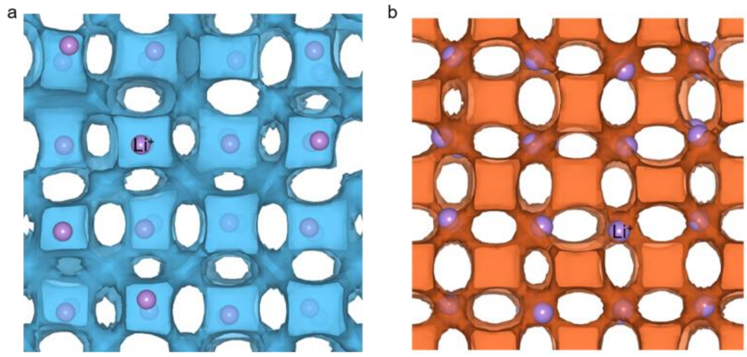


**Supplementary Fig. 30** Simulated possible $Li^+$ diffusion pathways in bulk LiF (a) and $Li_2O$ (b).

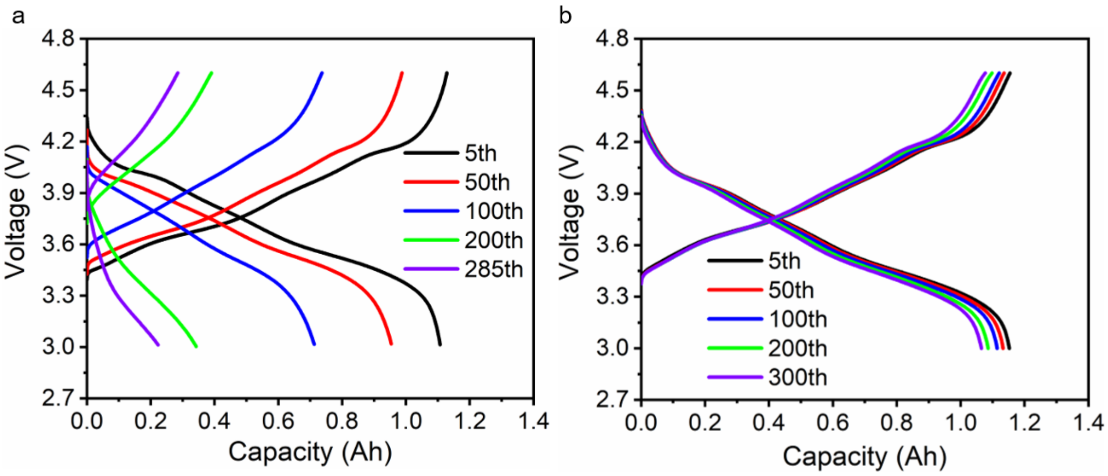


**Supplementary Fig. 31** Charge–discharge curves of graphite||NCM811 pouch cells using 1 M $LiPF_6$/EC–DMC+2%VC (a) and 1.9 M LiFSI/TTMS–TM (b) electrolytes at 0.5 C charge and 1 C discharge in the voltage range from 3 to 4.6 V.

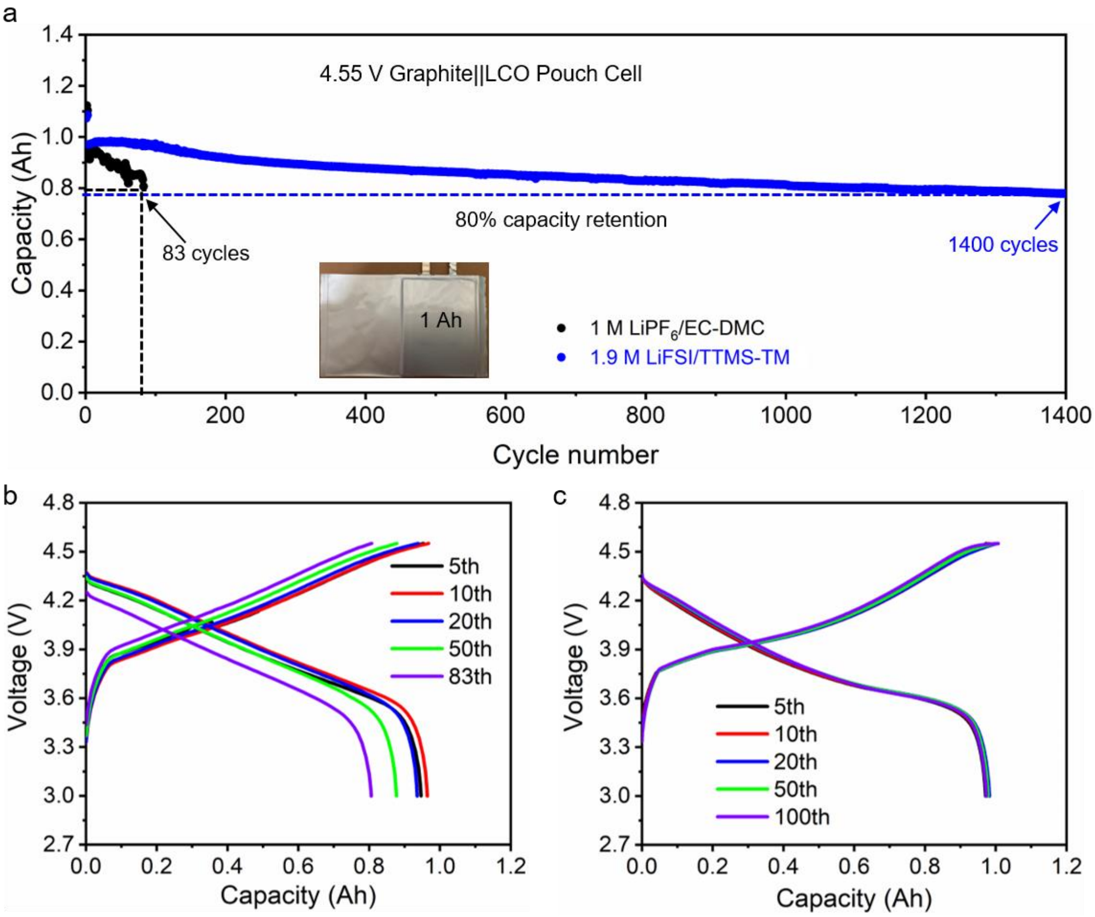


**Supplementary Fig. 32** Cycling performances (a) and corresponding voltage profiles (b and c) of graphite||LCO pouch cells using investigated electrolytes at 0.5 C charge/discharge in the voltage range from 3 to 4.55 V. The electrolytes used in (b) and (c) were 1 M $LiPF_6$/EC–DMC electrolyte and 1.9 M LiFSI/TTMS–TM electrolyte, respectively.

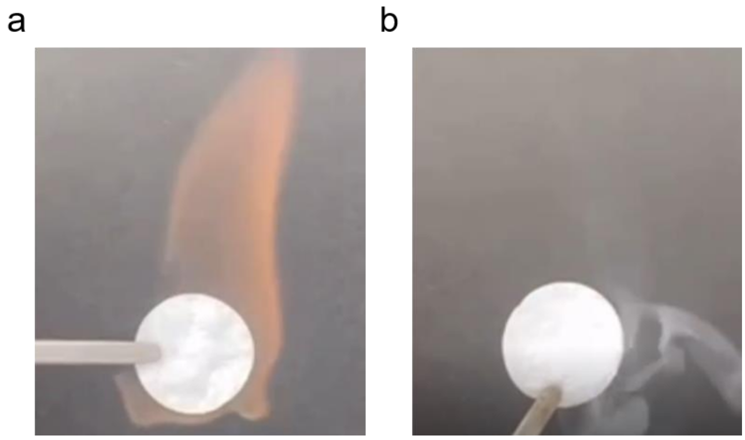


**Supplementary Fig. 33** Flammability of 1 M $LiPF_6$/EC–DMC electrolyte (a) and 1.9 M LiFSI/TTMS–TM electrolyte (b).

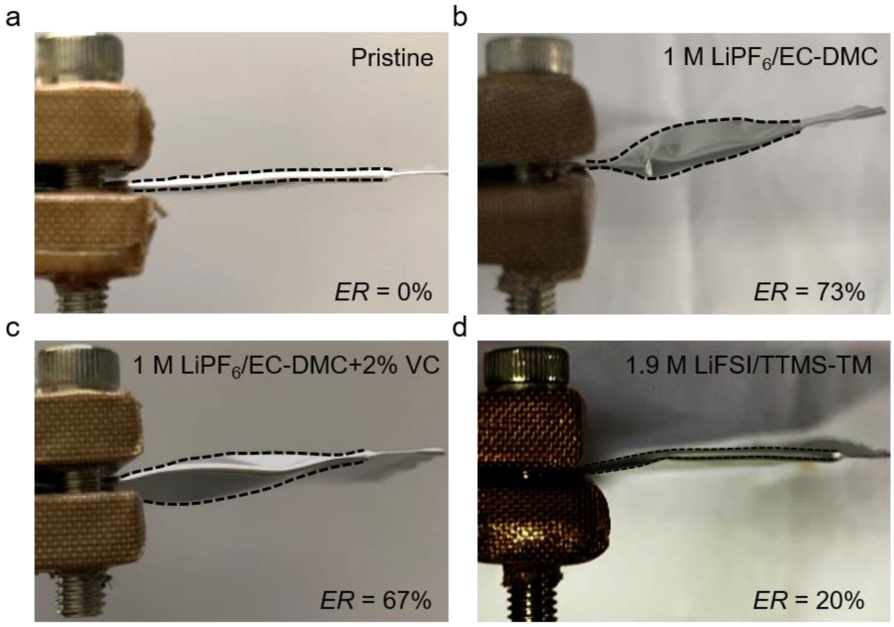


**Supplementary Fig. 34** Optical images of gas expansion in graphite||NCM811 pouch cells. a, Pristine graphite||NCM811 pouch cell. b-d, graphite||NCM811 pouch cells after 300 cycles in 1 M $LiPF_6$/EC–DMC electrolyte (b), 300 cycles in 1 M $LiPF_6$/EC–DMC+2%VC electrolyte (c) and 1000 cycles in 1.9 M LiFSI/TTMS–TM electrolyte (d).

The calculated gas expansion rates (*ER*s) of graphite||NCM811 pouch cells after 300 cycles in 1 M $LiPF_6$/EC–DMC electrolyte and 1 M $LiPF_6$/EC–DMC+2%VC electrolyte are 73% and 69%, respectively. In contrast, the *ER* of graphite||NCM811 pouch cell after 1000 cycles in the 1.9 M LiFSI/TTMS–TM electrolyte is about 20%, indicating a better stability between electrolyte and electrodes.

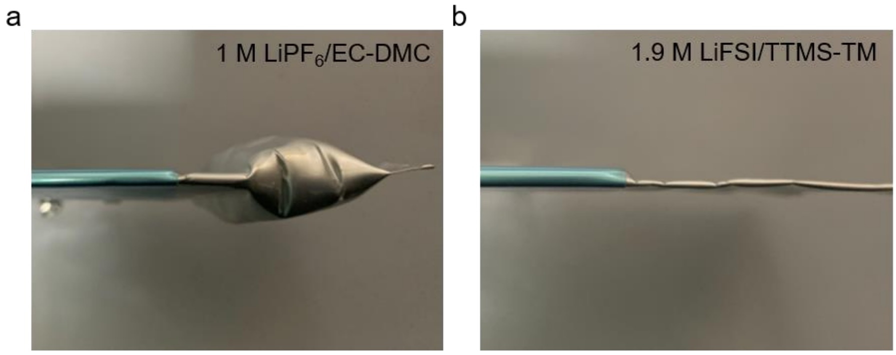


**Supplementary Fig. 35** Optical images of gas expansion in graphite||LCO pouch cells after 200 cycles in 1 M $LiPF_6$/EC–DMC electrolyte (a) and 1000 cycles in 1.9 M LiFSI/TTMS–TM electrolyte (b).

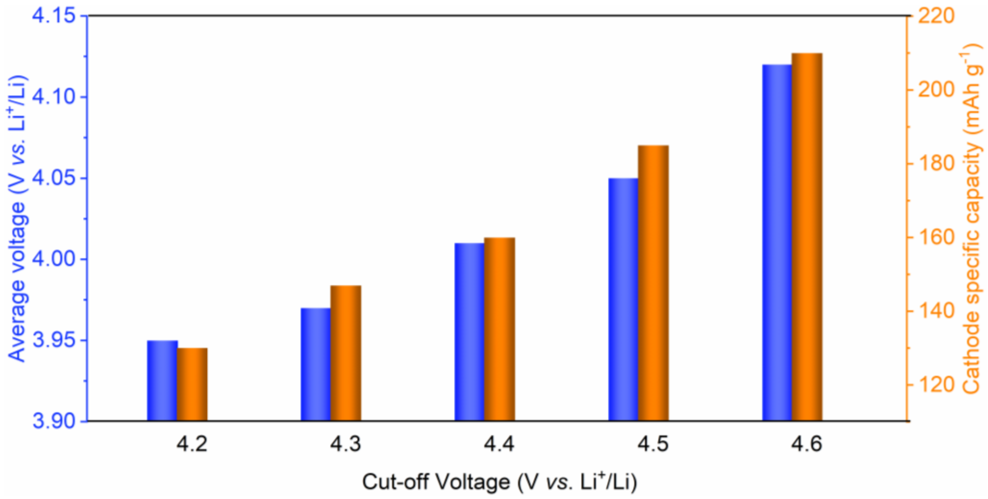


**Supplementary Fig. 36** Average voltage, cathode specific capacity, and gravimetric energy density of graphite||LCO pouch cells at different charging cut-off voltages.

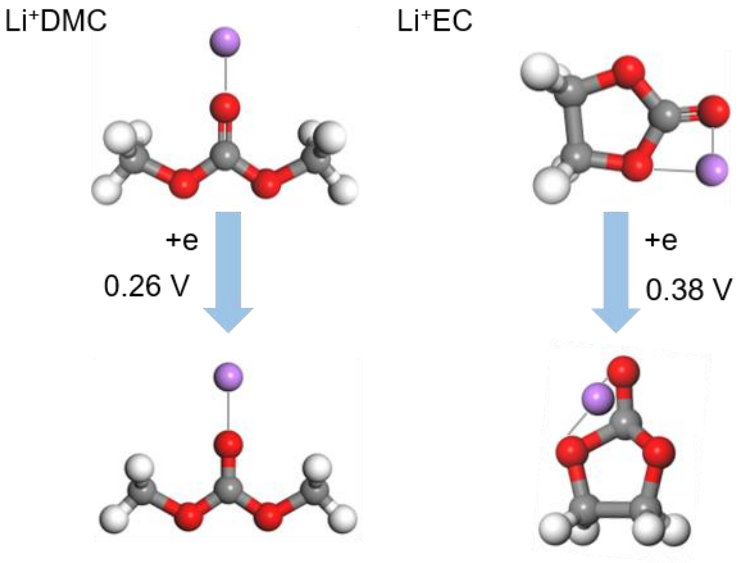


**Supplementary Fig. 37** Calculated reduction potentials (*vs*. $Li^+$/Li) of EC and DMC molecules with SMD solvation model at M05-2X/6-311+G(d,p) level.

The reduction stability of solvents is highly affected by surrounding $Li^+$ in the electrolyte. The solvents are polarized by surrounding $Li^+$ in the electrolyte, thus decreasing the complex reduction stability compared to the intrinsic reduction stability for the isolated solvent. Therefore, the solvent/$Li^+$ complexes were constructed for reduction potential calculations.

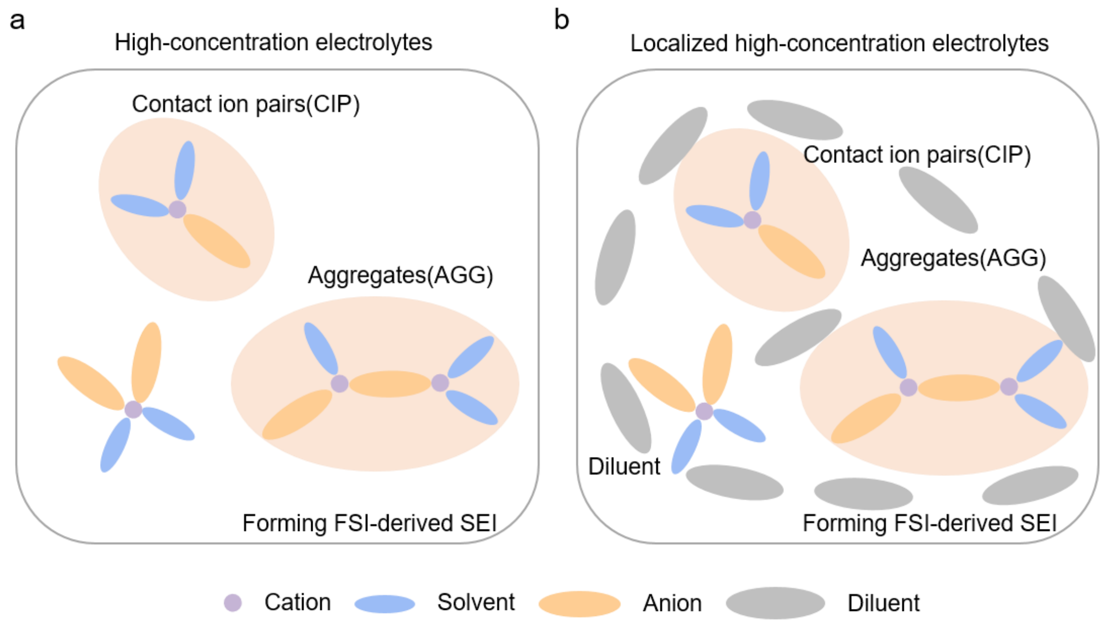


**Supplementary Fig. 38** SEI formation mechanisms on graphite anodes in high-concentration electrolytes (a) and localized high-concentration electrolytes (b).

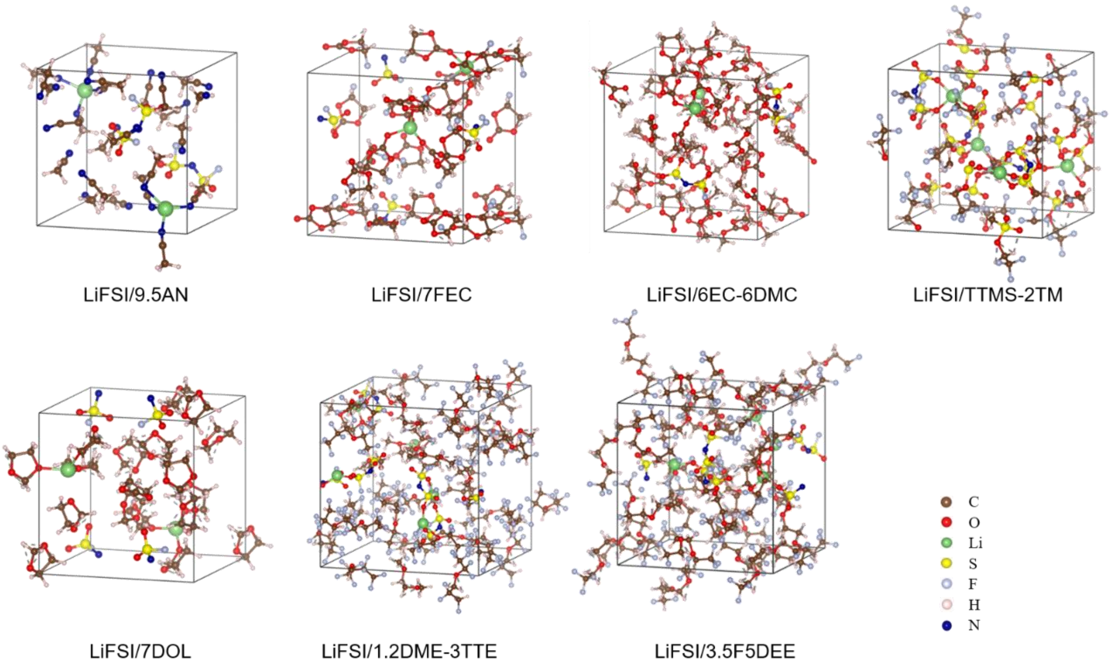


**Supplementary Fig. 39** The *ab initio* molecular dynamics (AIMD) simulation snapshots of different electrolytes.

The corresponding size of simulation electrolyte box and the number of solvents/lithium salts were shown in Supplementary Table 8.

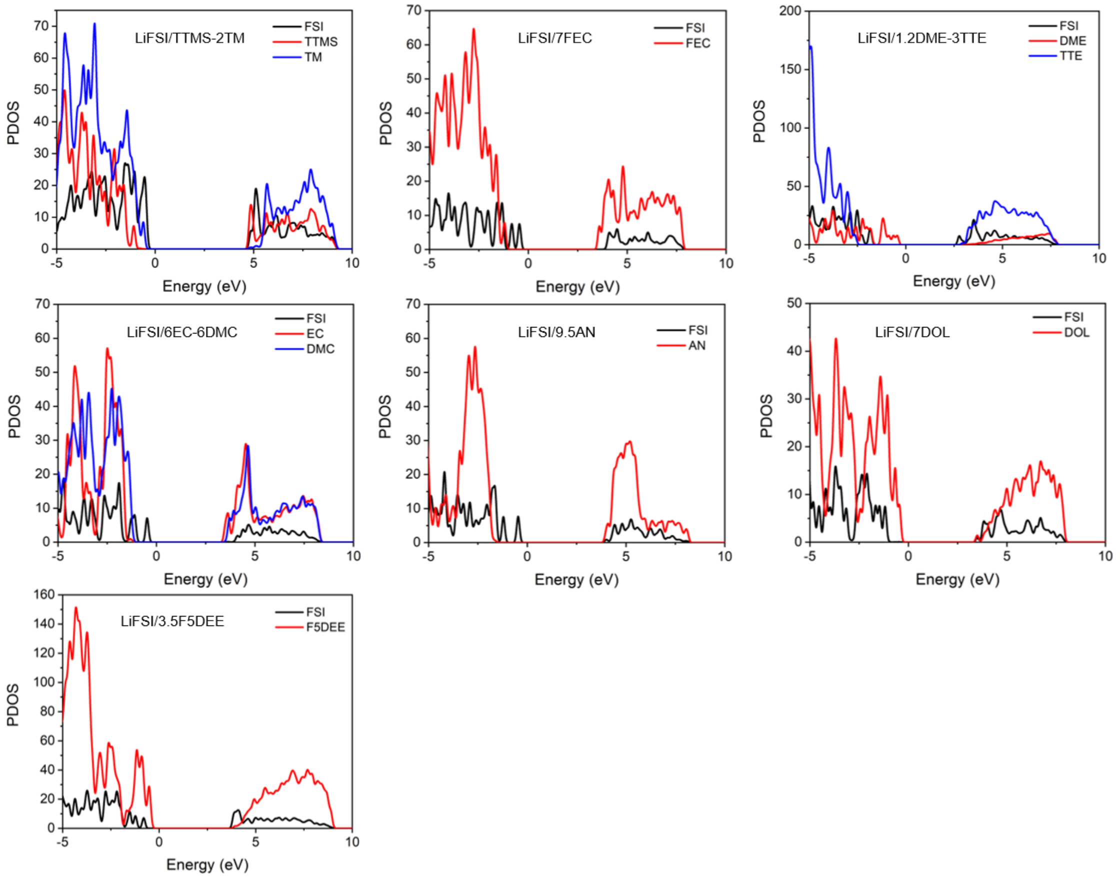


**Supplementary Fig. 40** Projected density of states (PDOS) of different electrolytes.

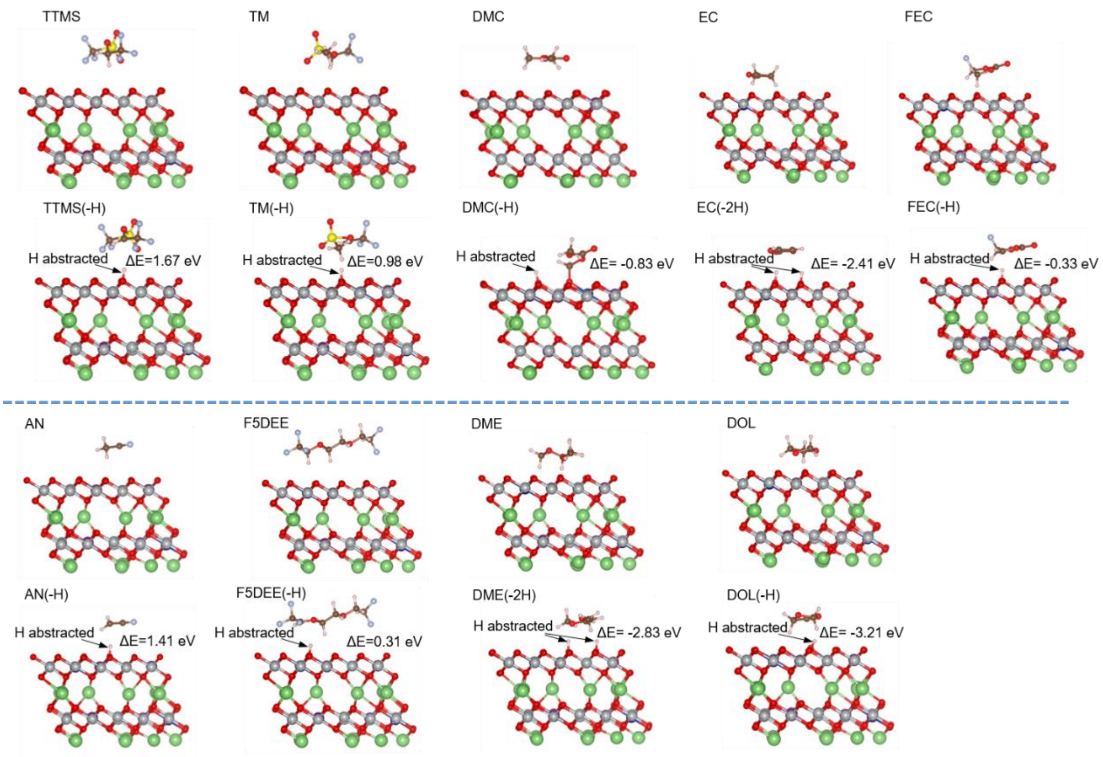


**Supplementary Fig. 41** Reactivity of different solvents at fully charged NCM811 surface.

The solvents with spontaneous H-abstraction on the NCM811 surface enable a negative reaction energy. Among these solvents, EC and DME transfer 2 H, while other solvents transfer 1 H. The radicals formed after one H is abstracted from molecules such as FEC, are longer-lived because they decompose with a high reaction energy for the ring-opening to induce the second H abstraction of FEC•(-H)[1,2]. In contrast, the EC•(-H) radical, which is formed after one H is abstracted from EC molecule, decomposes with a low reaction energy compared to FEC•(-H)[2,3] to participate in the second H abstraction. Therefore, 2 H were abstracted together. Besides, Borodin et al.[4] also reported a spontaneous concerted double proton abstraction reaction for the EC from a converged structure relaxation. DME is also in this case[5].

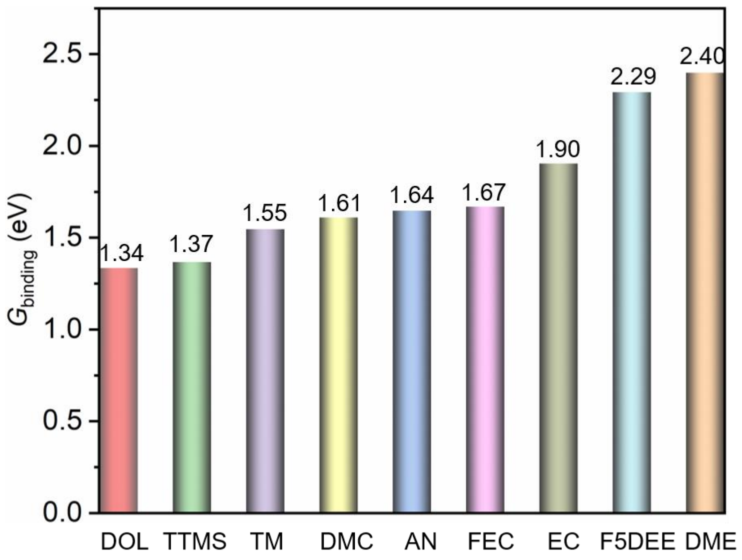


**Supplementary Fig. 42** Binding energies of different solvents with $Li^+$.

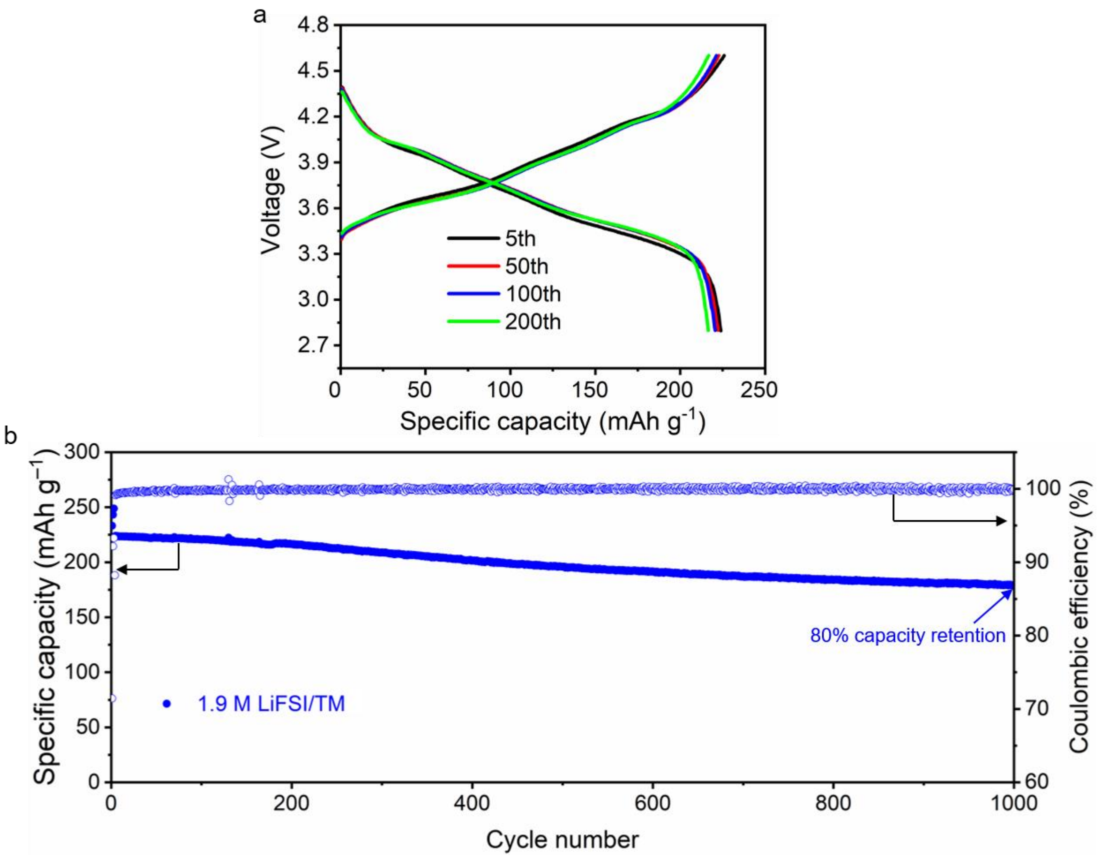


**Supplementary Fig. 43** Charge-discharge curves (a) and cycling performance (b) of the graphite||NCM811 cell using 1.9 M LiFSI/TM electrolyte at 1 C charge and 2 C discharge in the voltage range from 2.8 to 4.6 V.

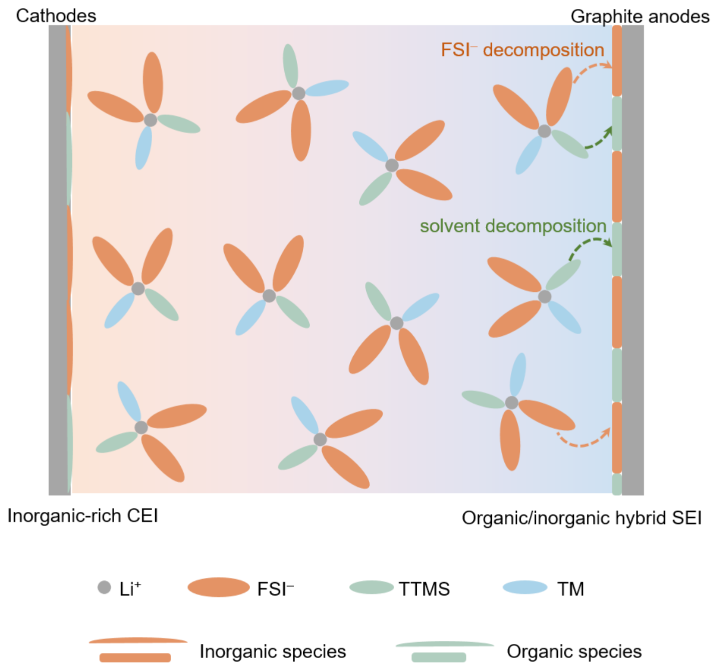


**Supplementary Fig. 44** Schematic illustration of SEI/CEI layer formed in the 1.9 M LiFSI/TTMS-TM electrolyte.

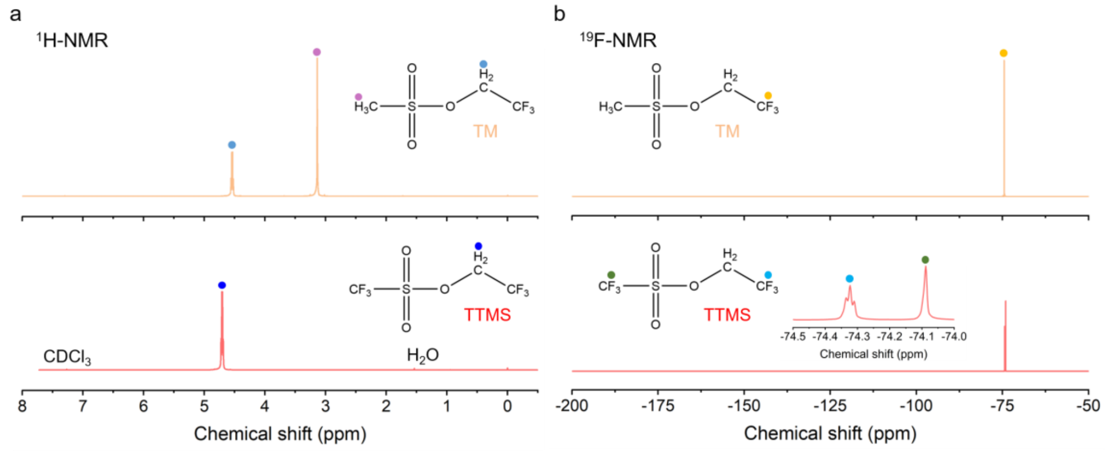


**Supplementary Fig. 45** The $^{1}H$ (a) and $^{19}F$ (b) NMR spectra for TTMS and TM solvents.

**Supplementary Table 1** The determined electrode resistance before and after polarization ($R_0$ and $R_s$), the current in the initial state and steady state ($I_0$ and $I_s$), and the calculated $Li^+$ transference number.

| Electrolytes | Polarizing voltage (ΔV)/V | $R_0/\Omega$ | $R_s/\Omega$ | $I_0$/A | $I_s$/A | Transference number |
|---|---|---|---|---|---|---|
| 1 M $LiPF_6$/EC-DMC | 0.01 | 148.7 | 207.8 | $3.04\times10^{-5}$ | $1.29\times10^{-5}$ | 0.32 |
| 1.9 M LiFSI/TTMS-TM | 0.01 | 34.1 | 38.6 | $1.01\times10^{-4}$ | $6.48\times10^{-5}$ | 0.56 |

**Supplementary Table 2** Element contents on the graphite anodes retrieved from graphite||NCM811 cells after 100 cycles in different electrolytes analyzed by XPS results in Supplementary Fig. 17.

| Elements | Atomic ratio (%) | |
|---|---|---|
| | 1 M $LiPF_6$/EC-DMC | 1.9 M LiFSI/TTMS-TM |
| Ni | 0.25 | 0.06 |
| Mn | 0.09 | 0.05 |
| Co | 0.24 | 0.05 |
| C | 64.99 | 62.13 |
| O | 15 | 11.4 |
| F | 15.27 | 7.39 |
| P | 4.16 | -- |
| N | -- | 14.18 |
| S | -- | 4.74 |

**Supplementary Table 3** Comparison of our work with recent electrolyte works on Li (or graphite)||NCM batteries.

| Electrolyte Composition | Anode||Cathode | Cut-off voltage | Specific capacity | Cyclability | | References |
|---|---|---|---|---|---|---|
| | | | | Coin cells | Pouch cells | |
| 1.9 M LiFSI/TTMS–TM | Gr||NCM**811** | 4.6 V | ~225 mAh $g^{-1}$ | 85% (2002 cycles) | 83% (1000 cycles) | This work |
| 0.8 M LiFSI-0.1 M LiTFSI-0.6 M $LiPF_6$/EMC | Gr||NCM**811** | 4.5 V | ~ | | 82% (200 cycles) | 6 |
| 1.57 M LiFSI in DME-TTE-FEC | Gr||NCM**811** | 4.4 V | 194 mAh $g^{-1}$ | 87% (500 cycles) | | 7 |
| 1.2 M LiFSI in TEPa-EC-BTFE | Gr||NCM**811** | 4.3 V | 197 mAh $g^{-1}$ | 85% (300 cycles) | | 8 |
| 1 M $LiPF_6$ in EC/EMC + 0.5% TPPO | Gr||NCM**811** | 4.3 V | ~198 mAh $g^{-1}$ | 92% (100 cycles) | | 9 |
| Baseline electrolyte + 1.5% LiDFOB | Gr||NCM**811** | 4.3 V | ~198 mAh $g^{-1}$ | 83% (200 cycles) | | 10 |
| 1 M $LiPF_6$ in EC/DMC + 1% TMPSi + 1% VC | Gr||NCM**811** | 4.2 V | 180 mAh $g^{-1}$ | 91% (200 cycles) | | 11 |
| 1 M $LiPF_6$ in FEC/FEMC | Gr||NCM523 | 4.7 V | ~187 mAh $g^{-1}$ | 81% (200 cycles) | | 12 |
| 1.2 M $LiPF_6$ in EC/EMC +TTFP | Gr||NCM523 | 4.6 V | 222 mAh $g^{-1}$ | 86% (50 cycles) | | 13 |
| 1 M $LiPF_6$ in FEC/HFDEC+1% LiDFOB | Gr||NCM523 | 4.6 V | 220 mAh $g^{-1}$ | 82% (100 cycles) | | 14 |
| 1 M $LiPF_6$ in EC/DEC + 0.5% TMST | Gr||NCM111 | 4.5 V | ~180 mAh $g^{-1}$ | 80% (215 cycles) | | 15 |
| 1 M $LiPF_6$ in EC/DEC + 2% LiDMSI | Gr||NCM111 | 4.5 V | ~158 mAh $g^{-1}$ | 85% (100 cycles) | | 16 |
| 1 M $LiPF_6$ in EC/EMC + 2% TBB | Gr||NCM622 | 4.5 V | ~180 mAh $g^{-1}$ | | 90% (100 cycles) | 17 |
| 1 M $LiPF_6$ in EC/EMC + 1% CPE | Gr||NCM622 | 4.5 V | ~180 mAh $g^{-1}$ | | 82% (50 cycles) | 18 |
| 1 M $LiPF_6$ in EC/DMC + 0.1 mg $mL^{-1}$ S | Gr||NCM523 | 4.5 V | 167 mAh $g^{-1}$ | 78% (200 cycles) | | 19 |
| 1 M $LiPF_6$ in EC/EMC + 1% $Li_2CO_3$ | Gr||NCM523 | 4.5 V | 190 mAh $g^{-1}$ | 88% (60 cycles) | | 20 |

| | | | | | | |
|---|---|---|---|---|---|---|
| 1 M $LiPF_6$ in EC/EMC/DEC+1% NOB | Gr\|\|NCM523 | 4.5 V | ~178 mAh $g^{-1}$ | | 73% (100 cycles) | 21 |
| 1 M $LiPF_6$ in EC/EMC + 1% D-DTD | Gr\|\|NCM523 | 4.4V | ~ | | 89% (150cycles) | 22 |
| 1 M $LiPF_6$ in EC/EMC + 0.5% AND | Gr\|\|NCM523 | 4.4V | ~ | 85% (120 cycles) | | 23 |
| 1 m LiFSI in DMTMSA | Li\|\|NCM811 | 4.7 V | 231 mAh $g^{-1}$ | 88% (100 cycles) | | 24 |
| 1 M $LiBF_4$ in SN/FEC | Li\|\|NCM523 | 4.7 V | ~210 mAh $g^{-1}$ | 74% (100 cycles) | | 25 |
| 3.1 M $LiPF_6$ in EC/EMC | Li\|\|NCM811 | 4.6 V | ~220 mAh $g^{-1}$ | 77% (500 cycles) | | 26 |
| 10 M LiFSI in DMC | Li\|\|NCM622 | 4.6 V | ~225 mAh $g^{-1}$ | 86% (100 cycles) | | 27 |
| 8.67 m $LiBF_4$ in DMC | Li\|\|NCM523 | 4.6 V | 200 mAh $g^{-1}$ | 92% (50 cycles) | | 28 |
| 1 M $LiBF_4$ in EC-DMC | Li\|\|NCM333 | 4.6 V | ~164 mAh $g^{-1}$ | 96% (50 cycles) | | 29 |
| 1 M $LiPF_6$ in EC/EMC/DEC +1% TIB | Li\|\|NCM622 | 4.5 V | 198 mAh $g^{-1}$ | 83% (300 cycles) | | 30 |
| 1 M $LiPF_6$ in EC/EMC/DEC +0.5% IPTS | Li\|\|NCM622 | 4.5 V | 215 mAh $g^{-1}$ | 73% (150 cycles) | | 31 |
| 1 M $LiPF_6$ in EC/EMC +0.25% DODSi | Li\|\|NCM811 | 4.5 V | 200 mAh $g^{-1}$ | 66% (100 cycles) | | 32 |
| 1 M $LiPF_6$ in EC/DEC+0.4% Al(EtO)+5% FEC | Li\|\|NCM811 | 4.5 V | 200 mAh $g^{-1}$ | 80% (130 cycles) | | 33 |
| 1 M $LiPF_6$ in FEC/FEMC/HFE | Li\|\|NCM811 | 4.4 V | ~200 mAh $g^{-1}$ | 90% (450 cycles) | | 2 |
| 1.2 M LiFSI in TEP/BTFE | Li\|\|NCM622 | 4.4 V | ~185 mAh $g^{-1}$ | | 90% (450 cycles) | 34 |
| 4 M LiTFSI+0.5 M LiDFOB in FEC/DMC | Li\|\|$LiNi_{0.7}Co_{0.15}Mn_{0.15}O_2$ | 4.3 V | ~170 mAh $g^{-1}$ | 86% (200 cycles) | | 35 |

**Supplementary Table 4** Comparison of our work with recent electrolytes works on Li (or graphite)||LCO batteries.

| Electrolyte Composition | Anode‖Cathode | Cut-off voltage | Specific capacity | Cyclability | | References |
|---|---|---|---|---|---|---|
| | | | | Coin cells | Pouch cells | |
| 1.9 M LiFSI/TTMS–TM | Gr‖LCO | 4.55 V | ~210 mAh $g^{-1}$ | 89% (5329 cysles) | 80% (1400 cycles) | This work |
| 1 M $LiPF_6$ in EC/DEC/EMC + 3% AND + 1% THFPB+0.1% CHB | Gr‖LCO | 4.5 V | ~175 mAh $g^{-1}$ | | 60% (100 cycles) | 36 |
| 1 M $LiPF_6$ in EC/EMC + 0.5% MMDS | Gr‖LCO | 4.5 V | ~ | 70% (150 cycles) | | 37 |
| 1 M $LiPF_6$ in FEC/FEMC/TTE+2% TMSB | Gr‖LCO | 4.5 V | ~225 mAh $g^{-1}$ | 80% (500 cycles) | 78% (200 cycles) | 38 |
| 1 M $LiPF_6$ in EC/DMC/DEC+0.5% DMSE | Gr‖LCO | 4.5 V | ~202 mAh $g^{-1}$ | 70% (150 cycles) | | 39 |
| 1 M $LiPF_6$ in EC/EMC + 1% PDTD | Gr‖LCO | 4.45 V | ~225 mAh $g^{-1}$ | | 68% (150 cycles) | 40 |
| Base electrolyte + 1% SUN+1% HTCN | Gr‖LCO | 4.45 V | ~ | | 80% (140 cycles) | 41 |
| 1 M $LiPF_6$–EC/EMC+ 1% DMFA | Gr‖LCO | 4.45 V | ~ | | 93% (100 cycles) | 42 |
| 1 M $LiPF_6$ in EC/DFSM$_2$/EMC + 5%FEC | Gr‖LCO | 4.4 V | ~165 mAh $g^{-1}$ | 93% (135 cycles) | | 43 |
| 1 M $LiPF_6$–EC/DEC+ 0.1% DPDS | Gr‖LCO | 4.4 V | ~160 mAh $g^{-1}$ | | 95% (200 cycles) | 44 |
| 1 M $LiPF_6$–EC/DMC/EMC+ 0.2% TPPA | Gr‖LCO | 4.4 V | ~164 mAh $g^{-1}$ | 95% (200 cycles) | | 45 |
| 1 M $LiPF_6$–EC/EMC+ 1% HBAG | Gr‖LCO | 4.4 V | ~164 mAh $g^{-1}$ | 95% (160 cycles) | | 46 |
| 1 M $LiPF_6$–EC/EMC+ 1% PTSI | Gr‖LCO | 4.4 V | ~172 mAh $g^{-1}$ | | 95% (200 cycles) | 47 |
| 1 M $LiPF_6$–EC/DMC+ 5% FCPN | Gr‖LCO | 4.3 V | ~158 mAh $g^{-1}$ | 95% (50 cycles) | | 48 |
| 1 M $LiPF_6$–EC/DMC+ 1.6 wt% $LiPO_2F_2$ | Gr‖LCO | 4.3 V | ~148 mAh $g^{-1}$ | 89% (2400 cycles) | | 49 |
| 1 M $LiPF_6$–EC/EMC+ 0.5% MMDS | Gr‖LCO | 4.2 V | ~142 mAh $g^{-1}$ | 85% (150 cycles) | | 37 |
| 1 M $LiPF_6$ in FEC/EMC +3% $LiNO_3$+1% TPFPB | Li‖LCO | 4.6 V | ~205 mAh $g^{-1}$ | 90% (160 cycles) | | 50 |
| 1 m LiFSI in DMCF$_3$SA | Li‖LCO | 4.6 V | ~219 mAh | 89% (200 | | 51 |

| | | | | | | |
|---|---|---|---|---|---|---|
| | | | $g^{-1}$ | cycles) | | |
| 1 M $LiPF_6$ in EC/DEC+0.1% TFPB | Li\|\|LCO | 4.6 V | ~205 mAh $g^{-1}$ | 85% (200 cycles) | | 52 |
| 1 M $LiPF_6$ in EC/DEC+0.1% KSeCN | Li\|\|LCO | 4.6 V | ~205 mAh $g^{-1}$ | 69% (750 cycles) | | 53 |
| 1 M $LiPF_6$ in FEC/DMC/HFE +10% MSM | Li\|\|LCO | 4.55 V | ~195 mAh $g^{-1}$ | 75% (300 cycles) | | 54 |
| 1.2 M $LiPF_6$ in FEC/DMC/HFE | Li\|\|LCO | 4.5 V | ~184 mAh $g^{-1}$ | 84% (300 cycles) | | 55 |
| LiFSI-1 DME-3 TTE (mol ratio) | Li\|\|LCO | 4.5 V | ~184 mAh $g^{-1}$ | 93% (300 cycles) | | 56 |
| 1 M $LiPF_6$ in EC/EMC/DEC + FN | Li\|\|LCO | 4.5 V | ~188 mAh $g^{-1}$ | 96% (120 cycles) | | 57 |
| 1 M $LiPF_6$ in EC/DMC + PPFPN | Li\|\|LCO | 4.5 V | ~185 mAh $g^{-1}$ | 90% (300 cycles) | | 58 |
| 1 M $LiPF_6$ in EC/EMC/DEC+0.5%TBB | Li\|\|LCO | 4.4 V | ~160 mAh $g^{-1}$ | 83% (120 cycles) | | 59 |
| 1 M $LiPF_6$ in EC/EMC/DEC+0.5% LBTB | Li\|\|LCO | 4.4 V | ~162 mAh $g^{-1}$ | 73% (300 cycles) | | 60 |

**Supplementary Table 5** Comparison of our work with recent doping or surface coating works on Li (or graphite)||LCO batteries.

| Doping or surface coating | Anode\|\|Cathode | Cut-off voltage | Specific capacity | Cyclability | | References |
|---|---|---|---|---|---|---|
| | | | | Coin cells | Pouch cells | |
| 1.9 M LiFSI/TTMS–TM (without doping or coating) | Gr\|\|LCO | 4.55 V | ~210 mAh $g^{-1}$ | 89% (5329 cysles) | 80% (1400 cysles) | This work |
| $LiAlO_2$-LCO | Li\|\|LCO | 4.6 V | ~199 mAh $g^{-1}$ | 87% (50 cycles) | | 61 |
| Ti–Mg–Al-LCO | Li\|\|LCO | 4.6 V | ~202 mAh $g^{-1}$ | | 86% (100 cycles) | 62 |
| $Li_{1/3}Al_{1/3}Co_{2/3}O_{4/3}F_{2/3}$-LCO | Li\|\|LCO | 4.6 V | ~209 mAh $g^{-1}$ | 82% (200 cycles) | | 63 |
| $Li_{1.5}Al_{0.5}Ti_{1.5}(PO_4)_3$-LCO | Li\|\|LCO | 4.6 V | ~218 mAh $g^{-1}$ | 88% (100 cycles) | | 64 |
| $LiAlPO_{3.93}F_{1.07}$-LCO | Li\|\|LCO | 4.55 V | ~210 mAh $g^{-1}$ | 92% (50 cycles) | | 65 |
| $Al_2O_3$-LCO | Li\|\|LCO | 4.5 V | ~178 mAh $g^{-1}$ | 83% (500 cycles) | | 66 |
| $Li_{1.4}Al_{0.4}Ti_{1.6}(PO_4)_3$-LCO | Li\|\|LCO | 4.5 V | ~180 mAh $g^{-1}$ | 93% (50 cycles) | | 67 |
| $Li_2CO_3$-LCO | Li\|\|LCO | 4.5 V | ~178 mAh $g^{-1}$ | 88% (60 cycles) | | 68 |

**Supplementary Table 6** Parameters of graphite||NCM811 pouch cells with 306 Wh $kg^{-1}$ at 1-Ah level and 338 Wh $kg^{-1}$ at 10-Ah level.

| Graphite\|\|NCM811 | Parameter | Value | |
|---|---|---|---|
| | | 1 Ah-level | 10 Ah-level |
| NCM811 cathode | Discharge capacity | 230 mAh $g^{-1}$ | 230 mAh $g^{-1}$ |
| | Active material loading | 95.5% | 95.5% |
| | Press density | 3.4 g $cc^{-1}$ | 3.4 g $cc^{-1}$ |
| | Areal weight | 14.24 mg $cm^{-2}$ | 14.24 mg $cm^{-2}$ |
| | Number of layers | 11 | 27 |
| | Total weight | 4.93 g | 46.13 g |
| AG | Discharge capacity | 372 mAh $g^{-1}$ | 372 mAh $g^{-1}$ |
| | Active material loading | 94.8% | 94.8% |
| | Press density | 1.5 g $cc^{-1}$ | 1.5 g $cc^{-1}$ |
| | Areal weight | 9 mg $cm^{-2}$ | 9.9 mg $cm^{-2}$ |
| | N/P ratio | 1.01 | 1.12 |
| | Total weight | 3.1 g | 32.2 g |
| Al current | Thickness | 8 μm | 8 μm |
| | Total weight | 0.4 g | 3.63 g |
| Cu current | Thickness | 6 μm | 6 μm |
| | Total weight | 1.01 g | 9.0 g |
| Electrolyte | E/C ratio | 2 g $(Ah)^{-1}$ | 2 g $(Ah)^{-1}$ |
| | Total weight | 2 g | 20 g |
| Separator | Total weight | 0.0005 g | 0.005 g |
| Tab | Total weight | 0.1 g | 0.1 g |
| Package | Total weight | 1.15 g | 4.39 g |
| Cell | Average voltage | 3.9 V | 3.9 V |
| | Energy density | 306 Wh $kg^{-1}$ | 338 Wh $kg^{-1}$ |

Note: Energy density calculation method:

1 Ah-level: The total weight of graphite||NCM811 pouch cell is 12.7 g. The average output voltage is 3.9 V. The calculated energy density of the cell is 306 Wh $kg^{-1}$ (=1 Ah*3.9 V/ 0.0127 kg).

10 Ah-level: The total weight of graphite||NCM811 pouch cell is 115.5 g. The average output voltage is 3.9 V. The calculated energy density of the cell is 338 Wh $kg^{-1}$ (=10 Ah*3.9 V/ 0.1155 kg).

**Supplementary Table 7** Parameters of graphite||LCO pouch cells with 313 Wh $kg^{-1}$ at 1-Ah level and 363 Wh $kg^{-1}$ at 10-Ah level.

| Graphite\|\|LCO | Parameter | Value | |
|---|---|---|---|
| | | 1 Ah-level | 10 Ah-level |
| LCO cathode | Discharge capacity | 210 mAh $g^{-1}$ | 210 mAh $g^{-1}$ |
| | Active material loading | 98.6% | 98.6% |
| | Press density | 4.1 g $cc^{-1}$ | 4.1 g $cc^{-1}$ |
| | Areal wight | 18.7 mg $cm^{-2}$ | 18.7 mg $cm^{-2}$ |
| | Number of layers | 9 | 29 |
| | Total mass | 5.3 g | 48.8 g |
| AG | Discharge capacity | 372 mAh $g^{-1}$ | 372 mAh $g^{-1}$ |
| | Active material loading | 96.7% | 96.7% |
| | Press density | 1.7 g $cc^{-1}$ | 1.7 g $cc^{-1}$ |
| | Areal wight | 12 mg $cm^{-2}$ | 12 mg $cm^{-2}$ |
| | N/P ratio | 1.11 | 1.11 |
| | Total mass | 3.4 g | 31.3 g |
| Al current | Thickness | 8 μm | 8 μm |
| | Total mass | 0.34 g | 2.91 g |
| Cu current | Thickness | 6 μm | 6 μm |
| | Total mass | 0.84 g | 7.23 g |
| Electrolyte | E/C ratio | 2 g $(Ah)^{-1}$ | 2 g $(Ah)^{-1}$ |
| | Total mass | 2 g | 20 g |
| Separator | Total mass | 0.0005 g | 0.002 g |
| Tab | Total mass | 0.1 g | 0.1 g |
| Package | Total mass | 1.15 g | 3.29 g |
| Cell | Average voltage | 4.12 V | 4.12 V |
| | Energy density | 313 Wh $kg^{-1}$ | 363 Wh $kg^{-1}$ |

Note: Energy density calculation method:

1 Ah-level: The total weight of graphite||LCO pouch cell is 13.1 g. The average output voltage is 4.12 V. The calculated energy density of the cell is 313 Wh $kg^{-1}$ (=1 Ah*4.12 V/ 0.0131 kg).

10 Ah-level: The total weight of graphite||LCO pouch cell is 113.6 g. The average output voltage is 4.12 V. The calculated energy density of the cell is 363 Wh $kg^{-1}$ (=10 Ah*4.12 V/ 0.1136 kg).

**Supplementary Table 8** The size and the number of solvents/lithium salts in the AIMD simulation box of electrolytes.

| Electrolyte system | Size of simulated box (Å) | Number of LiFSI and solvent |
|---|---|---|
| LiFSI/AN | 12.42×12.42×12.42 | 2 LiFSI/19 AN |
| LiFSI/FEC | 14.56×14.56×14.56 | 2 LiFSI/14 FEC |
| LiFSI/EC-DMC | 16.10×16.10×16.10 | 2 LiFSI/12 EC/12 DMC |
| LiFSI/TTMS-TM | 14.52×14.52×14.52 | 4 LiFSI/4 TTMS/8 TM |
| LiFSI/DOL | 13.28×13.28×13.28 | 2 LiFSI/14 DOL |
| LiFSI/DME-TTE | 19.00×19.00×19.00 | 5 LiFSI/6 DME/15 TTE |
| LiFSI/F5DEE | 16.72×16.72×16.72 | 4 LiFSI/14 F5DEE |

## Supplementary references


1. Xia, L. et al. Oxidation decomposition mechanism of fluoroethylene carbonate-based electrolytes for high-voltage lithium ion batteries: a DFT calculation and experimental study. *ChemistrySelect* **2**, 7353-7361 (2017).
2. Fan, X. et al. Non-flammable electrolyte enables Li-metal batteries with aggressive cathode chemistries. *Nat. Nanotechnol.* **13**, 715-722 (2018).
3. Xing, L. et al. Theoretical investigations on oxidative stability of solvents and oxidative decomposition mechanism of ethylene carbonate for lithium ion battery use. *J. Phys. Chem. B* **113**, 16596-16602 (2009).
4. Borodin, O. et al. Towards high throughput screening of electrochemical stability of battery electrolytes. *Nanotechnology* **26**, 354003 (2015).
5. Huang, Y. et al. Eco-friendly electrolytes via a robust bond design for high-energy Li metal batteries. *Energy Environ. Sci.* **15**, 4349-4361 (2022).
6. Wu, Y. et al. High-voltage and high-safety practical lithium batteries with ethylene carbonate-free electrolyte. *Adv. Energy Mater.* **11**, 2102299 (2021).
7. Jia, H. et al. Enabling ether-based electrolytes for long cycle life of lithium-ion batteries at high charge voltage. *ACS Appl. Mater. Inter.* **12**, 54893-54903 (2020).
8. Cao, X. et al. Nonflammable el ectrolytes for lithium ion batteries enabled by ultraconformal passivation interphases. *ACS Energy Lett.* **4**, 2529-2534 (2019).
9. Beltrop, K. et al. Triphenylphosphine oxide as highly effective electrolyte additive for graphite/NMC811 lithium ion cells. *Chem. Mater.* **30**, 2726-2741 (2018).
10. Dong, Q. et al. Insights into the dual role of lithium difluoro(oxalato)borate additive in improving the electrochemical performance of NMC811||graphite cells. *ACS Appl. Energy Mater.* **3**, 695-704 (2019).
11. Vidal Laveda, J. et al. Stabilizing capacity retention in NMC811/graphite full cells via TMSPi electrolyte additives. *ACS Appl. Energy Mater.* **2**, 7036-7044 (2019).
12. Im, J. et al. Fluorinated carbonate-based electrolyte for high-voltage $Li(Ni_{0.5}Mn_{0.3}Co_{0.2})O_2$/graphite lithium-ion battery. *J. Electrochem. Soc.* **164**, A6381-A6385 (2017).
13. He, M. et al. Mechanistic insight in the function of phosphite additives for protection of $LiNi_{0.5}Co_{0.2}Mn_{0.3}O_2$ cathode in high voltage Li-ion cells. *ACS Appl. Mater. Interfaces* **8**, 11450-11458 (2016).
14. He, M. et al. High voltage $LiNi_{0.5}Mn_{0.3}Co_{0.2}O_2$/graphite cell cycled at 4.6 V with a FEC/HFDEC-based electrolyte. *Adv. Energy Mater.* **7**, 1700109 (2017).
15. Imholt, L. et al. Trimethylsiloxy based metal complexes as electrolyte additives for high voltage application in lithium ion cells. *Electrochim. Acta* **235**, 332-339 (2017).
16. Murmann, P. et al. Lithium-cyclo-difluoromethane-1,1-bis(sulfonyl)imide as a stabilizing electrolyte additive for improved high voltage applications in lithium-ion batteries. *Phys. Chem. Chem. Phys.* **17**, 9352-8 (2015).
17. Li, J. et al. Efficiently suppressing oxygen evolution in high voltage graphite/NCM pouch cell with tributyl borate as electrolyte additive. *Electrochim. Acta* **354**, 136722 (2020).
18. Liao, B. et al. Constructing unique cathode interface by manipulating functional groups of electrolyte additive for graphite/$LiNi_{0.6}Co_{0.2}Mn_{0.2}O_2$ cells at high voltage. *J. Phys. Chem.*

*Lett.* **9**, 3434-3445 (2018).
19. Xiong, J. et al. Sulfur is a new high-performance additive toward high-voltage $LiNi_{0.5}Co_{0.2}Mn_{0.3}O_2$ cathode: tiny amount, huge impact. *ACS Appl. Mater. Interfaces* **13**, 18648-18657 (2021).
20. Klein, S. et al. On the beneficial impact of $Li_2CO_3$ as electrolyte additive in NCM523||graphite lithium ion cells under high-voltage conditions. *Adv. Energy Mater.* **11**, 2003756 (2021).
21. Hu, Z. et al. A novel electrolyte additive enables high-voltage operation of nickel-rich oxide/graphite cells. *J. Phys. Chem. Lett.* **12**, 4327-4338 (2021).
22. Yang, T. et al. Sulfur-containing $C_2H_2O_8S_2$ molecules as an overall-functional electrolyte additive for high-voltage $LiNi_{0.5}Co_{0.2}Mn_{0.3}O_2$/graphite batteries with enhanced performance. *J. Power Sources* **470**, 228462 (2020).
23. Han, S. et al. 1,4-dicyanobutane as a film-forming additive for high-voltage in lithium-ion batteries. *Solid State Ion.* **337**, 63-69 (2019).
24. Xue, W. et al. Ultra-high-voltage Ni-rich layered cathodes in practical Li metal batteries enabled by a sulfonamide-based electrolyte. *Nat. Energy* **6**, 495-505 (2021).
25. Zhang, Q. et al. Enhancing the high voltage interface compatibility of $LiNi_{0.5}Co_{0.2}Mn_{0.3}O_2$ in the succinonitrile-based electrolyte. *Electrochim. Acta* **298**, 818-826 (2019).
26. Tatara, R. et al. Enhanced cycling performance of Ni-rich positive electrodes (NMC) in Li-ion batteries by reducing electrolyte free-solvent activity. *ACS Appl. Mater. Interfaces* **11**, 34973-34988 (2019).
27. Fan, X. et al. Highly fluorinated interphases enable high-voltage Li-metal batteries. *Chem* **4**, 174-185 (2018).
28. Doi, T. et al. Fluoroalkyl ether-diluted dimethyl carbonate-based electrolyte solutions for high-voltage operation of $LiNi_{0.5}Co_{0.2}Mn_{0.3}O_2$ electrodes in lithium ion batteries. *Sustain. Energy Fuels* **2**, 1197-1205 (2018).
29. Gallus, D.R. et al. The influence of different conducting salts on the metal dissolution and capacity fading of NCM cathode material. *Electrochim. Acta* **134**, 393-398 (2014).
305. Qin, Z. et al. Triisopropyl borate as an electrolyte additive for improving the high voltage stability of $LiNi_{0.6}Co_{0.2}Mn_{0.2}O_2$ cathode. *J. Electroanal. Chem.* **854**, 113506 (2019).
31. Wang, S. et al. A new additive 3-isocyanatopropyltriethoxysilane to improve electrochemical performance of Li/NCM622 half-cell at high voltage. *J. Power Sources* **423**, 90-97 (2019).
32. Jang, S.H., Jung, K. & Yim, T. Silyl-group functionalized organic additive for high voltage Ni-rich cathode material. *Curr. Appl. Phys.* **18**, 1345-1351 (2018).
33. Zhang, Y. et al. A dual-function liquid electrolyte additive for high-energy non-aqueous lithium metal batteries. *Nat. Commun.* **13**, 1297 (2022).
34. Niu, C. et al. High-energy lithium metal pouch cells with limited anode swelling and long stable cycles. *Nat. Energy* **4**, 551-559 (2019).
35. Wang, W. et al. Stable cycling of high-voltage lithium-metal batteries enabled by high-concentration FEC-based electrolyte. *ACS Appl. Mater. Interfaces* **12**, 22901-22909 (2020).
36. Pang, C. et al. Three-component functional additive in a $LiPF_6$-based carbonate electrolyte for a high-voltage $LiCoO_2$/graphite battery system. *Energy Technol.* **5**, 1979-1989 (2017).
37. Zuo, X. et al. High-voltage performance of $LiCoO_2$/graphite batteries with methylene

methanedisulfonate as electrolyte additive. *J. Power Sources* **219**, 94-99 (2012).
38. Zhang, J. et al. Interfacial design for a 4.6 V high-voltage single-crystalline $LiCoO_2$ Cathode. *Adv. Mater.* **34**, e2108353 (2021).
39. Zheng, X. et al. Di(methylsulfonyl) ethane: new electrolyte additive for enhancing $LiCoO_2$/electrolyte interface stability under high voltage. *ACS Appl. Mater. Interfaces* **11**, 36244-36251 (2019).
40. Wu, S. et al. Stabilizing $LiCoO_2$/graphite at high voltages with an electrolyte additive. *ACS Appl. Mater. Inter.* **11**, 17940-17951 (2019).
41. Yang, X. et al. Enabling stable high-voltage $LiCoO_2$ operation by using synergetic interfacial modification strategy. *Adv. Funct. Mater.* **30**, 2004664 (2020).
42. Xiang, F., Wang, P. & Cheng, H. Methyl 2,2-difluoro-2-(fluorosulfonyl) acetate as a novel electrolyte additive for high-voltage $LiCoO_2$/graphite pouch Li-ion cells. *Energy Technol.* **8**, 1901277 (2020).
43. Wang, J. et al. Fluorosilane compounds with oligo(ethylene oxide) substituent as safe electrolyte solvents for high-voltage lithium-ion batteries. *J. Power Sources* **334**, 58-64 (2016).
44. Park, H. & Kim, H.-J. Diphenyl diselenide as SEI-forming additive for a high-voltage $LiCoO_2$/graphite battery. *J. Electrochem. Soc.* **167**, 070555 (2020).
45. Lee, J.N. et al. N-(triphenylphosphoranylidene) aniline as a novel electrolyte additive for high voltage $LiCoO_2$ operations in lithium ion batteries. *Electrochim. Acta* **56**, 5195-5200 (2011).
46. Lei, W. et al. 4-hydroxy-2-butanesulfonic acid gamma-sultone as a bifunctional electrolyte additive for $LiCoO_2$/graphite batteries with enhanced performances. *ACS Appl. Energy Mater.* **4**, 5877-5887 (2021).
47. Zhang, L. et al. 1-(p-toluenesulfonyl) imidazole (PTSI) as the novel bifunctional electrolyte for $LiCoO_2$-based cells with improved performance at high voltage. *J. Power Sources* **491**, 229596 (2021).
48. Zhang, Q. et al. Improved thermal stability of $LiCoO_2$ by cyclotriphosphazene additives in lithium-ion batteries. *Chem. Lett.* **34**, 1012-1013 (2005).
49. Yang, G. et al. Improving the cyclability performance of lithium-ion batteries by introducing lithium difluorophosphate ($LiPO_2F_2$) additive. *RSC advances* **7**, 26052-26059 (2017).
50. Li, S. et al. Synergistic dual-additive electrolyte enables practical lithium-metal batteries. *Angew. Chem. Int. Ed.* **59**, 14935-14941 (2020).
51. Xue, W. et al. Stabilizing electrode–electrolyte interfaces to realize high-voltage Li||$LiCoO_2$ batteries by a sulfonamide-based electrolyte. *Energy Environ. Sci.* **14**, 6030-6040 (2021).
52. Zou, Y. et al. Stabilizing the $LiCoO_2$ interface at high voltage with an electrolyte additive 2,4,6-tris(4-fluorophenyl)boroxin. *ACS Sustain. Chem. Eng.* **9**, 15042-15052 (2021).
53. Fu, A. et al. Synergistical stabilization of Li metal anodes and $LiCoO_2$ cathodes in high-voltage Li||$LiCoO_2$ batteries by potassium selenocyanate (KSeCN) additive. *ACS Energy Lett.* **7**, 1364-1373 (2022).
54. Kong, X., Zhou, R., Wang, J. & Zhao, J. An effective electrolyte strategy to improve the high-voltage performance of $LiCoO_2$ cathode materials. *ACS Appl. Energy Mater.* **2**, 4683-4691 (2019).

55. Lin, S. & Zhao, J. Functional electrolyte of fluorinated ether and ester for stabilizing both 4.5 V $LiCoO_2$ cathode and lithium metal anode. *ACS Appl. Mater. Interfaces* **12**, 8316-8323 (2020).
56. Ren, X. et al. Designing advanced in situ electrode/electrolyte interphases for wide temperature operation of 4.5 V Li||$LiCoO_2$ batteries. *Adv. Mater.* **32**, e2004898 (2020).
57. Wang, X. et al. Maintaining structural integrity of 4.5 V lithium cobalt oxide cathode with fumaronitrile as a novel electrolyte additive. *J. Power Sources* **338**, 108-116 (2017).
58. Ji, Y. et al. Toward a stable electrochemical interphase with enhanced safety on high-voltage $LiCoO_2$ cathode: a case of phosphazene additives. *J. Power Sources* **359**, 391-399 (2017).
59. Qin, Z. et al. Tributyl borate as a novel electrolyte additive to improve high voltage stability of lithium cobalt oxide in carbonate-based electrolyte. *Electrochim. Acta* **276**, 412-416 (2018).
60. Sun, Z. et al. Design of a novel electrolyte additive for high voltage $LiCoO_2$ cathode lithium-ion batteries: lithium 4-benzonitrile trimethyl borate. *J. Power Sources* **503**, 230033 (2021).
61. Xie, J. et al. Engineering the surface of $LiCoO_2$ electrodes using atomic layer deposition for stable high-voltage lithium ion batteries. *Nano Res.* **10**, 3754-3764 (2017).
62. Zhang, J.-N. et al. Trace doping of multiple elements enables stable battery cycling of $LiCoO_2$ at 4.6 V. *Nat. Energy* **4**, 594-603 (2019).
63. Qian, J. et al. Electrochemical surface passivation of $LiCoO_2$ particles at ultrahigh voltage and its applications in lithium-based batteries. *Nat. Commun.* **9**, 4918 (2018).
64. Wang, Y. et al. An in situ formed surface coating layer enabling $LiCoO_2$ with stable 4.6 V high-voltage cycle performances. *Adv. Energy Mater.* **10**, 2001413 (2020).
65. Shen, B. et al. Mixed lithium ion and electron conducting $LiAlPO_{3.93}F_{1.07}$-coated $LiCoO_2$ cathode with improved electrochemical performance. *Electrochem. Commun.* **83**, 106-109 (2017).
66. Zhou, A. et al. $Al_2O_3$ surface coating on $LiCoO_2$ through a facile and scalable wet-chemical method towards high-energy cathode materials withstanding high cutoff voltages. *J. Mater. Chem. A* **5**, 24361-24370 (2017).
67. Yang, Q. et al. Surface-protected $LiCoO_2$ with ultrathin solid oxide electrolyte film for high-voltage lithium ion batteries and lithium polymer batteries. *J. Power Sources* **388**, 65-70 (2018).
68. Dai, X. et al. Extending the high-voltage capacity of $LiCoO_2$ cathode by direct coating of the composite electrode with $Li_2CO_3$ via magnetron sputtering. *J. Phys. Chem. C* **120**, 422-430 (2015).
