# Supplementary material for: Multifunctional solvent molecule design enables high-voltage Li-ion batteries
Source: Nat Commun. 2023 Apr 18;14:2211. doi: 10.1038/s41467-023-37999-4 (PMC10113204; doi:10.1038/s41467-023-37999-4)
Supplement: Supplementary file 2 — Description of Additional Supplementary Files [file 41467_2023_37999_MOESM2_ESM.pdf]

Description of Additional Supplementary Items

Title: Supplementary Movie 1:
Description: Nail penetration test for carbonate electrolyte

Title: Supplementary Movie 2:
Description: Nail penetration test for our electrolyte

Title: Supplementary Movie 3:
Description: Flame retardancy test for our electrolyte

Title: Supplementary Movie 4:
Description: Flame retardancy test for carbonate electrolyte
